# Bifurcated Fluorotelomer Carboxylic Acid Biotransformation: Enoyl-Hydration versus Hydroxylation Regulated by Redox Conditions

**DOI:** 10.1021/acs.est.6c04089

**Published:** 2026-09-22

**Authors:** Boyuan Su, Hao Chen, John Washington, Mengyan Li

**Affiliations:** † Department of Chemistry and Environmental Science, 5965 New Jersey Institute of Technology, Newark, New Jersey 07102, United States; ‡ Department of Geology, University of Georgia, Athens, Georgia 30602, United States; § U.S. Environmental Protection Agency, Athens, Georgia 30605, United States

**Keywords:** fluorotelomer carboxylic acids, PFAS precursors, bifurcated biotransformation, redox conditions, biodefluorination, activated sludge, biosolids, nontarget analysis

## Abstract

Fluorotelomer carboxylic acids (FTCAs) are important precursors of perfluoroalkyl carboxylic acids (PFCAs) in wastewater and biosolids, yet their redox-dependent biotransformation remains poorly understood. We investigated 5:3 FTCA biotransformation in microcosms inoculated with activated sludge and anaerobic digester sludge under aerobic, nitrate-reducing, sulfate-reducing, and methanogenic conditions. After correction for abiotic losses, the pseudo-first-order removal rate was 0.074 d^–1^ under aerobic conditions, over an order of magnitude higher than under nitrate-reducing (0.0045 d^–1^) and methanogenic (0.0028 d^–1^) conditions; sulfate-reducing conditions showed minimal removal. Nontarget Nano-ESI-HRMS analysis further revealed ultrashort-chain PFCAs, including PFPrA, and previously unreported intermediates, including keto-5:3 FTCA and 3-F-4:3 FTCA. A bifurcated FTCA biotransformation framework was unveiled, initiated by Cα-centered hydroxylation and Cβ-centered enoyl-hydration. Hydroxylation promoted iterative CF_2_ moiety removal and progressive chain shortening, accounting for nearly 95% of measured products and 72.8% of fluoride release under aerobic conditions, whereas enoyl-hydration dominated (>60%) under nitrate reduction and favored PFCA formation. These findings provide quantitative and mechanistic evidence for competing FTCA biotransformation routes in wastewater microbiomes and highlight redox control as a critical lever for steering PFAS fate toward either PFCA accumulation or progressive defluorination.

## Graphical abstract

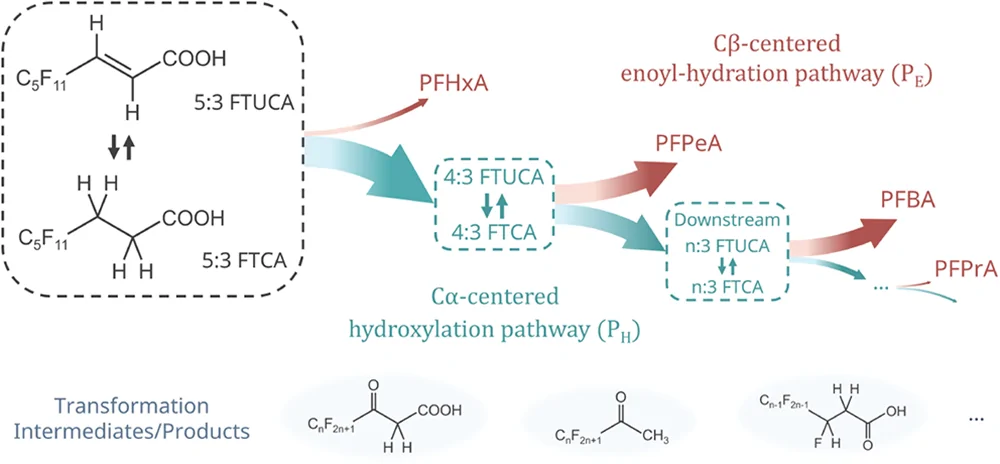

## 1 Introduction

Per- and polyfluoroalkyl substances (PFAS), known as “forever chemicals,” comprise a large and structurally diverse class of synthetic compounds characterized by exceptional chemical stability, environmental persistence, mobility, and bioaccumulation potential.^1,2^ Mounting evidence links PFAS exposure to adverse human health outcomes, prompting growing concern over their widespread occurrence in surface and groundwater, landfill leachates, wastewater treatment residuals, soils, and biota.^3–12^ Regulatory attention has historically focused on legacy perfluoroalkyl acids (PFAAs), such as perfluorooctanoic acid (PFOA) and perfluorooctanesulfonate (PFOS), culminating in progressively more stringent drinking water and product standards under international frameworks including the Stockholm Convention.^13,14^

In contrast, polyfluoroalkyl precursors, particularly fluorotelomers (FTs), remain prevalent in commercial formulations and modern technological products and constitute long-term environmental reservoirs of PFAS contamination.^15–19^ This regulatory shift is reflected in the European Union (EU)’s recent Regulation 2024/2462 on “PFHxA, its salts, and PFHxA-related substances”, which explicitly defines restricted compounds by the presence of linear or branched perfluoropentyl (C_5_F_11_^–^) or perfluorohexyl (C_6_F_13_^–^) moieties.^20^ By extending regulation from fully fluorinated end products to polyfluorinated precursors, this framework directly implicates fluorotelomer carboxylic acids (FTCAs), particularly 5:3 and 6:2 FTCAs, were under increasing monitoring scrutiny. Accordingly, FTCAs are now included in routine monitoring programs, such as EPA Method 1633 ^21^ and California’s biomonitoring initiatives,^22^ and are under consideration in ongoing PFAS restriction efforts under EU’s REACH.^23^

Among FT precursors, 5:3 FTCA has emerged as a compound of particular concern due to its high environmental abundance^24,25^ as well as a prominent transformation intermediate of more complex precursors.^26,27^ Surveys of landfill leachates have reported 5:3 FTCA as one of the dominant PFAS species, accounting for up to 71.1% of total measured PFAS mass.^28,29^ Such leachates and other 5:3 FTCA-rich waste streams are frequently cotreated in municipal wastewater treatment plants (WWTPs), resulting in the consistent detection of 5:3 FTCA throughout treatment trains, spanning from influents and activated sludge to secondary clarifiers and final effluents.^30^ Recent studies further indicate that 5:3 FTCA partitions substantially between aqueous and solid phases, enabling biosolids to act as secondary reservoirs and potential sources upon land application.^31–33^ Compounding these concerns, toxicological studies suggest that FTCAs can exhibit acute aquatic toxicity several orders of magnitude greater than their corresponding perfluoroalkyl carboxylic acids (PFCAs).^34^ Together, these observations underscore the importance of understanding 5:3 FTCA biotransformation in WWTPs as a determinant of downstream PFAS release and environmental exposure.

Biotransformation of 5:3 FTCA has generally been interpreted through two mechanistic paradigms. The first views 5:3 FTCA as a fatty acid analogue that undergoes β-oxidation or β-oxidation-like reactions, ultimately producing perfluorohexanoic acid (PFHxA) while preserving the perfluoroalkyl tail. In classical β-oxidation, activation by coenzyme A (CoA) is an essential first step, followed by sequential dehydrogenation, hydration, oxidation, and thiolytic cleavage of two nonfluorinated carbons.^35,36^ Although CoA conjugates have been identified for shorter-chain n:3 FTCAs (e.g., 2:3 and 3:3 FTCAs), evidence for CoA activation of 5:3 FTCA and other longer chain FTCAs remains limited.^37,38^ A recent study further demonstrated that an acyl-CoA synthetase from *Dietzia aurantiaca* strain J3 catalyzed CoA conjugation of C8 fluorotelomer unsaturated carboxylic acids (FTUCAs) (e.g., 6:2 FTUCA) but not 5:3 FTCA or 6:2 FTCA, suggesting that classical β-oxidation may not fully explain 5:3 FTCA transformation.^39^ Nevertheless, numerous studies have reported β-hydroxylated intermediates and related nonconjugative transformation products (TPs) that resemble portions of the β-oxidation sequence, leading to the broader concept of a β-oxidation-like pathway associated with PFCA formation.^40–43^

A second paradigm emphasizes iterative “one-carbon removal”, in which n:3 FT­(U)­CAs undergo successive oxidative chain-shortening reactions to generate (n-1):3 FT­(U)­CAs and progressively shorter-chain products.^44,45^ Unlike the β-oxidation-like reactions, which primarily explain PFCA formation while preserving the perfluoroalkyl tail, the one-carbon removal paradigm focuses on repeated shortening of the FT chain and progressive biodefluorination. More recent studies suggest that this process can proceed through cascading transformation cycles, generating a suite of downstream TPs with progressively shorter chain lengths under aerobic conditions.^40^ Similar transformations have also been reported under nitrate-reducing and methanogenic conditions for a broad spectrum of FTs, including n:2 FTCAs,^46^ FTUCAs,^42^ and n:2 fluorotelomer alcohols (FTOHs),^47,48^ albeit with substantially slower kinetics and lower degrees of defluorination, often at the Cβ position.^49,50^ However, quantitative understanding of transformation kinetics remains largely limited to aerobic systems, whereas kinetic behavior under anoxic and anaerobic conditions has received comparatively little attention, likely because of the substantially slower transformation rates.^40,47,51^ Collectively, these studies suggest that redox conditions strongly influence transformation kinetics and outcomes and point to the existence of competing enzymatic routes. A unifying mechanistic framework that resolves pathway divergence, individual reaction steps, cascade-level flux, and transformation kinetics under environmentally relevant redox regimes remains lacking for 5:3 FTCA.^52–54^

Here we investigated the biotransformation of 5:3 FTCA using six sets of microcosms inoculated with WWTP activated sludge and anaerobic sludge under four redox conditions (i.e., aerobic, anoxic nitrate-reducing, anaerobic sulfate-reducing, and anaerobic methanogenic). Target LC/MS/MS and nontarget Nano-ESI-HRMS analyses were combined to comprehensively profile PFCAs and other TPs over 18 days in aerobic microcosms and 5 months (∼150 days) in anoxic/anaerobic microcosms. The complementary analytical approaches were designed to cover both known and previously unrecognized TPs spanning a wide range of abundances and chain lengths. Compared with conventional electrospray ionization, Nano-ESI-HRMS improves ionization and ion-transfer efficiency, reduces salt-induced matrix suppression, minimizes sample consumption, and limits carryover through replaceable nanoemitters, making it particularly well suited for time-resolved analysis in PFAS biotransformation studies.^30,55–57^ Without LC separation, this Nano-ESI-HRMS approach also enables the detection of ultrashort-chain PFCAs, such as perfluoropropionic acid (PFPrA) and trifluoroacetic acid (TFA),^30^ and captures transient and/or low-abundance intermediates, such as keto-n:3 FTCA and 3-F n:3 FTCA, which would likely have been missed by conventional target analyses alone.

The comprehensive TP profiles generated in this study provide the mechanistic resolution needed to reconcile two longstanding mechanistic paradigms. Here we propose a bifurcated biotransformation framework, in which n:3 FTCAs encounter a biotransformation fork. One branch proceeds through a Cβ-centered enoyl-hydration pathway (P_E_) that resembles β-oxidation-like chemistry and preserves the perfluoroalkyl tail, terminating in PFCA formation. The second branch proceeds through a Cα-centered hydroxylation pathway (P_H_) that parallels the previously proposed one-carbon removal mechanism, driving iterative chain shortening and biodefluorination. Rather than representing mutually exclusive mechanisms, these pathways operate as competing branches of a unified biotransformation network whose relative contributions are governed by redox conditions. TPs were further allocated to P_E_ and P_H_ pathways to quantify cascade-level contributions and link TP fluxes to fluorine mass recovery (FMR) using a combination of absolute and semiquantitative approaches. Together, our results elucidate how redox conditions strongly regulate 5:3 FTCA removal rates, TP formation kinetics, pathway partitioning, biodefluorination, and the extent of fluorinated chain shortening. These findings provide new mechanistic and kinetic insight into FTCA biotransformation in wastewater treatment systems and inform strategies for steering PFAS fate away from terminal PFCA accumulation and toward progressive defluorination.

## 2 Materials and Methods

### 2.1 Chemicals and Reagents

Reagent-grade 5:3 fluorotelomer carboxylic acid (2H,2H,3H,3H-perfluorooctanoic acid, 5:3 FTCA) was obtained from two commercial suppliers (SynQuest Laboratories, Alachua, FL, USA, ≥ 97%; Arctom Scientific, Westlake Village, CA, USA, ≥98%). 5:3 FTCA analytical standard (FPePA) was purchased from Wellington Laboratories (Guelph, ON, Canada). Impurities in these 5:3 FTCA standards were examined prior to the microcosm setup (Figure S1, SI Section S2.1). For absolute and semiquantification, analytical standards of PFCAs, including perfluorobutanoic acid (PFBA), perfluoropentanoic acid (PFPeA), perfluorohexanoic acid (PFHxA), and ^13^C-labeled PFCAs were purchased from Wellington Laboratories. PFPrA (>97%) and 1,2,3-^13^C_3_-2H,2H,3H,3H-perfluorooctanoic acid were obtained from Cambridge Isotope Laboratories (Tewksbury, MA, USA). Native PFAS standards were used to prepare calibration curves for absolute- and semiquantification, while isotope-labeled standards were used as internal standards (ISs) (Table S1). Sodium fluoride (NaF, ≥99%) and total ionic-strength adjustment buffer (TISAB II) were purchased from Thermo Fisher Scientific (Waltham, MA, USA). Sodium nitrate (NaNO_3_, ≥99%), sodium sulfate (Na_2_SO_4_, ≥99%), ammonium acetate (NH_4_OAc), molecular biology grade D-(+)-glucose (≥99.5%), and potassium hydroxide (KOH) were purchased from Sigma-Aldrich (St. Louis, MO, USA). Phosphate buffered saline (PBS, 10×, pH 7.4) was purchased from Quality Biological (Gaithersburg, MD, USA). LC/MS-grade methanol and acetonitrile (Optima, ≥99.9%; Thermo Fisher Scientific) were used for sample preparation and chromatographic separation. Ultrapure water (18.2 MΩ·cm) was produced by a Milli-Q RC Synthesis system (Millipore, Bedford, MA, USA). Synthetic wastewater was prepared as previously reported^51,58^ (see details in the SI Section S1.1).

### 2.2 Microcosm Assays under Different Redox Conditions

#### 2.2.1 Anoxic/Anaerobic Assays

As biological precursor of biosolids with potential land applications, anaerobic sludge was collected from three municipal WWTP anaerobic digesters (WWTP L, R, and P; Table S2) and handled following prior work.^46^ Equal volumes (post centrifugation) of these three anaerobic sludges were combined and then washed three times with 1× PBS and centrifuged (8,500 rpm, 10 min) to remove residual chemicals. 100 μL of 40 mM 5:3 FTCA methanolic stock was preloaded to sterile serum bottles, allowing methanol (MeOH) to evaporate overnight. Then, 50 mL sulfate-free ammonium mineral salts (AMS) medium and 1 g wet anaerobic sludge were amended to yield an initial 5:3 FTCA concentration of 80 μM. This concentration was selected to match our previous microcosm assays^46,51,59^ and also used in other studies^60,61^ to provide sufficient analytical sensitivity for low-abundance TP identification and fluoride release measurement.

To establish nitrate- and sulfate-reducing microcosms, sodium nitrate or sodium sulfate was added to the sulfate-free AMS medium to achieve 20 mM NO_3_^–^ and SO_4_^2–^ at the start of incubation, respectively.^47,62^ The headspace of these bottles was flushed with N_2_ and crimp-sealed with butyl septa to create anaerobic conditions. Methanogenic microcosms were prepared using the same sulfate-free AMS medium with the headspace filled with 50:50 (v/v) CO_2_/N_2_ gas mixture.^63^ All anoxic/anaerobic microcosms were prepared in triplicate and incubated in an anaerobic chamber (BACTRON300, Airgas, PA, USA) filled with a gas mixture of 90% N_2_/5% CO_2_/5% H_2_ throughout 5 months.

Bottles were well mixed before sampling using disposable syringes with sterile 20-gauge needles. An aliquot sample was split for target LC/MS/MS, nontarget Nano-ESI-HRMS PFAS analysis, electron-acceptor measurement (NO_3_^–^/SO_4_^2–^), and free fluoride measurement after filtration with 0.22 μm polyethersulfone filters.^64^ Nitrate and sulfate were monitored by ion chromatography (IC, Section 2.6). For anoxic nitrate-reducing microcosms, NO_3_^–^ was reamended to ∼20 mM at month 4 when it dropped below 25% of the starting concentration (Figure 1b). Methane production in methanogenic headspace was monitored by GC-FID. Anaerobic sludge remaining in the serum bottles at the end of the incubation period was collected and preserved at −20 °C for future analysis.

**Figure 1:**
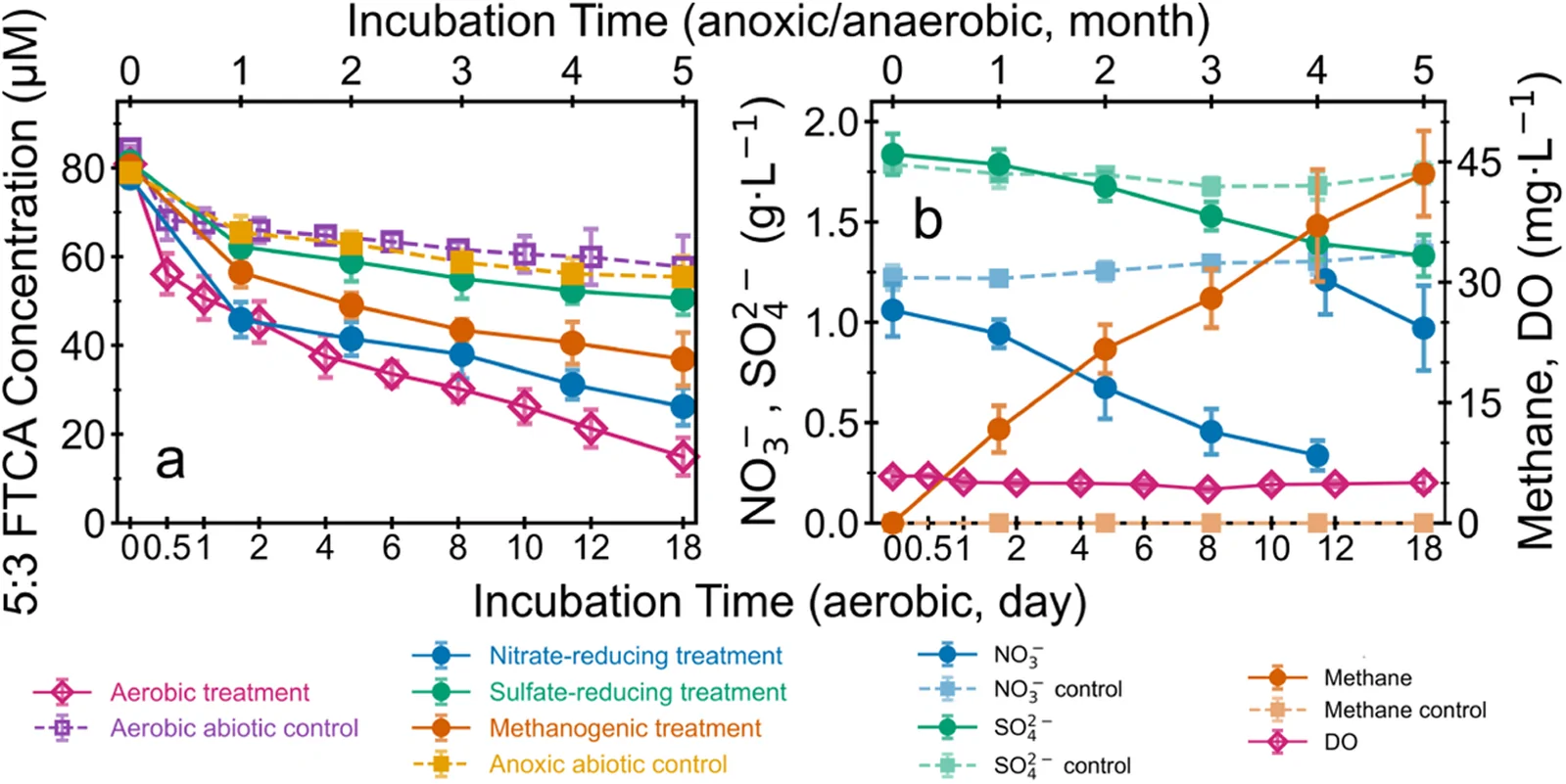
(a) Biotransformation of 5:3 FTCA in aerobic versus anoxic/anaerobic (i.e., nitrate-reducing, sulfate-reducing, and methanogenic) microcosms as compared to their corresponding abiotic controls. (b) Temporal changes in geochemical indicators confirming distinct redox conditions in aerobic (i.e., dissolved oxygen [DO]) and anoxic/anaerobic (i.e., nitrate, sulfate, and methane) microcosms. NO_3_^–^ was reamended at month 4 in the anoxic nitrate-reducing microcosms. DO concentrations in aerobic microcosms were maintained at 5.1 ± 0.5 mg·L^–1^, equivalent to 46–65% oxygen saturation relative to the atmosphere. All *y*-axes are plotted on linear scales.

#### 2.2.2 Aerobic Assays

Three activated sludge samples (R1, R2, and R3) were collected from individual aeration tanks operated in parallel at the WWTP R and processed following previous studies.^51^ Briefly, activated sludge samples were washed and inoculated to serum bottles preloaded with 5:3 FTCA to match the initial concentration of the anoxic/anaerobic assays. Synthetic wastewater (50 mL) was added to supply chemical oxygen demand (COD) and nutrients mimicking domestic sewage compositions (e.g., glucose/acetate/NH_4_^+^/phosphate with trace salts; COD ≈ 415 mg L^–1^; pH ≈ 7.3).^51^ Aerobic microcosms were incubated on an orbital shaker (130 rpm) at room temperature, and dissolved oxygen (DO) was monitored by an Ohaus DO Meter. Each of the three sludge sources was inoculated in triplicate. Although three sludge samples were collected from separate aeration tanks, they exhibited similar biotransformation patterns. Thus, results of the aerobic assays were reported using a combined sample size of n = 9.

#### 2.2.3 Control Assays

Abiotic controls were prepared identically to the live treatments, consisting of the same medium and 5:3 FTCA dose, except that mixed anaerobic sludges or activated sludges were autoclaved (121 °C, 15 psi, 60 min, Hirayama HICLAVE HV-25, Marshall Scientific) before inoculation. Microbial controls containing anaerobic sludges or activated sludges with corresponding medium but without 5:3 FTCA addition were incubated under the same conditions. Analytical controls consisted of 5:3 FTCA and the corresponding medium without sludges. Pure methanol, acetonitrile (MeCN), and DI water were used as reagent controls to track background signals from reagents and instrument noises. At select sampling intervals, samples were filtered immediately and stored at −20 °C. All analyses were performed within 72 h after collection. A summary of all treatment and control microcosms is provided in Table S3.

### 2.3 Target PFAS Analysis via LC/MS/MS

Absolute quantification of the parent 5:3 FTCA and target PFCA TPs (C4–6) was performed by LC/MS/MS under the multiple reaction monitoring (MRM) mode. Analyses used an Agilent 1290 Infinity II LC system coupled to an Agilent 6470A triple quadrupole mass spectrometer operated in negative electrospray ionization mode (ESI). A Waters Symmetry C18 analytical column (2.1 × 100 mm, 3.5 μm) was used for analyte separation. Filtered samples were diluted 50 times with LC/MS grade methanol and spiked with IS mixture before quantification. The injection volume was set to 10 μL. The mobile phase was set referring to EPA Method 1633, with phase A (2 mM ammonium acetate in DI water with 5% MeCN) and phase B (100% MeCN), and a flow rate of 0.30 to 0.35 mL/min according to the LC gradient shown in Table S4.

The triple quadrupole mass spectrometer was operated in negative ESI mode with capillary voltage at −3.5 kV, desolvation gas temperature and flow rate at 350 °C and 8 L/min, and nebulizer pressure at 25 psi. Quantifier and qualifier MRM transitions, collision energies, fragmentor voltages, and cell acceleration voltages are listed in Table S5. Calibration was established using 7 serially diluted standards. Calibration checks were performed every 30–35 injections.

### 2.4 Nontarget PFAS Analysis by Nano-ESI-HRMS

Biotransformation products were semiquantified using nanoelectrospray high-resolution mass spectrometry (Nano-ESI-HRMS) on a Q-Exactive hybrid quadrupole-Orbitrap mass spectrometer (Thermo Fisher Scientific), in accordance with our established workflow.^30,51,65,66^ Samples were analyzed in negative-ion full-scan mode (*m*/*z* 60–900) at the 140,000 resolving power (at *m*/*z* 200). Putative mass ions of interest (MIIs) were subjected to data-dependent MS/MS using quadrupole isolation (±0.2 *m*/*z*) and collision induced dissociation (CID) for structure interpretation.

Nano emitters were prepared from capillaries on a laser puller (Sutter Instruments Model P-2000, Novato, CA, USA). Samples were introduced at a consistent flow rate (1.0 μL/min) by a NE-1000 syringe pump, with a −3.0 kV spray potential applied to the needle. The capillary temperature was set to 225 °C. Data acquisition for each analytic run was initiated once the automatic gain control (AGC) indicator stabilized at 100% (with an AGC target set at 1e6), and the total ion current variation was <8% across scans. Each full-scan MS1 file was compiled in 75 scans. The evolution of product ions in response to the collision energy (CE) variation (10–55 V) during MS2 was used to identify primary fragmentation and secondary breakdown profiles.

Raw MS was exported from Xcalibur and processed with our validated pipeline to identify PFAS features distinguishable from noise via the function of spectra preprocessing.^65^ For ion/fragment annotation, mass spectra were screened following stringent criteria: <3.5 ppm for *m*/*z* 126–350, < 5 ppm for *m*/*z* 351–900, and <10 ppm for *m*/*z* 60–125. MS2 spectra were interpreted using (i) a local PFAS fragment library and (ii) manual annotation via assigning elemental compositions to product ions based on exact mass and PFAS substructures. Confidence levels (CLs) were assigned to identified PFAS TPs following established framework, with criteria for each CL described in Table S6.^67,68^ The logic for nontarget MII capturing is summarized in Section 2.5.

Nano-ESI-HRMS was also used to screen potential ultrashort-chain PFCAs, including PFPrA and TFA, as the direct infusion workflow did not require chromatographic retention, which was a limitation in conventional LC methods for highly polar small carboxylates.^69,70^ Nano-ESI can efficiently transfer charged nanodroplets to gas phase^56^ and the acquisition covered low mass ranges. The detection of ultrashort-chain PFCAs were supported by exact mass, MS2 evidence, and comparison with control sets.^30^ Further details about Nano-ESI-HRMS semiquantification (e.g., calibration and determination of LOQs) are provided in SI Section S1.2 and Tables S7–S8.

### 2.5 Nontarget Screening and Semiquantification

Nontarget features were extracted from HRMS data using a Python-based workflow previously developed for PFAS biotransformation nontarget screening, with minor modifications for single-batch experiments.^65^ MS1 spectra were denoised by screening for peaks with signal-to-noise ratios (S/N) > 3 that appeared in ≥50 of 75 scans in each analytical run. The signal of each retained feature was normalized to the IS based on a relative intensity (RI) threshold of 0.1%. Features were aligned across samples and treatments by matching their *m*/*z* values within 3 ppm mass error.

Each candidate feature in live treatments was compared against a control set (aforementioned in Section 2.2) containing abiotic controls, analytical controls, microbial controls, and reagent blanks. Features were retained when there is no matching in the control set or their RI in live treatments exceeded controls/blanks by at least 25-fold. Features indicative of potential biotransformation, showing statistically significant changes in RI over time and/or consistent increases in treatments relative to abiotic controls were collected as MIIs as putative TPs. Homologue-series relationship was built using CF_2_ based Kendrick mass defect (KMD) mapping as supporting evidence of chain shortening effects during FTCA biotransformation. Codes for Python-based nontarget workflow are available at https://github.com/Daitoueqaq/HRMS-modification.

Since many TPs did not have authentic standards, their concentrations were measured and reported as surrogate-equivalent estimates. Three calibration/native standards were used, including 5:3 FTCA for FTCA intermediates, PFPeA for C4–C6 PFCAs, and PFPrA for PFPrA and TFA (Table S7). For semiquantification, calibration curves, method detection limit (MDL) and limit of quantification (LOQ) values of calibration/native standards are applied to corresponding analyte groups (see details in SI).

### 2.6 Anionic Electron Acceptor Measurement by Ion Chromatography (IC)

Nitrate and sulfate in anoxic/anaerobic microcosms were monitored by IC to verify adequate electron acceptors were maintained. Analyses were conducted using a Dionex ICS-1500 ion chromatography system coupled with AS50 autosampler. The IC is equipped with a conductivity detector, an IonPac AS18 IC analytical column (250 × 4 mm), and an AG18 IC guard column (50 × 4 mm). Filtered samples (400 μL) were diluted by 20 times with Milli-Q water, with 80 μL KOH stock solution added for pH adjustment. The mixture was gently vortexed, equilibrated for ∼30 min, passed through 0.2 μm IC Acrodisc syringe filters (13 mm), and transferred to IC vials prior to the injection. The IC mobile phase consists of 23 mM KOH with a flow rate of 1.0 mL/min. Calibration standards were prepared in the same way as samples and were analyzed at the beginning of monthly IC measurement. Field and reagent blank signals remained below the MDLs (Table S9) for both anions.

### 2.7 Free Fluoride Measurement and FMR Calculation

Free fluoride release was quantified using an ion-selective electrode (SI Section S1.3). Filtered samples were mixed 1:1 (v/v) with TISAB II and analyzed by an Orion Dual Star meter coupled with a 9609BNWP fluoride-specific electrode. Sodium fluoride standards were freshly prepared and measured in the same matrix to build calibration curves (Figure S2) before each measurement. The practical limit of detection (LOD) for fluoride in the sludge matrices was at ∼4 μM (i.e., ∼76 μg/L) based on the calibration regression and matrix spiked recoveries. This method was adapted from with EPA SW-846 Method 9214 ^71^ and the results were validated using IC.^30^ Signals below LOD were treated as nondetect (ND), unless ≥ 2 of 3 replicates exceeded the LOD (RSD ≤ 25%, retain the mean of triplicate).

FMR was estimated as the molar sum of fluorine in (i) all abiotic-corrected organofluorines and (ii) free fluoride, normalized to the initial fluorine dose. Details of FMR algorithms, together with calibration fitting, are provided in SI Section S1.4.

### 2.8 Methane Production by GC-FID

Headspace methane was measured to track methanogenic activity. At each sampling point, 100 μL of microcosm headspace was extracted using a gastight syringe equipped with a stopcock and immediately injected to a TRACE 1300 gas chromatography (GC, Thermo Scientific) coupled with a flame ionization detector (FID). The injector and detector temperatures were set at 200 and 250 °C, respectively. Other chromatographic conditions followed our laboratory’s established protocols for short-chain alkane gas measurement.^72,73^ Methane eluted at a retention time of ∼1 min and gas-phase concentrations were calculated based on the established standard curve calibration.

## 3 Results and Discussion

### 3.1 Redox-Dependent Kinetics of 5:3 FTCA Biotransformation

Marked differences in 5:3 FTCA biotransformation kinetics were observed across redox conditions (Figure 1a). In aerobic microcosms inoculated with activated sludge (n = 9), rapid removal of 5:3 FTCA occurred under well-maintained dissolved oxygen levels (4.2–5.8 mg L^–1^). Over 18 days, 5:3 FTCA concentrations declined from 80.89 ± 1.99 to 14.94 ± 4.25 μM, corresponding to an abiotic-corrected pseudo-first-order removal rate of 0.074 d^–1^ (Figure S3).

In contrast, biotransformation proceeded much more slowly in anoxic/anaerobic microcosms inoculated with the mixed anaerobic sludge from WWTPs L, R, and P. Over five months, biological removal accounted for 36.5%, 8.0%, and 24.4% of the initial 5:3 FTCA dose under anoxic nitrate-reducing, anaerobic sulfate-reducing, and anaerobic methanogenic conditions (n = 3 per anoxic/anaerobic treatment), respectively. Estimated abiotic-corrected pseudo-first-order removal rates under nitrate-reducing (0.0045 d^–1^) and methanogenic (0.0028 d^–1^) conditions were more than an order of magnitude lower than those under aerobic conditions. Sulfate-reducing microcosms exhibited minimal net loss of 5:3 FTCA, consistent with limited sulfate reduction (∼20%; Figure 1b). In contrast, pronounced nitrate reduction and methane production confirmed active anoxic/anaerobic metabolism in nitrate-reducing and methanogenic systems, supporting their capacity for FTCA transformation.

Collectively, these results establish a redox hierarchy for 5:3 FTCA biotransformation in our experimental system (O_2_ ≫ NO_3_^–^ > CO_2_ ≫ SO_4_^2–^), a trend further corroborated by TP profiles and fluorine-based mass balances discussed below. Given its comparatively progressive anoxic/anaerobic transformation, nitrate-reducing conditions were selected for detailed comparison with aerobic systems. Across all redox conditions, comparable abiotic losses of parent 5:3 FTCA (26.7–31.6%) were observed, likely reflecting sorption, evaporation, and other processes.^74^

### 3.2 Redox-Controlled Accumulation of Chain-Shortening TPs

Coupling with 5:3 FTCA biotransformation, PFCAs and chain-shortened FT­(U)­CAs have been reported as key TPs under varying redox conditions in previous studies.^40,41,44,75^ Here, we profiled the time-resolved dynamics of these targeted analytes using the combination of LC/MS/MS and Nano-ESI-HRMS. Figure 2 exhibits the temporal TP profiles in the aerobic and anoxic nitrate-reducing microcosms as the removal of the parent 5:3 FTCA demonstrated in Figure 1a. Results for TP profiles under anaerobic sulfate-reducing and methanogenic conditions are provided in Figures S6 and S7, respectively. Identification details for the detected TPs, including formula assignment, observed *m*/*z*, mass error, CL, detection regime, first emergence and time-integrated concentrations, are summarized in Tables S10–S12. MS2 spectra of TPs are provided in Figures M1–M14. Quantitative analysis of these key TPs enables the elucidation of the divergence of biotransformation pathways during 5:3 FTCA biotransformation, as discussed later.

**Figure 2:**
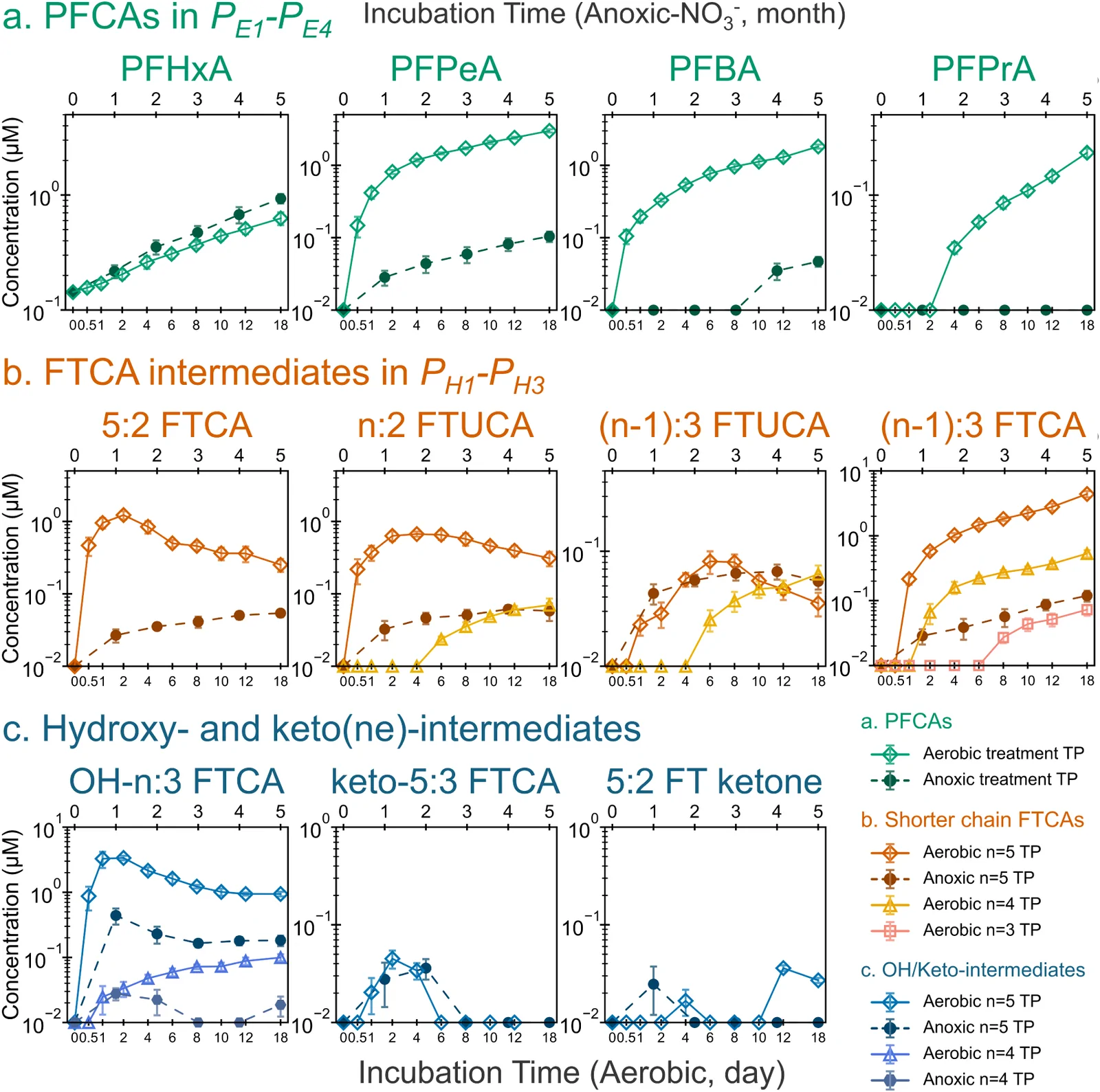
Temporal concentration profiles of 5:3 FTCA-derived TPs under aerobic versus anoxic (i.e., nitrate-reducing) conditions based on the combination of absolute and semiquantitative measurements. Major TPs are categorized into (a) PFCAs, (b) chain-shortened FTCA intermediates (n:2 FTCA, n:2 FTUCA, (n-1):3 FTUCA, and (n-1):3 FTCA), (c) hydroxyl- and keto-intermediates and FT ketone. For OH-n:3 FTCA, n:2 FTUCA, (n-1):3 FTUCA, and (n-1):3 FTCA, homologues of different chain lengths are distinguished by color shading as indicated in the legends. Only top *x*-axes are plotted on linear scales, while all *y*-axes are plotted on log scales. Individual aerobic and anoxic TP profiles with abiotic control are provided in Figures S4 and S5, respectively.

#### 3.2.1 Terminal PFCAs

Under aerobic conditions, gradual accumulation of C3–C6 PFCAs was observed (Figures 2a and S4). By day 18, PFHxA, PFPeA, PFBA, and PFPrA reached abiotic-corrected concentrations of 0.42 ± 0.08, 2.99 ± 0.19, 1.82 ± 0.14, and 0.23 ± 0.02 μM, respectively, collectively accounting for 13.5% of biologically removed 5:3 FTCA. While C4–C6 PFCAs appeared rapidly, PFPrA emerged only after day 4, and TFA was detected sparsely after day 8. The estimated TFA level was near its LOQ and accounted for <0.1% of the initial 5:3 FTCA dose, even under the high parent concentration used, suggesting that biotransformation of long-chain FTCAs and other polyfluorinated precursors is unlikely to be a dominant source of environmental TFA.^76^

Under nitrate-reducing conditions, PFCA formation was more restricted. Only C4–C6 PFCAs accumulated gradually over five months, reaching 0.73 ± 0.11 μM PFHxA, 0.10 ± 0.02 μM PFPeA, and 0.04 ± 0.01 μM PFBA. PFBA was detected only after four months, and no further chain-shortened products (PFPrA or TFA) were observed.

These contrasting PFCA profiles underscore strong redox control over terminal PFCA formation in regard to product distribution, reaction kinetics, and chain-shortening extent. Aerobic conditions favored sustained shortening toward PFPrA (and potentially TFA), with PFPeA and PFBA as the dominant sink. In contrast, anoxic nitrate-reducing conditions favored accumulation of PFHxA, a nonchain-shortened product, at levels approximately 2-fold higher than those observed aerobically at the end when normalized to parent consumption. Although PFPeA and PFBA were formed anoxically/anaerobically, their delayed appearance and lower abundance point to limited cascading and defluorination potential, a pattern further supported by other TPs discussed below.

#### 3.2.2 Chain-Shortened FT­(U)­CAs

In parallel to PFCA generation, shorter chain FTCAs and FTUCAs were also major TPs detected in both aerobic and anoxic/anaerobic conditions (Figure 2b). 5:2 FTCA and 5:2 FTUCA appeared immediately along with the initiation of 5:3 FTCA biodegradation under aerobic conditions. 5:2 FTCA and 5:2 FTUCA peaked between day 2 and 4, and then gradually declined to 0.25 ± 0.06 and 0.31 ± 0.09 μM to day 18, respectively, indicating efficient further transformation to downstream TPs. Notably, the concentration ratios of these intermediates relative to 5:3 FTCA as the parent compound approached near-constant values during the later incubation stage, suggesting kinetically coupled turnover consistent with quasi-steady-state behavior (see detailed discussion in SI Section S2.4, Figure S9). In contrast, accumulation of these two TPs was slower and more gradual in anoxic nitrate-reducing conditions.

Subsequent formation of 4:3 FTUCA and 4:3 FTCA followed the appearance of 5:2 species. In aerobic microcosms, 4:3 FTUCA emerged on day 1, peaked on day 6, and declined thereafter, while 4:3 FTCA accumulated continuously. Both TPs appeared under nitrate-reducing conditions after one month. Additional downstream FT­(U)­CAs, including 4:2 FTUCA, 3:3 FTUCA, 3:3 FTCA, and 2:3 FTCA, were detected exclusively under aerobic conditions, with delayed occurrence. 4:2 FTCA was observed sporadically at low signal intensity but supported by accurate mass matching, CF_2_-based KMD analysis, and CID fragmentation (Figure M9). None of these deeper cascade products were detected under nitrate-reducing conditions, highlighting limited progression beyond early shortening steps.

### 3.3 Detection of Transient Hydroxylated and Ketone Intermediates

Hydroxylated and ketone intermediates have been proposed as transient TPs in FTCA and related PFAS precursor biotransformation pathways,^40,41,52,77^ yet direct evidence remains limited, particularly for ketone intermediates.^77^ Using Nano-ESI-HRMS, we captured several transient intermediates (Figure 2c), including the first report of keto-5:3 FTCA (CL 3a; Table S6) and 5:2 FT ketone (CL 2b; Table S6). Although positional isomers of hydroxylated and keto- intermediates could not be resolved by the current approach, their temporal behavior provides critical mechanistic insight.

#### 3.3.1 Hydroxyl-n:3 FTCAs

OH-5:3 FTCA appeared rapidly following initiation of 5:3 FTCA degradation and declined thereafter, exhibiting transient behaviors under both redox conditions (Figure 2c). Peak concentrations reached 3.36 ± 0.32 μM (day 2) aerobically and 0.44 ± 0.15 μM (month 1) under nitrate-reducing conditions. These trends closely mirrored those of 5:3 FTUCA, particularly under aerobic conditions. Similarly, coupling with the occurrence of 4:3 FT­(U)­CA, OH-4:3 FTCA was detected at lower levels, gradually accumulating to 0.10 ± 0.01 μM by day 18 aerobically.

#### 3.3.2 Keto-n:3 FTCAs and n:2 FT Ketones

Keto-5:3 FTCA appeared as a low-abundance and early intermediate, detectable between day 1 and 4 aerobically and between month 1 and 2 anoxically. Detection was supported by both MS1 and MS2 evidence (Figure M10). Keto-4:3 FTCA was also observed from MS1 aerobically around day 10.

FT ketones were intermittently detected. Appearance of 5:2 FT ketone (MS1 and MS2 supported by Figures M11 and M12) followed keto-5:3 FTCA under both aerobic and anoxic conditions. Low abundance of 4:2 FT ketone (CL 3d; Table S6) was also observed aerobically (Figure M14). The strong electron-withdrawing effect of perfluoroalkyl chains stabilizes enolate anions, facilitating their detection by Nano-ESI-HRMS and unlocking their mechanistic significance.^78^

### 3.4 A Bifurcated Framework for n:3 FTCA Biotransformation

Based on our integrated TP evidence, we propose a bifurcated mechanistic framework for 5:3 FTCA biotransformation (Figures 3 and 4), which reconciles the major mechanistic paradigms previously proposed for FT transformation. Previous studies have described FTCA biotransformation using β-oxidation-like^18,40^ and “one-carbon removal”^44^ mechanisms. Rather than representing mutually exclusive mechanisms, our results indicate that these paradigms describe complementary branches of a common biotransformation network. In this unified framework, n:3 FTCAs encounter a biotransformation fork that directs flux into two competing pathways: a Cβ-centered enoyl-hydration pathway (P_E_) and a Cα-centered hydroxylation pathway (P_H_). The former functions largely as a redox-insensitive conduit to PFCA accumulation, whereas the latter, which is favored under aerobic conditions, acts as a conveyor for iterative chain shortening of the perfluoroalkyl tail and biodefluorination.

**Figure 3:**
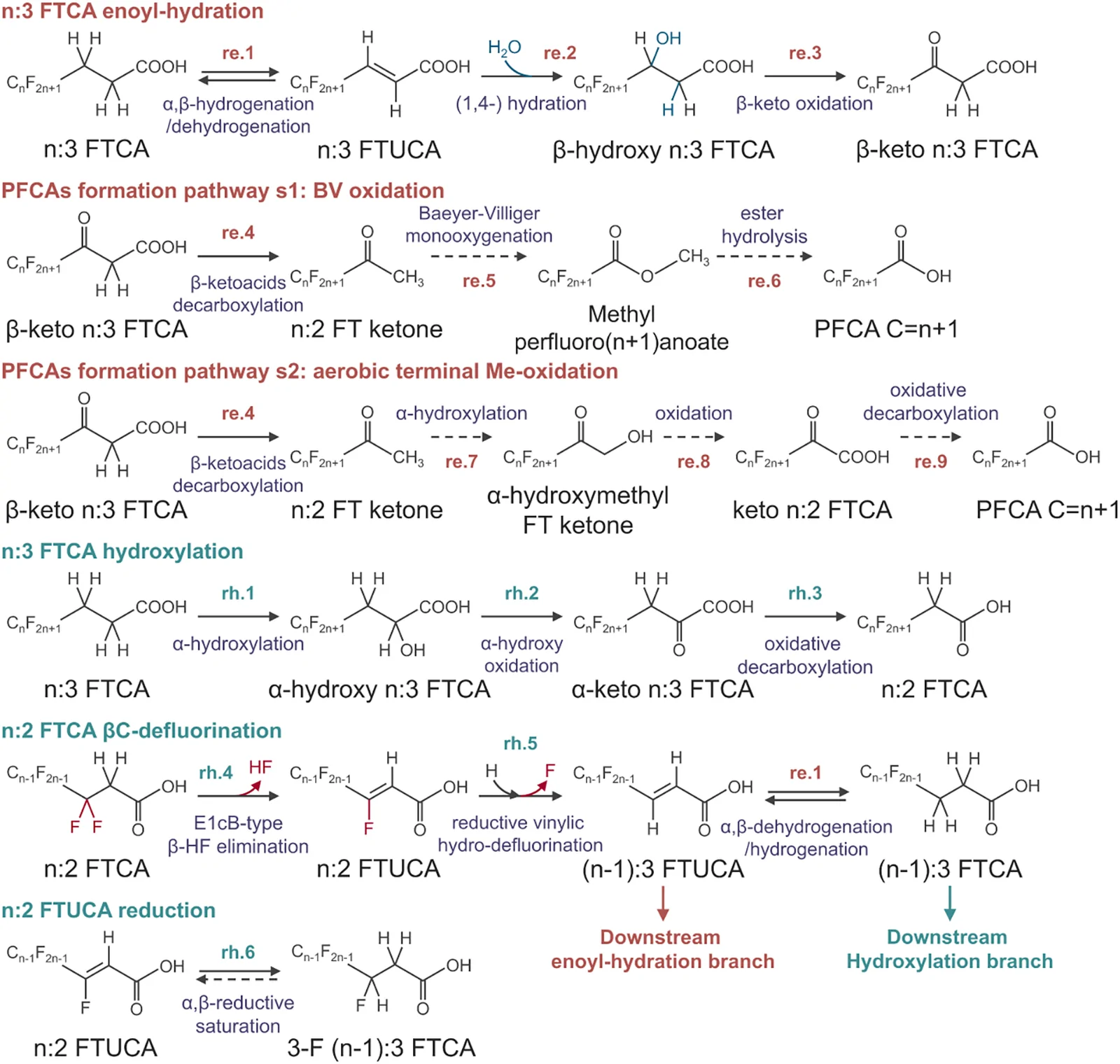
Schematic of bifurcated n:3 FTCA biotransformation reactions associated with the enoyl-hydration pathway (P_E_) versus the hydroxylation pathway (P_H_), which are highlighted in brick red and cyan teal, respectively. Solid arrows indicate proposed pathway steps supported by detected TPs in this study and/or by established fluorotelomer PFAS biotransformation chemistry. Dashed arrows indicate inferred steps for which the specific reaction intermediate or reaction sequence was not directly confirmed in this study. Newly detected keto-n:3 FTCA, n:2 FT ketone, and 3-F n:3 FTCA support proposed transformation mechanisms.

**Figure 4:**
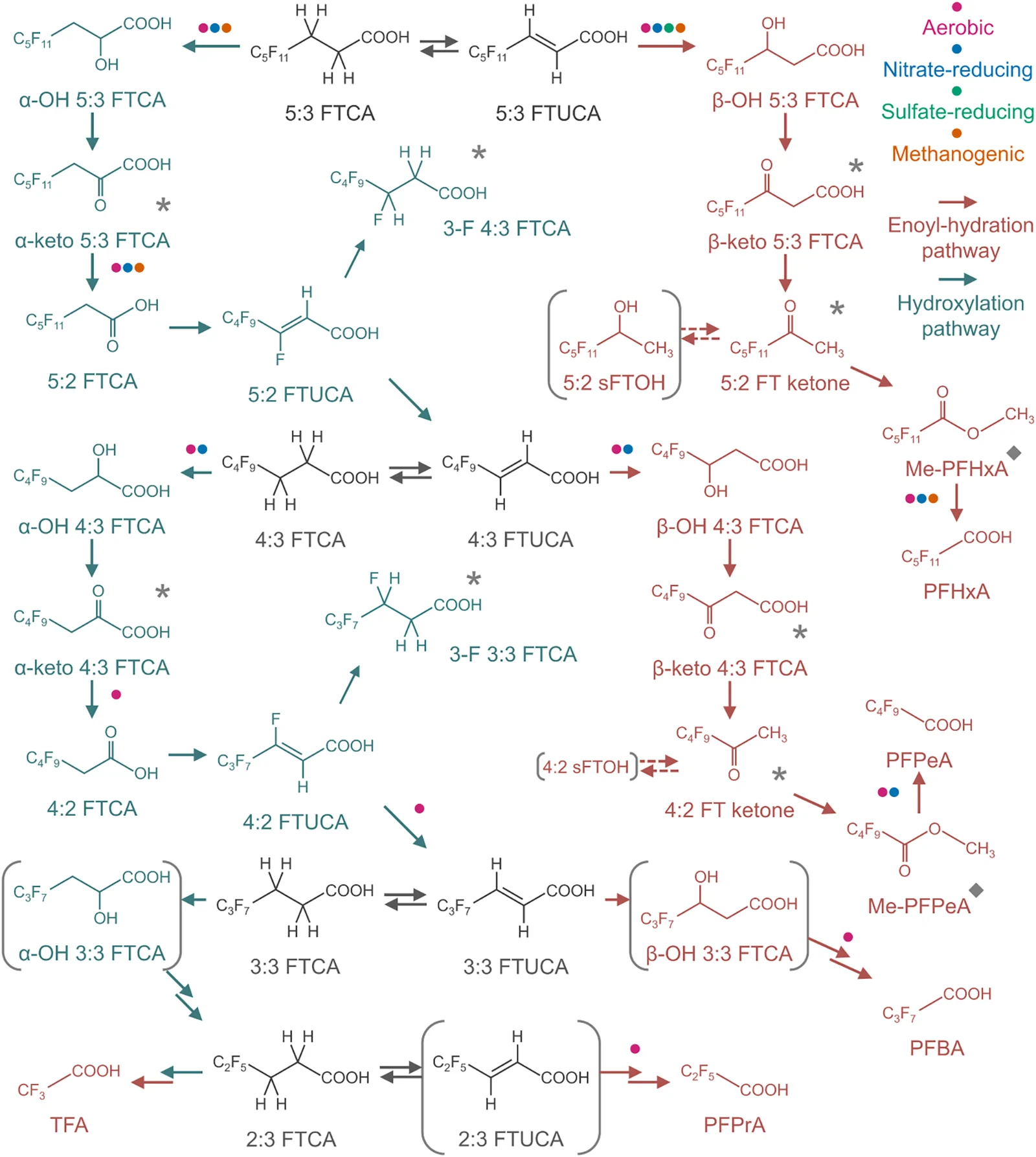
Proposed biotransformation pathways of 5:3 FTCA under aerobic and anoxic/anaerobic conditions. As the starting points of each biotransformation bifurcation, n:3 FT­(U)­CAs (*n* = 2–5) are colored in gray. TPs associated with the enoyl-hydration and hydroxylation pathways are shown in brick red and cyan teal, respectively. TPs enclosed in brackets denote nondetected species. All remaining TPs were identified at CL 3 or higher, with keto-4:3 FTCAs, 4:2 FTCA, 4:2 FT ketone, 3-F 4:3 FTCA, and 2:3 FTCA identified as CL 3d. Asterisks denote newly identified TPs, intermediates, and/or isomers. The α- and β-OH/keto structures indicate their proposed positional assignments. Staggered paired arrows denote multistep conversions. Dashed arrows indicate literature-supported transformations that were not directly confirmed in this study. Diamond marks proposed methyl perfluoroalkyl ester intermediates in the BV-type route that were not directly detected.

#### 3.4.1 The Enoyl-Hydration Pathway (P_E_)

The enoyl-hydration pathway (Figure 3) starts with the reversible dehydrogenation (re.1) of saturated n:3 FTCA to its unsaturated enoyl form (n:3 FTUCA).^42,44,79^ This step is analogous to the α, β-dehydrogenation and back-reduction catalyzed by acyl-CoA dehydrogenase (ACAD)-type enzymes, using electron transfer flavoproteins to introduce Cα-Cβ double bond (EC 1.3.8.-).^36^ Dehydrogenation initiates the enoyl-hydration pathway and can proceed under both aerobic and anoxic/anaerobic conditions.

Formed enoyl (n:3 FTUCA) undergoes a Michael-type hydration (re.2) catalyzed by enoyl hydratase-type enzymes (EC 4.2.1.-). This adds water across the double bond, and installs an −OH group at Cβ and a proton at Cα to yield β-hydroxy product.^80^ The strongly electron-withdrawing perfluoroalkyl tail further increases the electrophilicity at Cβ, favoring the 1,4-addition of water to produce β-hydroxy n:3 FTCA.^81,82^ Then, the Cβ-hydroxyl group can undergo dehydrogenation (re.3), analogous to the reaction catalyzed by dehydrogenase (EC 1.1.1.35).^36^ This removes the stereocenter at Cβ and generates β-keto n:3 FTCA.

β-keto n:3 FTCA tends to decarboxylate (re.4) through a cyclic six-membered transition state that generates an enol intermediate and then tautomerizes to n:2 FT ketone.^83–85^ The decarboxylation is mechanistically favored by β-carbonyl and catalyzed by decarboxylase (EC 4.1.1.-). In our scheme (Figure 3), the sequence of enoyl hydration, dehydrogenation, and decarboxylation to a fluorotelomer ketone constitutes the core of the enoyl-hydration pathway under both aerobic and anoxic/anaerobic conditions.

Along this enoyl-hydration pathway, we propose two possible mechanisms for PFCA formation. In the route s1, the decarboxylated n:2 FT ketone intermediate proceeds via Baeyer–Villiger (BV)-type monooxygenation (re.5) catalyzed by BV monooxygenase (BVMO; EC 1.14.13.-).^86^ This involves the insertion of an oxygen atom favorably between the carbonyl and the methyl groups facilitated by the electron-withdrawing perfluoroalkyl tail.^87^ Generated methyl perfluoroester can undergo esterase hydrolysis (re.6) to produce PFCAs. This proposed PFCA formation via BV oxidation is mechanistically plausible but has not yet been proven.

An alternative terminus methyl α-oxidation route s2 has been previously proposed by Washington (2015)^41^ and another study.^88^ The route initiates with the monooxygenase-catalyzed hydroxylation (re.7) at the terminal methyl of the FT ketone to generate an α-hydroxymethyl FT ketone, which is subsequently oxidized (re.8) to α-keto n:2 FTCA. The intermediate can then proceed with oxidative decarboxylation (re.9) to generate the corresponding PFCA.

Because n:3 FTCAs are structural analogues of fatty acids, β-oxidation chemistry is mechanistically relevant to their biotransformation. Classical β-oxidation requires activation of the substrate to a CoA thioester prior to the sequential dehydrogenation, hydration, oxidation, and carbon–carbon bond cleavage reactions. However, no FTCA-CoA conjugates were detected in this study. We therefore use the enoyl-hydration pathway to describe the β-oxidation-like reaction sequence supported by the observed TPs, while recognizing that the involvement of CoA-mediated activation remained unresolved.^40,89^

#### 3.4.2 The Hydroxylation Pathway (P_H_)

The hydroxylation pathway (P_H_) (Figure 3) initiates the cascades of the perfluoroalkyl chain shortening from n:3 FTCA. The proposed entry step is monooxygenase-catalyzed hydroxylation (rh.1) at Cα to form α–OH n:3 FTCA (EC 1.14.-.-). Direct electrophilic oxidation at Cβ is less favored as the strongly electron withdrawing CF group reduces electron density at Cβ.^90^ This α-hydroxylation initiated process is well studied,^40,41,44^ with the proposed route involved in oxidative decarboxylation (rh.2; rh.3) of the α-hydroxy n:3 FTCA to form n:2 FT aldehyde, followed by rapid oxidation to n:2 FTCA. However, n:2 FT aldehyde was neither detected in our microcosms nor was convincingly measurable in other previous studies.^91,92^ Thus, here we adopt minor updates based on our detection of keto-5:3 FTCA. We propose that α–OH n:3 FTCA is first dehydrogenated to α-keto n:3 FTCA by dehydrogenase (EC 1.1.1.-), and then the keto-acid intermediate undergoes oxidative decarboxylation (EC 4.1.1.- & EC 1.2.1.-) to produce n:2 FTCA. This step is plausible since C–C bond cleavage by decarboxylases typically act on keto-acids rather than directly on secondary alcohols.^93,94^

The generated n:2 FTCA will proceed dehydrofluorination across the Cα–H/Cβ–F via an E1cB-type elimination (rh.4) and form n:2 FTUCA. The Cα proton is acidified by carbonyl and Cβ-F, and favors deprotonation to form a resonance-stabilized carbanion.^95^ The anionic state intermediate can drive E1cB-type β-elimination of fluoride at Cβ. The poor leaving fluoride at Cβ disfavors E1 or E2 elimination mechanisms. The generated n:2 FTUCA undergoes reductive β-defluorination (rh.5) through a F/H replacement at Cβ. This mechanism has been reported in recent studies about flavin-based electron bifurcating of enoyl-fluoride.^42,49,50,96^

At this critical junction point, the generated (n-1):3 FTUCA can re-enter the enoyl-hydration pathway (P_E_) mentioned above via hydration to produce β–OH (n-1):3 FTCA. Alternatively, it can be reduced via α,β-hydrogenation to (n-1):3 FTCA. The saturated substrate becomes available to initiate a subsequent hydroxylation pathway (P_H_) to generate α–OH (n-1):3 FTCA.

Together, these two pathways form a cycling system in which (n-1):3 FT (U)­CAs repeatedly re-enter the bifurcation point, enabling cascading chain shortening or termination depending on redox conditions (Figure 4). This framework reconciles the diverse TP profiles observed here and across prior studies.^40,44,45^

### 3.5 Redox-Specific Partitioning between Competing Pathways

Quantitative pathway apportionment, performed by assigning quantified TPs to P_E_ and P_H_ pathways following allocation schematics and pathway/cascade assignment described in SI Section S1.5, Figure S10, and Tables S13–S14, revealed stark redox-dependent differences in pathway routing (Figure 5b). Under aerobic conditions, ∼95% of explainable TPs were routed through P_H_ at 2 days, sustaining deep cascading and generating a diverse TP pool throughout the incubation. Downstream TPs are featured by shorter-chain PFCAs via P_E2–4_ and FT­(U)­CAs via P_H2/3_, constituting to 8.34–11.93 μM as total TPs after 1 day (Figure 5a). Their detection was supported by CF_2_-based KMD analysis (Figure 5c and Table S10).

**Figure 5:**
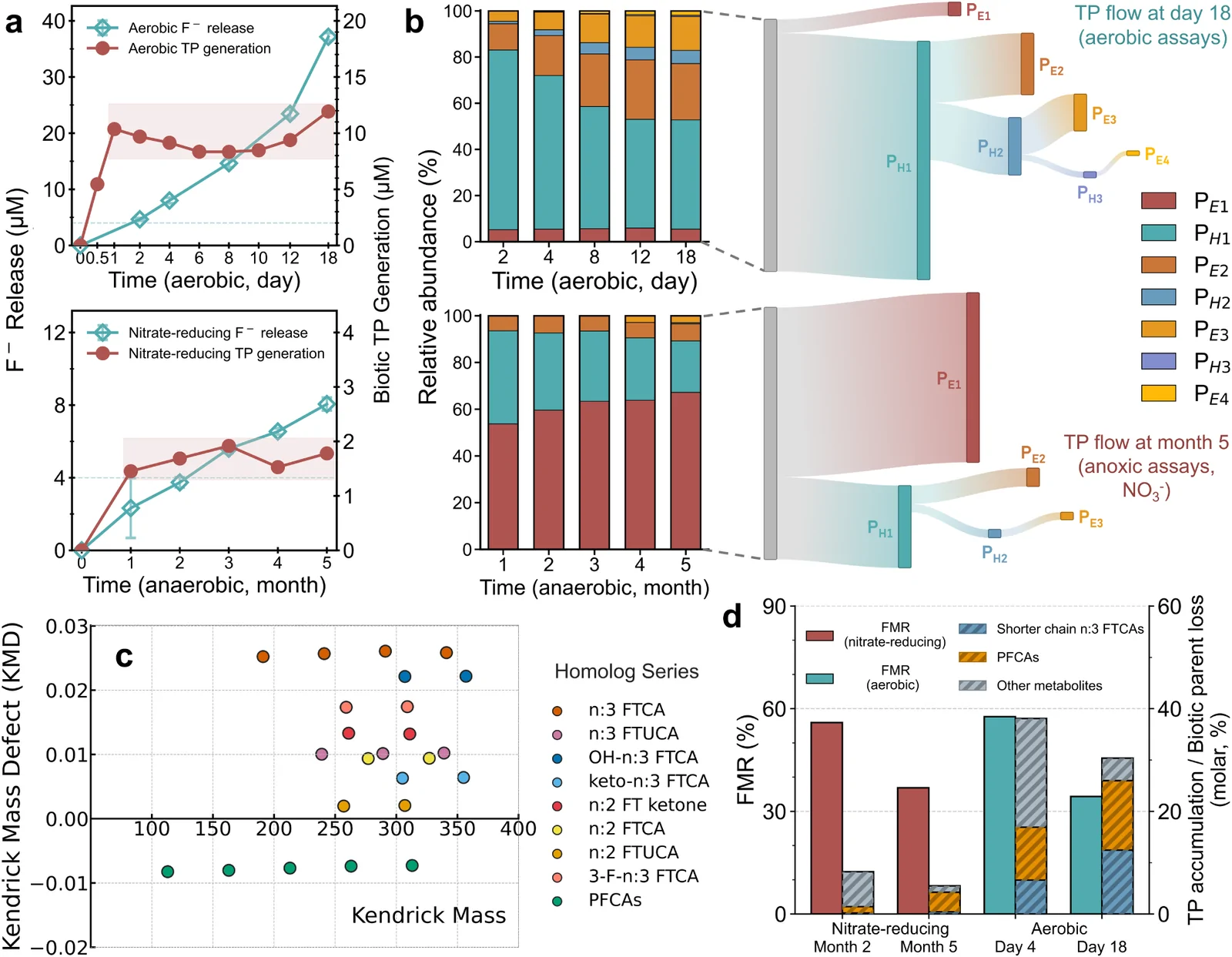
Overview of fluoride release and TP dynamics during bifurcated FTCA biotransformation. (a) Generation of free fluoride and total TPs during aerobic (18-day) and anoxic nitrate-reducing (5-month) incubations, with data corrected for the corresponding abiotic controls. (b) Percent distributions of TP cascades generated from bifurcated FTCA biotransformation via the enoyl-hydration (P_E_) and hydroxylation (P_H_) pathways over the incubation period, and their allocation across cascade levels (1–4) at the end of the incubation. (c) KMD plot illustrating homologous series of TPs with varying perfluoroalkyl chain lengths. (d) FMR and percent contributions of major TPs at select incubation times.

In contrast, anoxic nitrate-reducing microcosms funneled >60% of explainable TPs into the initial P_E_ with stagnant downstream progression, while P_H_ operated as a slow and secondary process, evident by trace accumulation of shorter-chain products, such as PFPeA in P_E2_ and 4:3 FTCA in P_H1_. The total explainable TP pool under anoxic nitrate-reducing conditions remains constant at ∼1.8 μM (Figure 5a), with PFHxA (∼0.91% of initial parent dose) as the largest product sink.

This redox hierarchy likely reflects differences in microbial energy metabolism and redox cycling under the tested electron-acceptor conditions. Aerobic respiration supported the deeper cascade progression and the greater extent of biodefluorination, whereas nitrate reduction sustained slower transformation that was dominated by PFHxA formation. These observations suggest that the enoyl-hydration pathway remains operative under both aerobic and anoxic conditions but proceeds at a relatively lower overall rate under anoxic conditions. In contrast, the hydroxylation pathway is strongly favored under aerobic conditions and is largely responsible for the enhanced cascade progression and biodefluorination.

Free fluoride release (Figure 5a) mirrored this divergence. Aerobic microcosms accumulated 37.16 ± 0.82 μM fluoride by day 18, approximately 4-fold higher than anoxic nitrate-reducing systems achieved over five months. Fluoride release equivalents from quantified TPs explained ∼72.8% (aerobic, 18 day) and ∼9.9% (anoxic nitrate-reducing, 5 month) of the observed fluoride release. The strong agreement under aerobic conditions supports consistency between TP quantification and fluoride measurements, whereas the substantially lower recovery under anoxic nitrate-reducing conditions points to unresolved fluorine sinks.

Fluorine mass recovery (FMR) peaked during early incubation phase (Figure 5d) as high as 55.9% under anoxic nitrate-reducing conditions at month 2 and 57.7% under aerobic conditions on day 4. However, FMR declined below 40% by the end of both incubations, suggesting involvement of pathways beyond the scope of current analyses. Several recent studies have highlighted conjugation with low-molecular-weight thiols, including CoA, glutathione, and mycothiol, as important reactions for 5:3 FTCA and related downstream products.^39,65,97^ In addition, incorporation of FTCAs and other partially fluorinated alkyl compounds into cellular membranes or other hydrophobic biomass fractions may further contribute to the observed fluorine mass imbalance.^74^ These processes likely represent parallel sinks that operate alongside the bifurcated pathways described here and warrant targeted investigation in future studies.

## 4 Environmental Implications: Steering the Biotransformation Fork

5:3 FTCA is a prevalent PFAS precursor in wastewater, landfill leachates, and related matrices, making its environmental fate highly relevant. This study demonstrates that redox conditions act as a regulator at the fork of FTCA biotransformation, determining whether flux is shunted toward PFCA accumulation or carried forward through cascading chain shortening and defluorination. Oxygen availability, in particular, governs whether n:3 FTCAs are trapped in a terminal sink or conveyed through sequential shortening steps.

Mechanistically, identification of ketone intermediates refines longstanding pathway hypotheses and strengthens the conceptual foundation of FTCA biotransformation. The bifurcated framework presented here offers a unifying lens for disparate TP patterns interpretation and provides a basis for designing biological treatment strategies that favor defluorination over PFCA formation.

Several reactions proposed in this bifurcated framework, as well as in previous anoxic/anaerobic studies,^98–100^ are classically associated with monooxygenase or oxygenase activity, raising questions about their operation under anoxic/anaerobic conditions. It is important to note, however, that these reactions were substantially attenuated rather than absent in nitrate-reducing microcosms, consistent with limited oxygen availability. In complex sludge and biosolid matrices, trace oxygen can arise from intracellular redox processes, endogenous reactive oxygen species, or micro-oxic niches, supporting low-flux oxygenase activity.^101–104^ In addition, previous studies have also reported that oxygen-independent hydroxylation can occur in anaerobic microorganisms.^105–107^ Furthermore, several steps in the proposed pathways may proceed via oxygen-independent mechanisms, including dehydrogenation-decarboxylation sequences or water-derived oxygen insertion, particularly for keto-acid intermediates.^108,109^ Therefore, the proposed framework reflects conserved reaction logic rather than strict enzyme identity, with redox conditions governing pathway flux, kinetics, and cascade depth rather than binary on/off behavior.

At the same time, substantial fluorine mass gaps highlight important limitations. Undetected conjugates, biomass-associated fluorine, and low-abundance intermediates remain poorly constrained, underscoring the need for integrated metabolomics, isotopic tracing, and biomass-associated fluorine analyses. Although experimental toxicity evaluation was beyond the scope of this study, *in silico* toxicity predictions (detailed in SI Section S1.6 and S2.6, Figure S11, Table S15) indicated that the major TPs formed along both pathways generally exhibited comparable (or even lower in many cases) predicted toxicities than 5:3 FTCA parent, consistent with previous report suggesting that chain shortening of PFAS does not necessarily increase toxicological concern.^34^ Addressing these remaining analytical and toxicological uncertainties will be essential for translating mechanistic insight into effective PFAS mitigation strategies.

## Supplementary Material





## Notes

Detailed methods for media preparation, fluoride measurement, nontarget analysis, and *in silico* toxicity estimation, as well as additional results on 5:3 FTCA biodegradation kinetics, low-abundance intermediates, and toxicity comparisons between 5:3 FTCA and its transformation products (PDF)
Mass spectra for important transformation products detected in this study (PDF)
