# Supplementary material for: Bifurcated Fluorotelomer Carboxylic Acid Biotransformation: Enoyl-Hydration versus Hydroxylation Regulated by Redox Conditions
Source: Environ Sci Technol. 2026 Sep 22;60(39):27917–30. doi: 10.1021/acs.est.6c04089 (PMC13648231; doi:10.1021/acs.est.6c04089)
Supplement: Supplementary file 1 [file es6c04089_si_001.pdf]

Supporting Information

for

Bifurcated Fluorotelomer Carboxylic Acid Biotransformation:

Enoyl-hydration versus Hydroxylation Regulated by Redox Conditions

Boyuan Su[1], Hao Chen[1], John Washington[2,3], Mengyan Li[1,*]

[1] Department of Chemistry and Environmental Science, New Jersey Institute of Technology, Newark, New Jersey, United States

[2] Department of Geology, University of Georgia, Athens, GA, USA

[3] USEPA, Office of Research and Development, Center for Environmental Measurement and Modeling Retired, Athens, GA, USA

*Address correspondence to Dr. Mengyan Li

ORCID ID: 0000-0002-7664-4836

Email: mengyan.li@njit.edu

Phone: +1-973-642-7095

Fax: +1-973-596-3586

Table of Contents

S1. Materials and Methods……………………………..…………………………………......3

S2. Results and Discussion……………………………………………………………..12

S3. Supporting Figures and Tables………………………………………………….....16

S1. Materials and Methods

S1.1 Media and Buffer Formulations

Synthetic wastewater

Synthetic wastewater used for the aerobic microcosms was prepared as previously reported [1-3]. The formulation per liter of Milli-Q water was: 206.4 mg D-glucose ($C_6H_{12}O_6$), 256.0 mg sodium acetate trihydrate ($CH_3COONa \cdot 3H_2O$), 35.4 mg ammonium sulfate ($(NH_4)_2SO_4$), 11.1 mg dipotassium hydrogen phosphate (K_2HPO_4), 14.2 mg calcium chloride ($CaCl_2$), 0.21 mg magnesium sulfate heptahydrate ($MgSO_4 \cdot 7H_2O$), 0.225 mg ferric chloride hexahydrate ($FeCl_3 \cdot 6H_2O$), 0.018 mg manganese(II) chloride tetrahydrate ($MnCl_2 \cdot 4H_2O$), 0.023 mg boric acid (H_3BO_3), 0.018 mg zinc sulfate heptahydrate ($ZnSO_4 \cdot 7H_2O$), 0.005 mg copper(II) sulfate pentahydrate ($CuSO_4 \cdot 5H_2O$), 1.5 mg ethylenediaminetetraacetic acid ($C_{10}H_{16}N_2O_8$), and 0.027 mg potassium iodide (KI). The formulation was used as a controlled medium that simulates the organic carbon, nutrient, and trace element composition of municipal wastewater. The synthetic wastewater had a near-neutral pH (~7.3), a chemical oxygen demand (COD) of ~415 mg L^{-1}, an ammonium-nitrogen concentration of ~7.5 mg·L^{-1}, and phosphorus concentration of ~2.0 mg P L^{-1}. The defined medium was formulated to provide adequate carbon, nutrients, trace elements, and buffering capacity to sustain microbial activity while minimizing variability associated with the composition of real wastewater. Although the ammonium and phosphorus concentrations were lower than those typically reported for municipal wastewater influents [4, 5], they remained within the range commonly used for laboratory-scale activated sludge microcosm studies.

Phosphate-buffered saline

Phosphate-buffered saline (PBS, 1×) used for biosolid washing was prepared from stock solution, diluted with Milli-Q water. 10× PBS stock solution formulation was: 90000 mg

L^{-1} sodium chloride (NaCl), 1440 mg L^{-1} potassium chloride (KCl), 7950 mg L^{-1} sodium phosphate dibasic anhydrous (Na_2HPO_4).

Sulfate-free ammonium mineral salts medium

Sulfate-free ammonium mineral salts (AMS) medium used for anoxic/anaerobic microcosms was prepared from stock solutions. The medium contained following salts per liter: 2.26 g dipotassium hydrogen phosphate (K_2HPO_4), 0.94 g monopotassium phosphate (KH_2PO_4), 15 mg calcium chloride dihydrate ($CaCl_2 \cdot 2H_2O$), 0.015 mg boric acid (H_3BO_3), 10 mg nickel(II) chloride hexahydrate ($NiCl_2 \cdot 6H_2O$), 0.25 mg ethylenediaminetetraacetic acid (EDTA), 0.05 mg cobalt(II) chloride hexahydrate ($CoCl_2 \cdot 6H_2O$), 0.005 mg copper(II) chloride dihydrate ($CuCl_2 \cdot 2H_2O$), 5 mg ethylenediaminetetraacetic acid iron(III) sodium salt, and 2 mg sodium molybdate dihydrate ($Na_2MoO_4 \cdot 2H_2O$).

S1.2 Nano-ESI-HRMS Semi-quantification and Ultrashort PFCA Detection

Nano-ESI-HRMS was used for semi-quantitative profiling of 5:3 FTCA-derived TPs, including FTCA intermediates and short/ultrashort PFCAs. The direct infusion Nano-ESI-HRMS workflow did not require chromatographic retention and acquired full-scan MS1 spectra across *m/z* 60-900, allowing ultrashort PFCAs such as TFA (CF_3COO^-, *m/z* 112.9850) and PFPrA ($C_2F_5COO^-$, *m/z* 162.9819) to be monitored with other TPs. All feature assignments were supported by exact mass (MS1), MS2 evidence (when intensity was sufficient), and comparison against abiotic, analytical, microbial, and reagent controls.

For semi-quantification, three calibration/native standards were used, including 5:3 FTCA for FTCA intermediates, PFPeA for C4-C6 PFCAs, and PFPrA for PFPrA and TFA (Table S7). Calibration/native standards were prepared at 0.05, 0.1, 0.5, 1, 10, 50, and 100 ppb. For each calibration standard, 0.05 ppb standard was injected seven times for low-level

response variability to estimate method detection limit (MDL) values.

The limit of quantification (LOQ) was calculated as

$$LOQ = \frac{10 \times \sigma}{S} \quad \text{(Eq. S1)}$$

where σ is the standard deviation of the seven low-level standard responses and S is the calibration slope.

The instrumental MDL was calculated as

$$MDL = \frac{t_{6,99\%} \times \sigma}{S} \quad \text{(Eq. S2)}$$

where t is 3.143 for seven measurements (6 degrees of freedom, 99% confidence). We reported LOQ, MDL, and corresponding original concentrations before 50× dilution in Table S8. When the calculated LOQ was lower than the lowest calibration level (i.e., 0.05 ppb), 0.05 ppb was used as reporting LOQ.

S1.3 ISE Calibration for Fluoride Measurement

Sodium fluoride (NaF) standards were freshly prepared in Millipore 18.2 MΩ·cm water and mixed with 1:1 (v/v) with total ionic strength adjustment buffer (TISAB II) right before measurement. The fluorine calibration (Figure S2) was built with ion-selective electrode (ISE) potential (mV) against the logarithm of fluoride concentration. The limit of detection (LOD) was determined based on (i) the standard deviation of triplicate readings at the two lowest calibration points divided by the calibration slope, and (ii) verified via spike-recovery level in sludge microcosm.

S1.4 Fluorine Mass Recovery (FMR)

FMR was computed according to previous research [6, 7] to evaluate fluorine balance. FMR is defined as the ratio of the total measurable fluorine (summing organofluorine (OF)

species and free fluoride) to the initial fluorine dose.

The molar sum of measurable fluorine at time t is:

$$F(t) = \sum_{i \in OF} \left(C_{i,t} \times n_{F,i}\right) + C_{F^-,t} \quad \text{(Eq. S3)}$$

where $C_{i,t}$ is the concentration of each identifiable OF, and $n_{F,i}$ is the number of fluorine atoms in that species.

The FMR was computed relative to the analytical control to account non-biological loss:

$$FMR(t) = \frac{F_{live}(t) + \left[F_{analy.}(t) - F_{ab.}(t)\right]}{F_{analy.}(t)} \quad \text{(Eq. S4)}$$

$F_{live}(t)$, $F_{ab.}(t)$, and $F_{analy.}(t)$ represent the measurable fluorine in the live treatment, abiotic sludge control, and analytical control at sampling time t, respectively.

S1.5 Time-integrated TP Abundance

To compare the overall contributions of individual TPs throughout the incubation, we calculated the area under the abiotic-corrected concentration-time curve (AUC) for each quantified TP. The AUC represents the time-integrated concentration of a TP and therefore incorporates both its abundance and persistence over the monitoring period. Unlike concentrations measured at individual sampling times, the AUC provides a cumulative metric of TP occurrence throughout the incubation.

For each TP j and sampling time t_k, the abiotic-corrected concentration is:

$$C_j^*(t_k) = C_j^{live}(t_k) - C_j^{Ab}(t_k) \quad \text{(Eq. S5)}$$

Negative values after correction are set to zero.

Abiotic-corrected concentrations $C_j^*(t_k)$ for TP j at time t were then integrated by the trapezoidal rule:

$$AUC_j([t_a, t_b]) = \int_{t_a}^{t_b} C_j^*(t)\,dt, \quad \mu M \cdot time \quad \text{(Eq. S6)}$$

where $t_a$and $t_b$denote the beginning and end of the integration interval.

For each TP, AUCs were computed over two representative monitoring windows for aerobic (0-2 d and 0-18 d) and anoxic nitrate-reducing (0-2 m and 0-5 m) (Table S12). Comparison of early- and full-incubation AUCs provides a scalar summary of how strongly each TP contributes to the observed TP pool over a certain monitoring interval. Transient intermediates are expected to exhibit relatively large AUCs during the early monitoring window but proportionally smaller contributions over the full incubation because of rapid turnover. In contrast, persistent or terminal products, such as PFCAs, accumulate throughout the incubation and therefore exhibit substantially larger full-window AUCs.

S1.5 Allocation of Composite OH- and Keto-FTCA Isomers to Biotransformation Pathways

Interval changes

For each sampling interval $[t_k, t_{k+1}]$, the change in concentration is:

$$\Delta C_j^*(k) = C_j^*(t_{k+1}) - C_j^*(t_k) \quad \text{(Eq. S7)}$$

Allocation of TPs to Biotransformation Pathways and Cascades

The allocation follows the mechanistic bifurcation of 5:3 FTCA biotransformation, including enoyl-hydration (P_E) and hydroxylation (P_H) branches. Pathway-specific TPs are

shown in Table S13. Low abundance species, including qualitative TPs occur with ≤ 3d CLs or those below LOQ, were excluded from allocation calculation.

Allocation of composite OH-/keto-FTCA pools

Since the detected pools of α/β-OH/keto isomers cannot be distinguished between P_E and P_H pathways at the same cascade level, we used a recursive branching logic to allocate these shared nodes based on the magnitude and variance of branch-specific TP sets.

The composite OH/keto-5:3 FTCA are split between the first enoyl-hydration branch (P_{E1}) and the first hydroxylation branch (P_{H1}). Since P_{H1} feeds all downstream hydroxylation and enoyl-hydration modules, its signal includes the sum of the P_{H1} TPs and all downstream branches (P_{H2}, P_{H3}, P_{E2}, P_{E3}, P_{E4}). Similarly, quantifiable OH-4:3 FTCA connects the secondary enoyl-hydration branch (P_{E2}) and an aggregate of second hydroxylation branch consisting of P_{H2} TPs and downstream derivatives (P_{H3}, P_{E3}, P_{E4}).

Notation

For pathway-specific TP (j) set S at sampling time t_k:

$$C_S(k) = \sum_{j \in S} C_j^*(t_k) \quad \text{(Eq. S8)}$$

$$\Delta C_S(k) = \sum_{j \in S} \left| C_j^*(t_{k+1}) - C_j^*(t_k) \right| \quad \text{(Eq. S9)}$$

where $C_S(k)$ is the current sum of branch-specific TPs, and $\Delta C_S(k)$ is the forward change over the next interval.

We built a two-signal weighting system for each TP set combining current concentration and flux (forward change).

$$\widetilde{\omega_S}(k;\lambda,\theta,\delta) = \lambda[C_S(k)+\varepsilon]^\theta + (1-\lambda)[\Delta C_S(k) + \delta\Delta C_S(k+1) + \varepsilon]^\theta \quad (\text{Eq. S10})$$

Weighing factor $\lambda \in [0,1]$ controls the balance between current TP set size and further flux, with values are given in Table S14. Nonlinear scaling factor θ is a modest exponent that slightly accentuates differences between branches ($\theta = 1.25$). $\varepsilon = 0.01$ is a small positive constant to avoid zero values. $\delta \in \{0,1\}$ is set as 0 when the timepoint is the second last or the last of biotransformation assay.

At the 5:3 layer, the unresolved pool allocation of OH-/keto-5:3 FTCA is derived by comparing signal from the P_{E1} branch against the cumulative signal of the P_{H1} aggregate.

$$A_{P_{E1}}(k) = \frac{\widetilde{\omega_{P_{E1}}}(k)}{\widetilde{\omega_{P_{E1}}}(k) + \widetilde{\omega_{P_{H1\_total}}}(k)}, \quad A_{P_{H1}}(k) = 1 - A_{P_{E1}}(k) \quad (\text{Eq. S11})$$

Let $Q_{iso}(k) = \sum_{j \in isomers} C_j^*(t_k)$ be the total concentration of the isomer (OH- or keto-5:3 FTCA) pool at t_k. The allocated amounts are:

$$\hat{Q}_{iso \to P_{E1}}(k) = A_{P_{E1}}(k) \cdot Q_{iso}(k) \quad (\text{Eq. S12})$$

$$\hat{Q}_{iso \to P_{H1}}(k) = A_{P_{H1}}(k) \cdot Q_{iso}(k) \quad (\text{Eq. S13})$$

A similar logic applies to the (n-1) layer (i.e., OH-4:3 FTCA). The branching is between P_{E1} and downstream P_{H2} aggregate.

$$A_{P_{E2}}(k) = \frac{\widetilde{\omega_{P_{E2}}}(k)}{\widetilde{\omega_{P_{E2}}}(k) + \widetilde{\omega_{P_{H2\_total}}}(k)}, \quad A_{P_{H2}}(k) = 1 - A_{P_{E2}}(k) \quad (\text{Eq. S14})$$

$$\hat{Q}_{iso \to P_{E2}}(k) = A_{P_{E2}}(k) \cdot Q_{iso}(k), \quad \hat{Q}_{iso \to P_{H2}}(k) = A_{P_{H2}}(k) \cdot Q_{iso}(k) \quad (\text{Eq. S15})$$

Further allocation from downstream families is not needed since no additional unresolved OH/keto pools were detected.

Sankey Flows

The per-parent allocation fraction for pathway P_i at time t_k is:

$$f_{P_i}(k) = \frac{\hat{Q}_{total \to Pi}(k)}{\Delta C_{parent}^{cum\_consumed}(k) + \varepsilon} \quad \text{(Eq. S16)}$$

where $\hat{Q}_{total \to Pi}(k)$ is the sum of measured and allocated concentrations of TPs. We used $f_{Pi}(k)$ at each sampling point to plot the Sankey flows diagram in Figure 5b, indicating how explainable parent is distributed between enoyl hydration and hydroxylation branches under aerobic and anoxic/anaerobic conditions.

S1.6. Screening-level *In Silico* Toxicity Estimation

The removal of 5:3 FTCA parent does not necessarily indicate detoxification, and TPs may retain, decrease, or increase toxicological concern. Therefore, selected accumulating and mechanistically relevant TPs/intermediates were compared with parent 5:3 FTCA using available screening-level *in silico* predictions by ADMETlab 3.0 machine learning model [8].

The selected compounds, presenting major TP classes observed in 5:3 FTCA biotransformation, included accumulating PFCAs that were assigned to the P_E, accumulating shorter-chain n:3 FTCAs assigned to the P_H, FTCA/FTUCA intermediates associated with P_H, and hydroxy-/keto-FTCA isomeric intermediates. The main toxicity endpoints used for comparison were IGC50, LC50DM, LC50FM, AMES toxicity, human hepatotoxicity, carcinogenicity, and eye corrosion. IGC50, LC50DM, and LC50FM are concentration-derived aquatic toxicity endpoints (for aquatic organisms: *Tetrahymena pyriformis*, *Daphnia magna*, and *Pimephales promelas*, respectively) reported by ADMETlab as -$\log_{10}$ of the predicted effect concentrations (i.e. higher values indicate lower predicted effect concentrations and greater predicted aquatic toxicity). AMES toxicity, human hepatotoxicity, carcinogenicity, and

eye corrosion are probability-based classification outputs, where higher values indicate greater predicted probability of the corresponding endpoint. AMES toxicity was used as a mutagenicity-related screening based on the bacterial reverse-mutation assay. Carcinogenicity provides a long-term mammalian hazard indicator. Human hepatotoxicity indicates the predicted potential for liver toxicity.

Radar plots were generated to compare each TP with parent 5:3 FTCA. Grey polygons in each subplot represent parent 5:3 FTCA, while colored polygons represent individual TPs. Since IGC50, LC50DM, and LC50FM are not probability values and have a different numerical range, these aquatic toxicity endpoints were scaled by dividing the predicted effect concentrations by 6 for visualization.

S2. Results and Discussion

S2.1. Evaluation of 5:3 FTCA Impurity

We evaluated three 5:3 FTCA source materials, including (i) SynQuest Laboratories (product 2121-3-60; stated purity 97%), (ii) Arctom Scientific (product BD-A1224178; stated purity 98%), and (iii) a methanolic standard from Wellington Laboratories (named as FPePA in the catalog) to track stock-derived impurities. Each stock was diluted to 1 mg/L 5:3 FTCA in 80:20 (v/v) methanol:water and prepared in triplicate. Samples were analyzed by Nano-ESI-HRMS using the same instrumental and MS acquisition conditions described in Section 2.4. Figure S1 shows the intensities of 5:3 FTUCA and PFHxA as impurity species normalized to the parent signal. For all time-series analyses (ΔTP, FMR, branch allocation, and mass balance), signals attribute to these impurity species were first corrected using the corresponding abiotic controls according to equation Eq. S5.

S2.2 5:3 FTCA Biodegradation Kinetics

The biodegradation kinetics of 5:3 FTCA were evaluated under aerobic and anoxic nitrate-reducing conditions using both zero-order and first-order decay models. To isolate the biological contribution, 5:3 FTCA concentrations were corrected for abiotic losses according to:

$$C_{abiotic-correct}(t) = C_{live}(t) \times \frac{C_{abiotic}(t=0)}{C_{abiotic}(t)} \quad \text{(Eq. S17)}$$

As shown in Figure S3, the pseudo-first-order kinetic model provided a better fit for 5:3 FTCA removal under both aerobic and nitrate-reducing conditions, as indicated by the higher R^2 values compared to the zero-order model. This result suggests that 5:3 FTCA degradation rates were more consistent with parent compound concentration-dependent

behavior within the examined range. The apparent first-order decay constant under aerobic conditions was 0.074 d^{-1}. In contrast, the decay constant under nitrate-reducing conditions was 0.134 m^{-1}, equivalent to 0.0045 d^{-1}, representing more than one order-of-magnitude slower transformation rate. These results are consistent with the markedly reduced biotransformation flux and cascade depth observed under anoxic/anaerobic conditions in the main text.

S2.3 Concentration Profile of 5:3 FTUCA

5:3 FTUCA appeared a rapid and transient increase in aerobic microcosms, culminating to an abiotic-corrected concentration of ~4.53 μM peaked at 24 h and then dropped gradually through day 18, where concentrations were no longer above corresponding abiotic controls after day 12. Similar but slowed pattern was also observed in nitrate-reducing microcosms, where abiotic-corrected 5:3 FTUCA concentration rose more slowly to ~1.02 μM by month 3 and decreased through the end of incubation. This initial turnover supports 5:3 FTUCA as an early intermediate that could be further transformed.

S2.4 Evidence for Kinetically Coupled Turnover of Chain-Shortening Intermediates

When normalized to abiotic-corrected 5:3 FTCA concentrations, three chain-shortening intermediate products exhibited near-constant intermediate/parent ratios during the later stage of aerobic incubation, consistent with quasi-steady-state behavior (Figure S9) [9]. The 5:2 FTCA/5:3 FTCA ratio plateaued from day 6 to day 18 at 0.010-0.012, while the 5:2 FTUCA/5:3 FTCA ratio remained within 0.013-0.015 from day 4 to day 18, despite a slight upward drift suggests modest relative accumulation of 5:2 FTUCA. The 4:3 FTUCA/5:3 FTCA ratio also showed a narrower late-stage plateau from day 10 to day 18 at 0.0015-0.0016. These patterns support kinetically coupled turnover of downstream intermediates, and are compatible with a secular-equilibrium-like relationship after the ingrowth period.

S2.5 Detection of Low-abundance Intermediates

Additional low-abundance intermediates assigned at Confidence Level 3d (Table S6) were supported by (1) exact mass match, (2) CF_2-based KMD homolog screening, and (3) evidence from identified homolog, though MS2 confirmation was limited or absent due to low signal intensity. 3-F 4:3 FTCA (*m/z* 308.9977; signal-to-noise ratio observed at 6.44-34.78; CF2-based homolog with 3-F 3:3 FTCA at *m/z* 259.0008; Figure M13) was observed in aerobic samples. Additionally, 5:2 FT secondary alcohol (5:2 sFTOH) is a plausible downstream intermediate in the 5:3 FTCA biotransformation, possibly generated through the reduction of 5:2 FT ketone. However, we were not able to detect 5:2 sFTOH above LOQ, probably due to the measuring limitations and low abundance. These species occurred sporadically and thus only provided qualitative evidence for the proposed biotransformation pathways.

S2.6 *In Silico* Toxicity Comparison between 5:3 FTCA and Its TPs

The *in silico* results (Figure S11 and Table S15) suggest that 5:3 FTCA biotransformation process is a mixed toxicity-shifting process rather than a confirmed detoxification process that produces a significant net toxicological benefit. Accumulating shorter-chain n:3 FTCAs generally showed lower predicted aquatic toxicity (IGC50, LC50DM, and LC50FM) and slightly lower carcinogenicity than the parent 5:3 FTCA, suggesting partial reduction of predicted toxicity along the P_H pathway. The accumulating PFCAs exhibited mixed behavior. PFHxA showed higher predicted aquatic toxicity than the parent, while shorter PFCAs, such as PFBA and PFPrA, showed lower predicted aquatic toxicity but retained or increased AMES toxicity and human hepatotoxicity. The FTCA/FTUCA intermediates generally retained aquatic toxicity and reduced carcinogenicity but had higher human hepatotoxicity probabilities. Hydroxy- and keto-FTCA intermediates were strongly isomer-dependent, with β-OH/keto 5:3 intermediates generally showed lower aquatic toxicity, AMES

toxicity, and carcinogenicity. α-Substituted intermediates showed higher predicted aquatic toxicity.

These predictions are *in silico* screening-level estimates rather than experimental toxicity measurements. Direct product-specific toxicity based on TP profile testing would be required to determine net detoxification.

S3. Supporting Figures and Tables

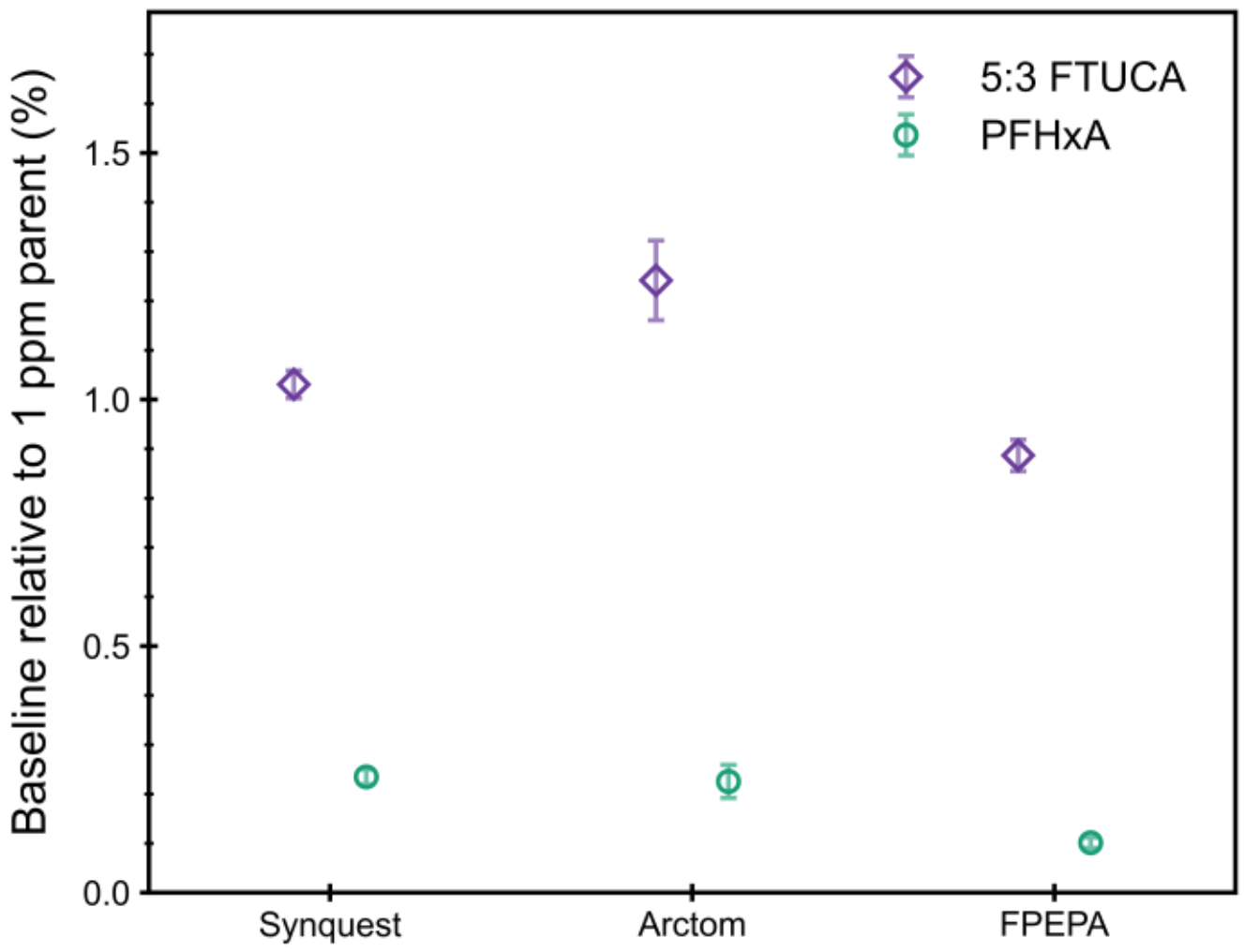

Figure S1. Intensity percentage of baseline species (5:3 FTUCA and PFHxA) relative to ~1 ppm parent 5:3 FTCA in commercial standards.

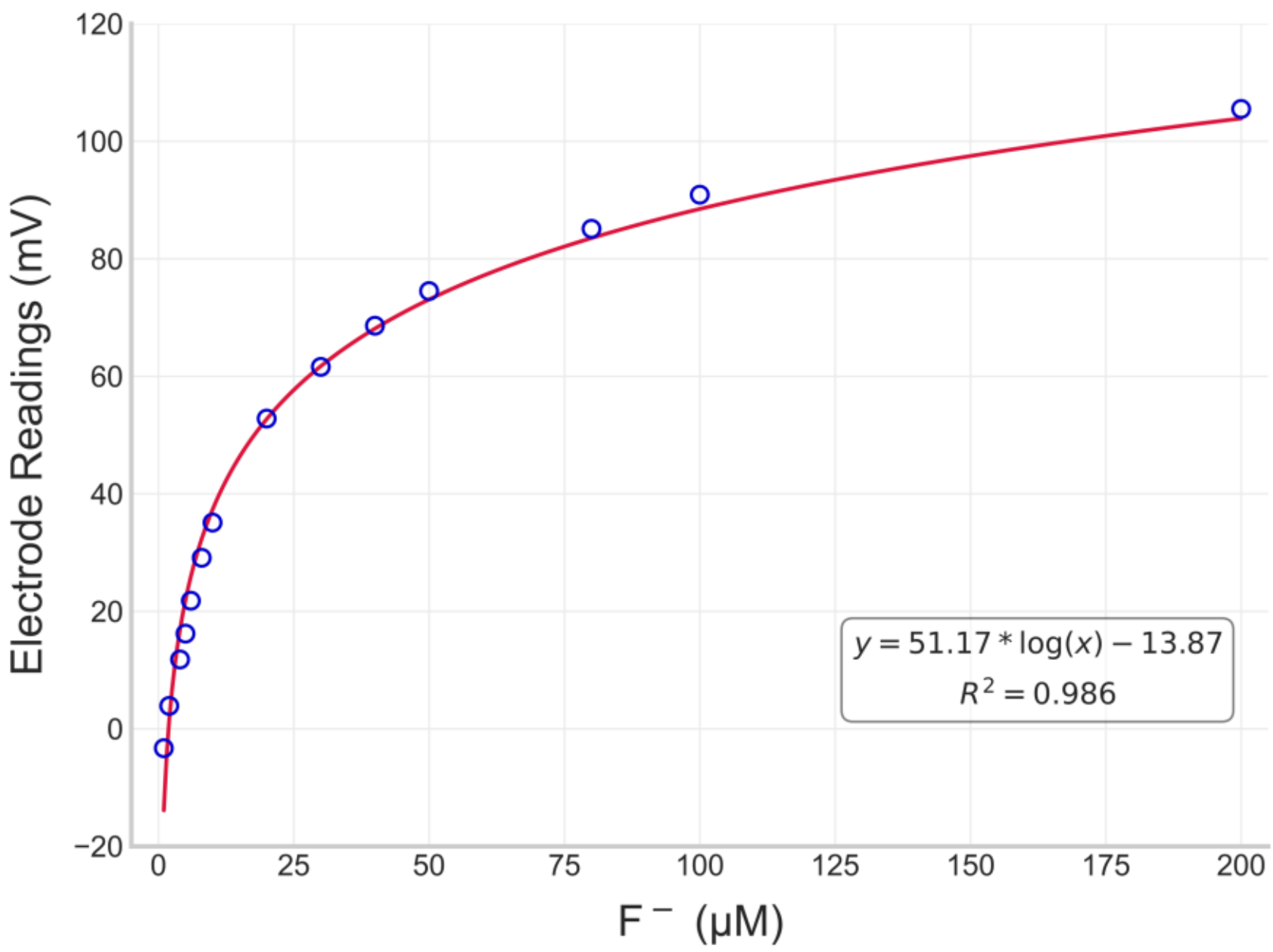

Figure S2. F^- calibration curve.

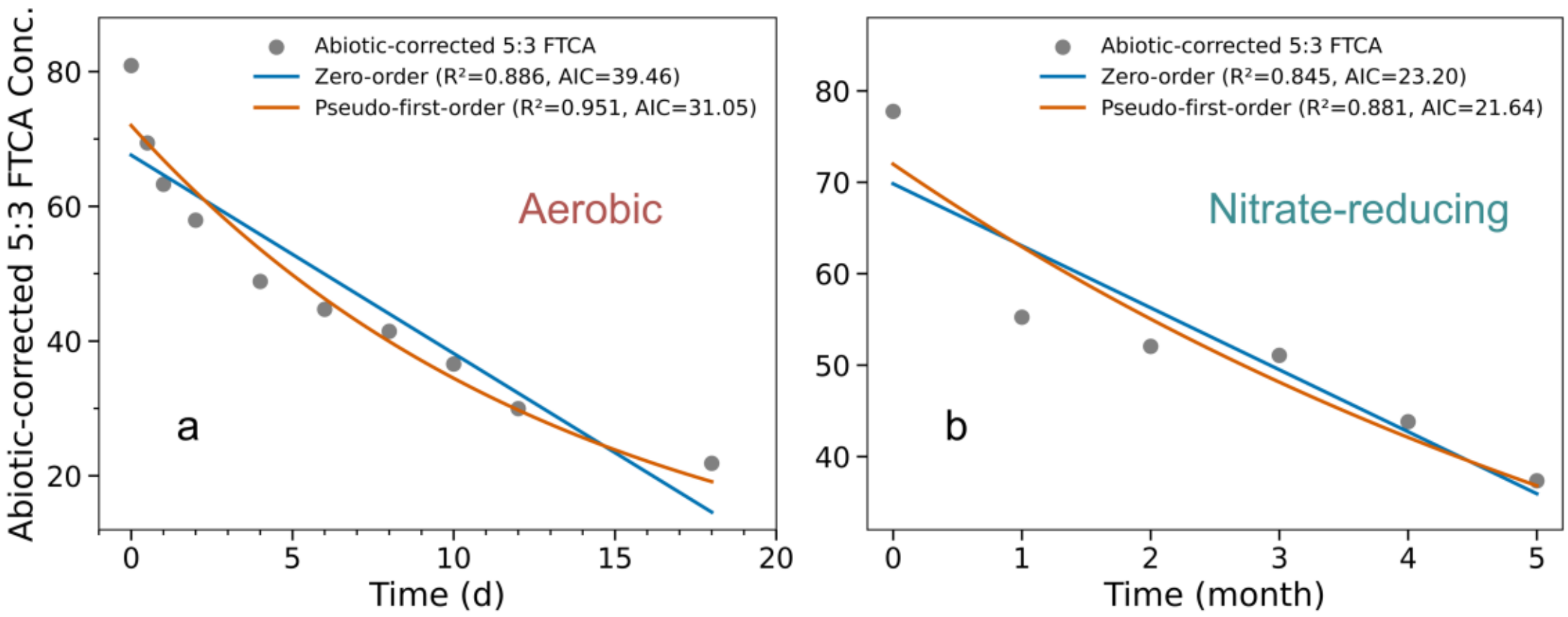

Figure S3. Empirical kinetic models fitted with abiotic-corrected 5:3 FTCA concentrations in (a) aerobic and (b) nitrate-reducing microcosms.

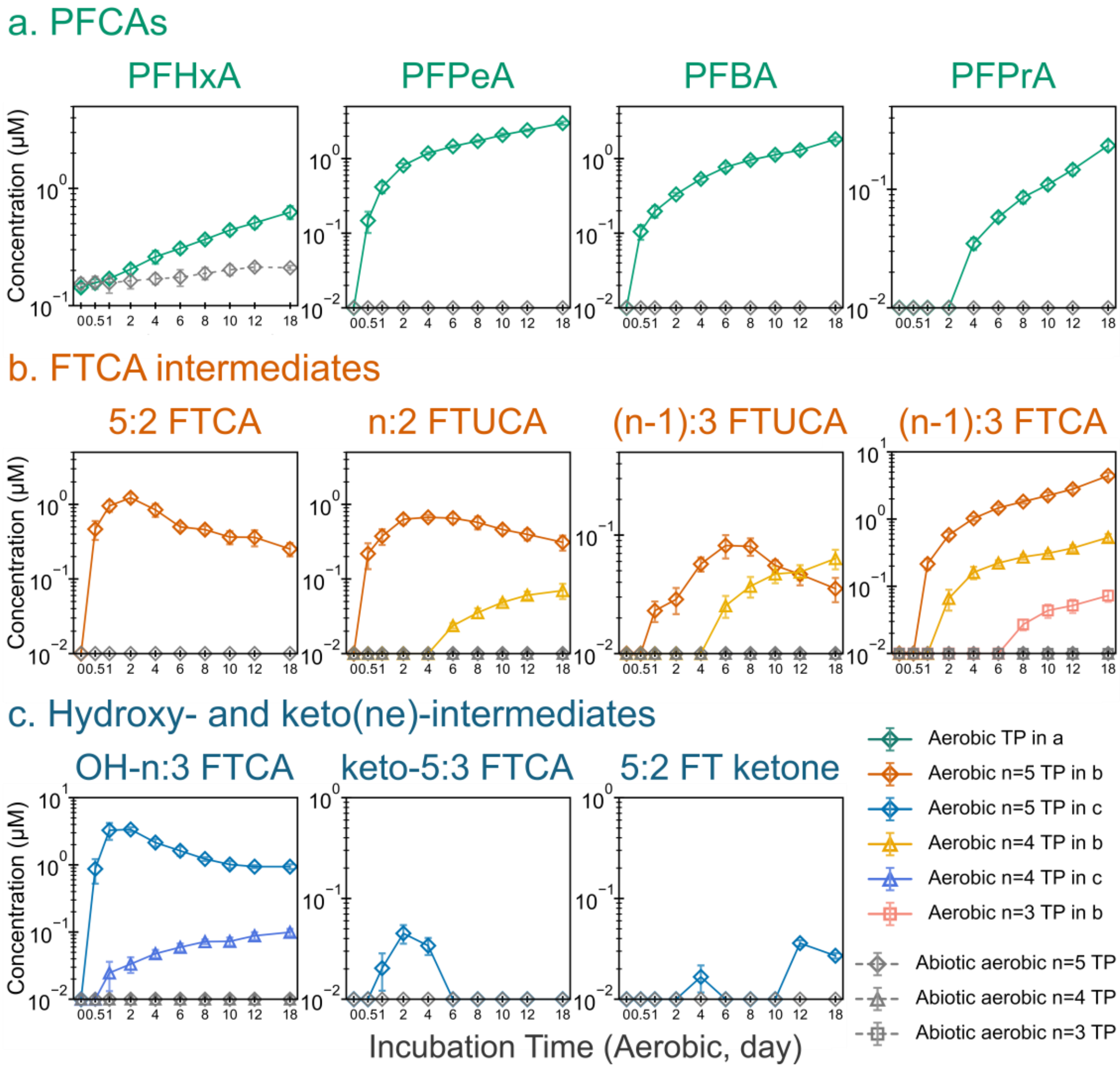

Figure S4. Temporal concentration profiles of 5:3 FTCA-derived TPs under aerobic conditions based on the combination of absolute and semi-quantitative measurements. Treatment results in this figure are also presented in Figure 2.

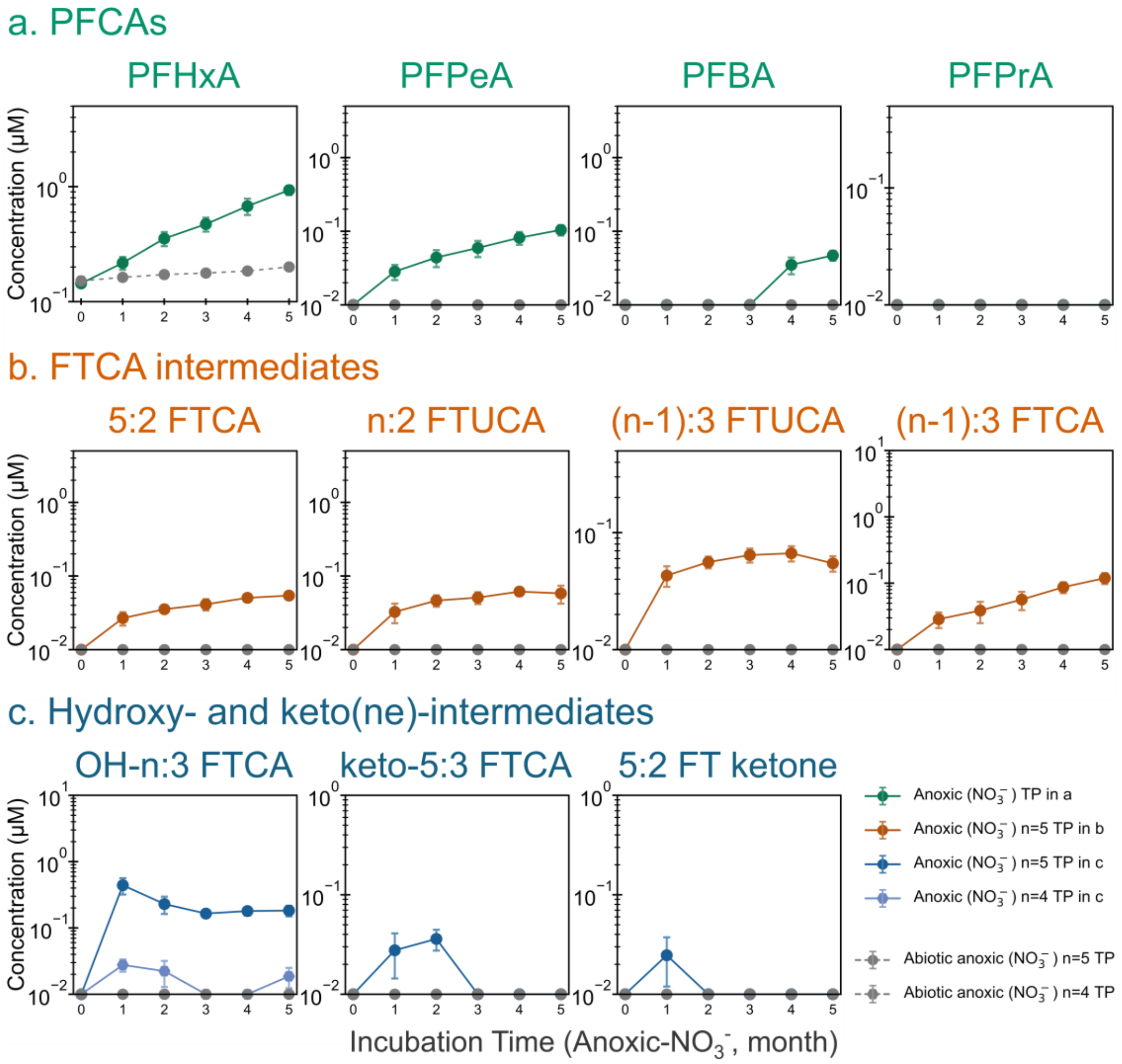

Figure S5. Temporal concentration profiles of 5:3 FTCA-derived TPs under anoxic nitrate-reducing conditions based on the combination of absolute and semi-quantitative measurements. Treatment results in this figure are also presented in Figure 2.

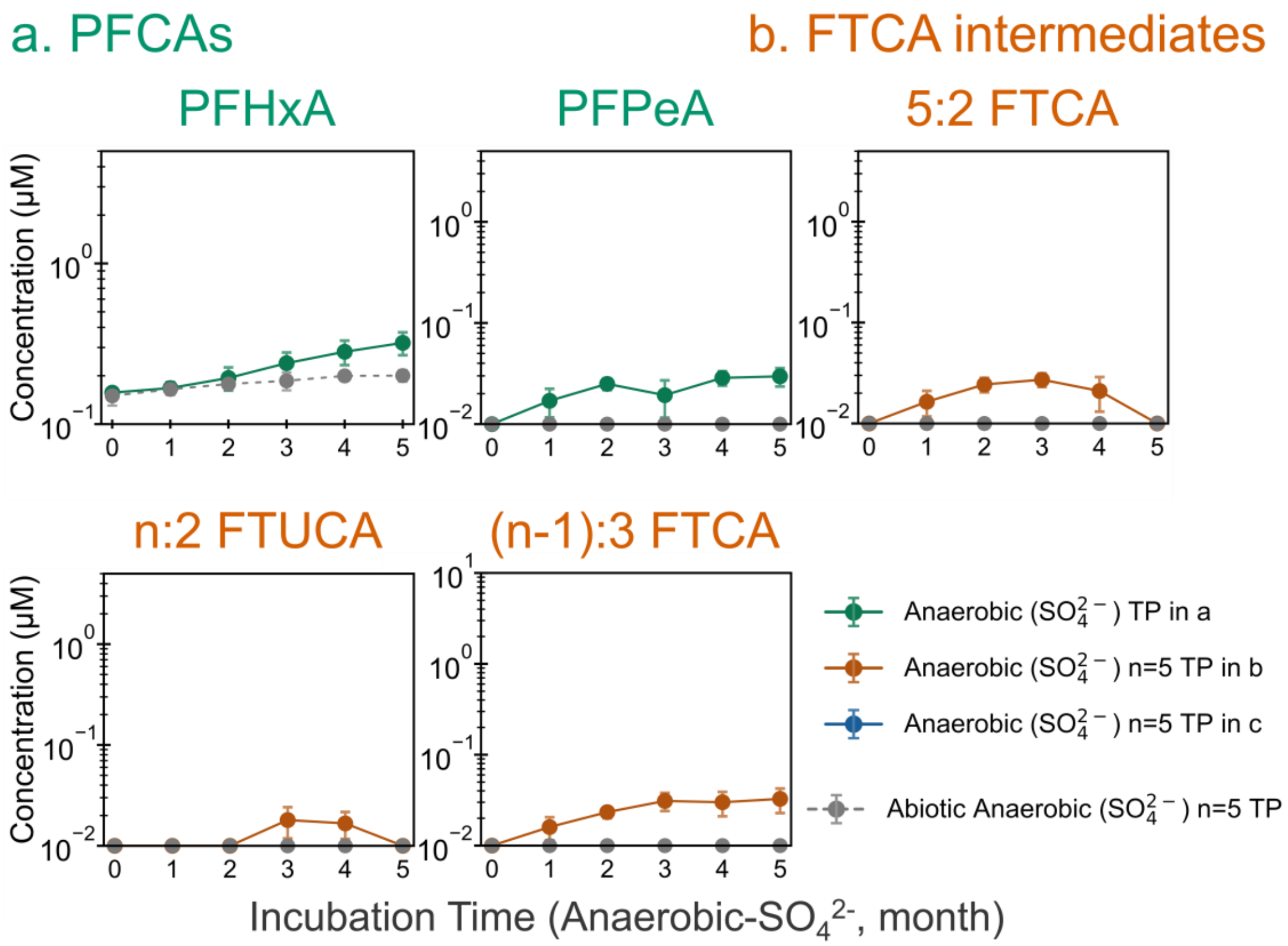

Figure S6. Temporal concentration profiles of 5:3 FTCA-derived TPs under anaerobic sulfate-reducing conditions based on semi-quantitative measurements.

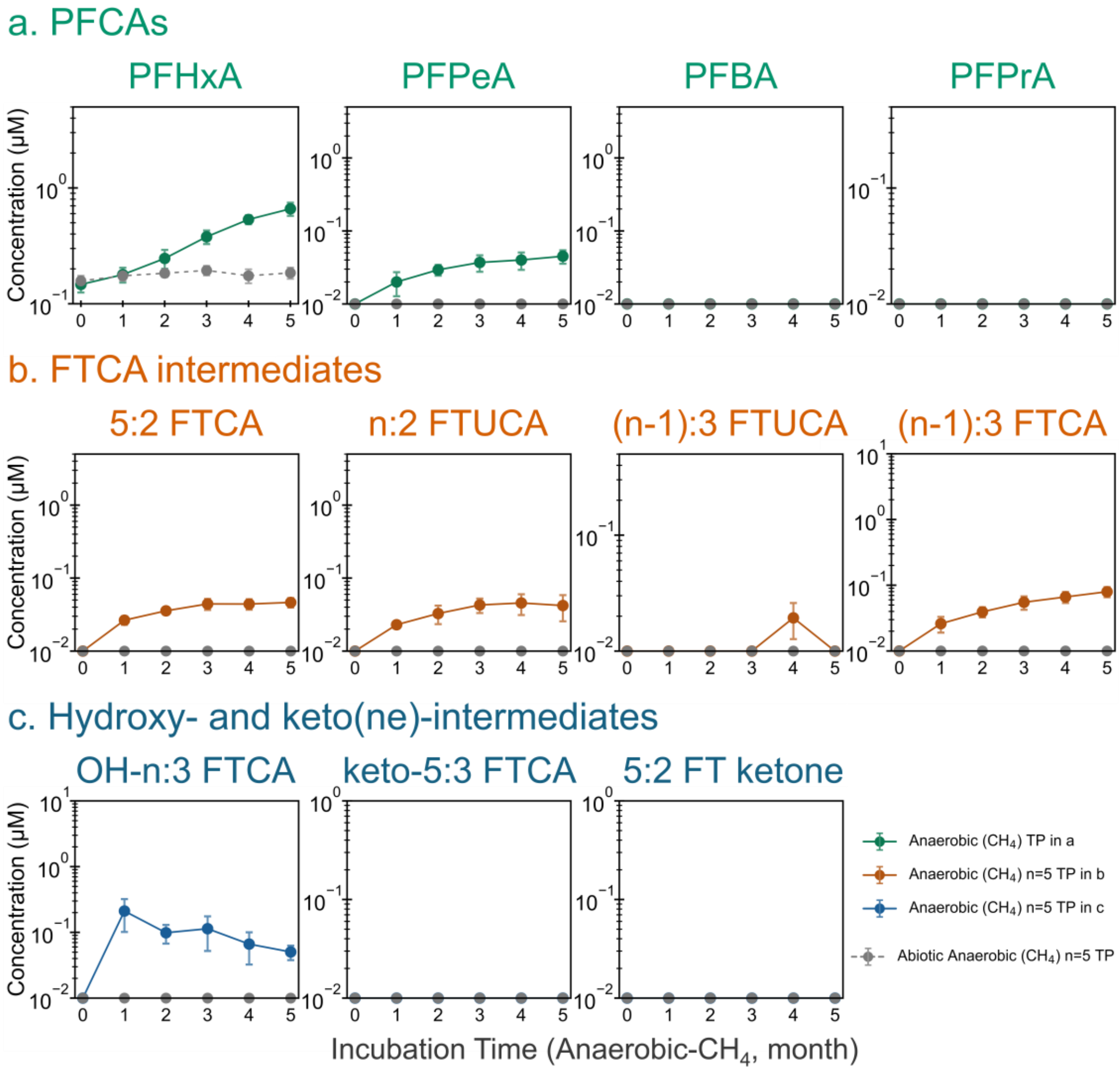

Figure S7. Temporal concentration profiles of 5:3 FTCA-derived TPs under anaerobic methanogenic conditions based on the combination of absolute and semi-quantitative measurements.

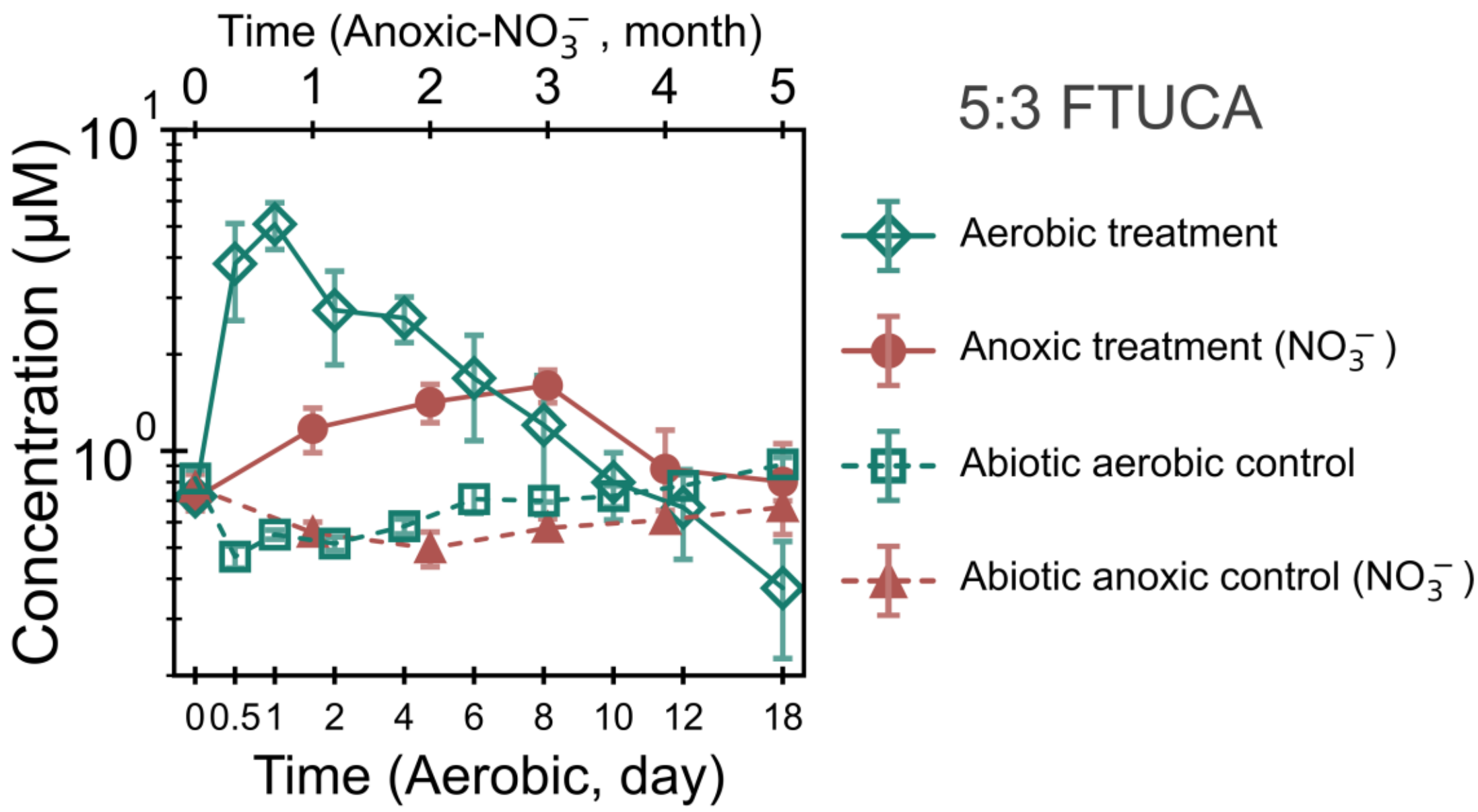

Figure S8. Temporal concentration profiles of 5:3 FTUCA under aerobic versus anoxic nitrate-reducing conditions in log scale, with solid lines represent live treatment and dashed lines represent corresponding abiotic conditions.

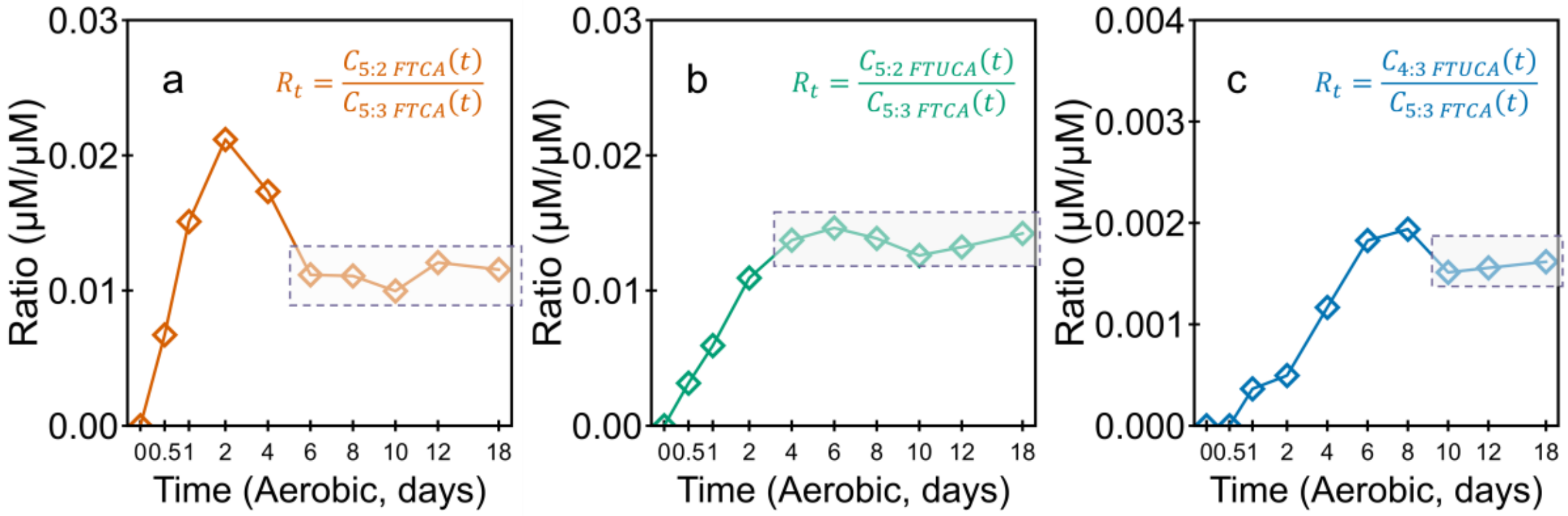

Figure S9. Ratios of three chain-shortening intermediate products, including (a) 5:2 FTCA, (b) 5:2 FTUCA, and (c) 4:3 FTUCA to 5:3 FTCA in aerobic microcosms.

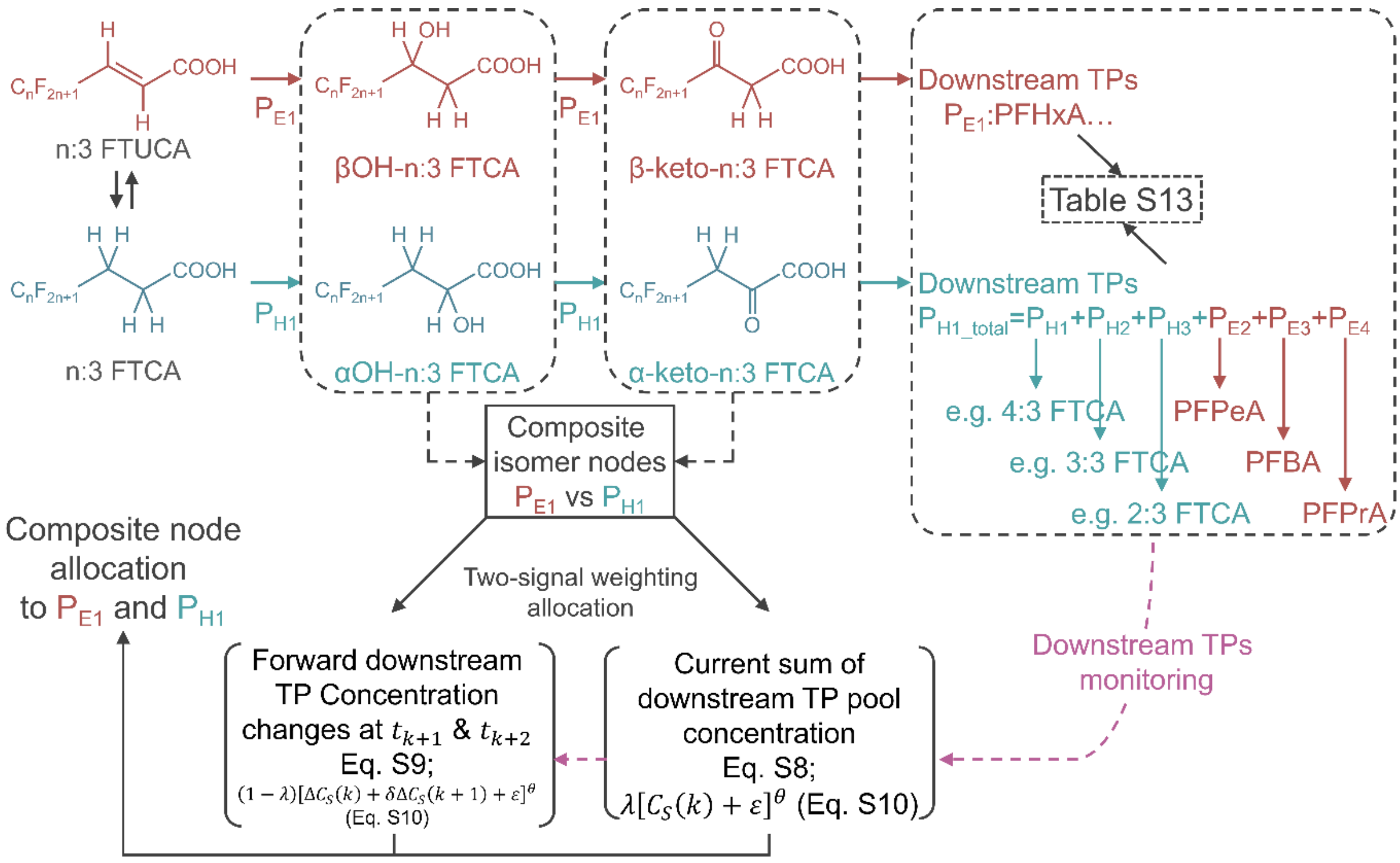

Figure S10. Allocation schematic of composite node isomers (use OH- and keto-5:3 FTCA as examples) to pathway families (P_{E1} and P_{H1} in this case).

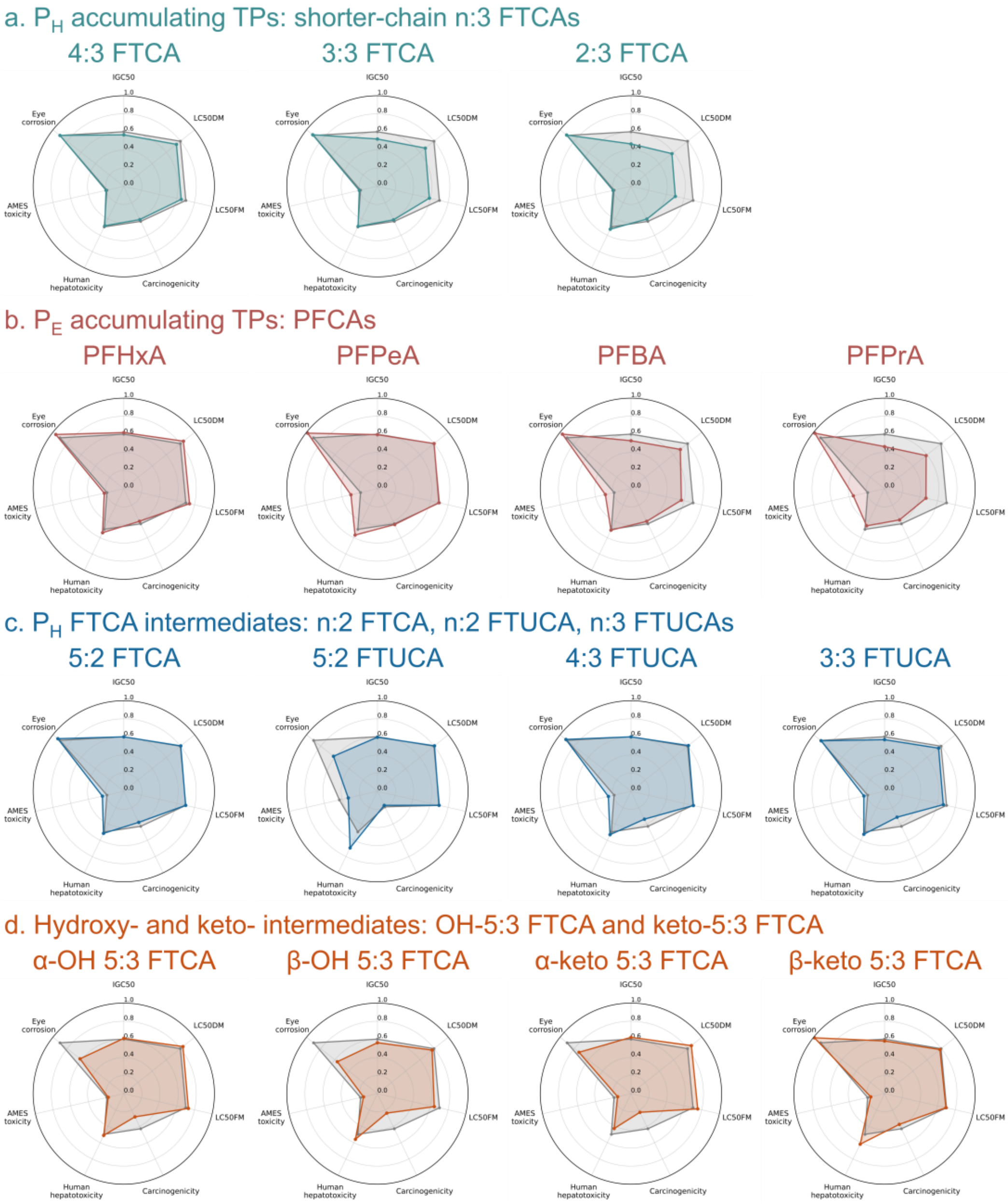

Figure S11. Predicted toxicity profiles of 5:3 FTCA and selected TPs. TPs are grouped into four pathway classes: accumulating shorter-chain n:3 FTCAs, accumulating PFCAs, FTCA/FTUCA intermediates, and hydroxy-/keto-FTCA intermediates. Grey polygons represent parent 5:3 FTCA, and colored polygons represent individual TPs. The radar plots compare selected ADMETlab 3.0 toxicity indicators, including IGC50, LC50DM, LC50FM, carcinogenicity, human hepatotoxicity, AMES toxicity, and eye corrosion. The concentration-derived aquatic toxicity endpoints IGC50, LC50DM, and LC50FM were scaled by dividing the ADMETlab-reported -log10 transformed values by 6.

Table S1. List of isotope-labelled and analytical/native PFAS standards

Name	Acronym	$[M\text{-}H]^-$	SMILES
Perfluoro-n-[1,2,3,4-$^{13}C_4$]-butanoic acid	$^{13}C_4$-PFBA	$[^{13}C]_4F_7O_2^-$	[13C](=O)([13C]([13C]([13C](F)(F)F)(F)F)(F)F)O
Perfluoro-n-[1,2,3,4,5-$^{13}C_5$]-pentanoic acid	$^{13}C_5$-PFPeA	$[^{13}C]_5F_9O_2^-$	[13C](=O)([13C]([13C]([13C]([13C](F)(F)F)(F)F)(F)F)(F)F)O
Perfluoro-n-[1,2,3,4,6-$^{13}C_5$]-hexanoic acid	$^{13}C_5$-PFHxA	$C[^{13}C]_5F_{11}O_2^-$	[13C](=O)([13C]([13C]([13C](C([13C](F)(F)F)(F)F)(F)F)(F)F)(F)F)O
2H,2H,3H,3H-Perfluoro-n-[1,2,3-$^{13}C_3$]-octanoic acid	$^{13}C_3$-5:3 FTCA	$C_5[^{13}C]_3H_4F_{11}O_2^-$	[13CH2]([13CH2]C(C(C(C(C(F)(F)F)(F)F)(F)F)(F)F)(F)F)[13C](=O)O
Perfluorobutyric acid	PFBA	$C_4F_7O_2^-$	C(=O)(C(C(C(F)(F)F)(F)F)(F)F)O
Perfluoropentanoic acid	PFPeA	$C_5F_9O_2^-$	C(=O)(C(C(C(C(F)(F)F)(F)F)(F)F)(F)F)O
Perfluorohexanoic Acid	PFHxA	$C_6F_{11}O_2^-$	C(=O)(C(C(C(C(C(F)(F)F)(F)F)(F)F)(F)F)(F)F)O
2H,2H,3H,3H-Perfluorooctanoic acid	5:3 FTCA	$C_8H_4F_{11}O_2^-$	C(CC(C(C(C(C(F)(F)F)(F)F)(F)F)(F)F)(F)F)C(=O)O

Table S2. Characteristics of WWTPs for activated sludge and anaerobic sludge sample collection in this study. MGD, million gallons per day.

**WWTP**	**P**		
Service Scale	*Population served*	*Hydraulic scale*	*Sampling location*
~150 mi^2 (390 km^2) across ~48 municipalities	~1.4-1.5 million residents	Design capacity at ~330 MGD, with long-term avg. influent ~220-250 MGD; Wet weather capacity at ~400 MGD	Anaerobic digester
Wastewater and co-waste influent	Mixed municipal wastewater (residential and commercial sewage, pretreated industrial discharges, liquid wastes, and landfill leachate)		

**WWTP**	**L**		
Service Scale	*Population served*	*Hydraulic scale*	*Sampling location*
~13 mi^2 (34 km^2) across 2 municipalities	~60,000 residents and an industrial community	Design capacity at ~17 MGD	Anaerobic digester
Wastewater and co-waste influent	Domestic sewage with industrial wastewater subject to pretreatment; industrial discharges		

**WWTP**	**R**		
Service Scale	*Population served*	*Hydraulic scale*	*Sampling location*
~48 mi^2 (124 km^2); primary residential and light commercial and industrial area.	~250,000 residents and ~3,000 commercial/industrial customers	Design capacity at ~40 MGD, with daily avg. influent ~30 MGD	Aeration tank<br>Anaerobic digester

Wastewater and co-waste influent	Mixed domestic sewage (suburban residential use) and pretreated industrial/commercial wastewater

Table S3. Experimental setup for aerobic and anoxic/anaerobic FTCA biotransformation assays

Experimental Group (Redox Condition)	*Inoculum Source*	*Medium Matrix*	*Electron acceptor; headspace*	*Initial FTCA Conc. (μM)*
Nitrate-Reducing Treatment	Mixed anaerobic digester sludge (WWTP L/R/P)	Sulfate-free AMS	20 mM NO_3^-; N_2 headspace	80
Sulfate-Reducing Treatment			20 mM SO_4^{2-}; N_2 headspace	
Methanogenic Treatment			50:50 CO_2/N_2 headspace	
Aerobic Treatment-R1	Activated sludge (WWTP R Tank R1)	Synthetic wastewater	Air headspace; shaken (130 rpm)	80
Aerobic Treatment-R2	Activated sludge (WWTP R Tank R2)		Air headspace; shaken (130 rpm)	
Aerobic Treatment-R3	Activated sludge (WWTP R Tank R3)		Air headspace; shaken (130 rpm)	
Nitrate-Reducing Abiotic Control	Autoclaved mixed anaerobic digester sludge (WWTP L/R/P)	Sulfate-free AMS	20 mM NO_3^-; N_2 headspace	80
Sulfate-Reducing Abiotic Control			20 mM SO_4^{2-}; N_2 headspace	
Methanogenic Abiotic Control			50:50 CO_2/N_2 headspace	
Aerobic Abiotic Control	Autoclaved mixture of R1-R3 sludge	Synthetic wastewater	Air headspace; shaken (130 rpm)	80
Nitrate-Reducing Microbial Control	Mixed anaerobic digester sludge (WWTP L/R/P)	Sulfate-free AMS	20 mM NO_3^-; N_2 headspace	-
Aerobic Microbial Control	Activated sludge mixture (WWTP R Tank R1-R3)	Synthetic wastewater	Air headspace; shaken (130 rpm)	-
Anaerobic Analytical Control	-	Sulfate-free AMS	-	80
Aerobic Analytical Control	-	Synthetic wastewater	Air headspace; shaken (130 rpm)	80

Table S4. LC operating conditions and gradient program

Agilent 1290 Infinity II LC<br>Column: Waters Symmetry C18 Column Column Temp: 50°C<br>Inject Volume: 10μL Maximum Pressure: 900 bars				
Segment	Time (min)	Eluent A (%)	Eluent B (%)	Flow Rate (mL/min)
Run	0	95	5	0.30
	0.5	95	5	0.35
	3.5	70	30	0.35
	8	5	95	0.35
	9	5	95	0.35
	9.5	95	5	0.35
Post-run	11.5	95	5	0.30

Table S5. LC/MS/MS multiple reaction monitoring (MRM) transitions and settings

Native and internal standard	Precursor ion (*m/z*, $[M-H]^-$)	Product ion(s) (*m/z*)	Collision Energy (V)	Fragmentor (V)	Cell Accel. (V)
5:3 FTCA	341.0	237.0; 217.0	20; 25	60	3
PFHxA	313.0	269.0; 119.0	12; 24	56	2
PFPeA	263.0	219.0	4	76	2
PFBA	213.0	168.9	4	48	2
M3-5:3 FTCA	344.0	239.0; 219.0	20; 25	60	3
M5-PFHxA	318.0	273.0; 120.0	12; 24	56	2
M5-PFPeA	268.0	223.0	4	76	2
M4-PFBA	217.0	172.0	4	48	2

Table S6. Identification of PFAS confidence level (CLs) according to Charbonnet et al., 2022 [10] and Boatman et al., 2025 [11]

Identification Level	Claiming expectation	Evidence required
1a	Confirmed by reference standard	*MS1 evidence*: Exact mass; mass defect; isotopic pattern<br>*MS2 evidence*: Library MS2
1b	Isomers indistinguishable from reference standard	*MS1 evidence*: Exact mass; mass defect; isotopic pattern<br>*MS2 evidence*: Library MS2
2a	Probable by library spectrum match	*MS1 evidence*: Exact mass; mass defect; isotopic pattern<br>*MS2 evidence*: Diagnostic fragments from library MS2
2b	Probable by diagnostic fragmentation	*MS1 evidence*: Exact mass; mass defect; isotopic pattern; (homolog identified)<br>*MS2 evidence*: ≥3 diagnostic fragments
2c	Probable by homolog	*MS1 evidence*: Exact mass; mass defect; isotopic pattern; 2 or more homolog identified ≥2a<br>*MS2 evidence*: ≥2 diagnostic fragments
3a	Positional isomer candidates	*MS1 evidence*: Exact mass; mass defect; isotopic pattern; (homolog identified)<br>*MS2 evidence*: ≥1 subclass-aligned fragment
3b	Fragmentation-inferred candidates	*MS1 evidence*: Exact mass; mass defect; isotopic pattern<br>*MS2 evidence*: PFAS subclass fragments
3c	"Circumstantial" PFAS candidates with fragments evidence	*MS1 evidence*: Exact mass; mass defect; isotopic pattern; homolog identified

		MS2 evidence: PFAS subclass fragmentation; circumstantial evidence
3d	"Circumstantial" PFAS candidates with homolog evidence	*MS1 evidence*: Exact mass; mass defect; isotopic pattern; homolog identified<br>*MS2 evidence*: None

Table S7. Analytical standards and isotope-labeled internal standards used for target quantification and Nano-ESI-HRMS semi-quantification.

Analyte/Analyte group	Quantification method	Analytical standard	Isotope-labeled standard
5:3 FTCA	LC/MS/MS; absolute quantification	5:3 FTCA	$^{13}C_3$-5:3 FTCA
PFHxA, PFPeA, PFBA		PFHxA, PFPeA, PFBA	$^{13}C_5$-PFHxA, $^{13}C_5$-PFPeA, $^{13}C_4$-PFBA,
5:3 FTCA and other FTCA intermediates	Nano-ESI-HRMS; Semi-quantification	5:3 FTCA	$^{13}C_3$-5:3 FTCA
PFHxA, PFPeA, PFBA		PFPeA	$^{13}C_5$-PFPeA
PFPrA, TFA		PFPrA	

Table S8. LOQ/MDL for calibration/native standards in Nano-ESI-HRMS analysis

Calibration standard	LOQ (ppb)	MDL (ppb)	Sample-level LOQ (before dilution; μM)	Analyte group
5:3 FTCA	0.107	0.042	0.016	FTCA intermediates
PFPeA	0.050	0.018	0.010	PFCAs (C4-C6)
PFPrA	0.050	0.013	0.015	PFCAs (C2-C3)

Table S9. Calculated MDLs for NO_3^- and SO_4^{2-} measurement by IC, determined following EPA Method 300.0 [12] from 7 replicate laboratory fortified blanks. The MDLs in the injected samples were calculated as $MDL = t_{6,99\%} \times \sigma$, where σ is the standard deviation of replicate measurements. The MDL in the original microcosm sample was multiplied by dilution factor (20×).

Analyte	MDLs in injected solutions	Corresponding concentrations in original microcosm samples
NO_3^-	11.6 μg·L^{-1}	232 μg·L^{-1}
SO_4^{2-}	9.1 μg·L^{-1}	182 μg·L^{-1}

Table S10. List of biotransformation TP with their formula, observed *m/z*, mass error, identification level, regimes found and detection method. "Ae" represents aerobic microcosms, while "An" represents anoxic nitrate-reducing microcosms.

TPs	Mol. Formula	Theor. Anion *m/z*	Observed Anion *m/z*	Mass error (ppm)	Time point of max. quant. Conc.	Conf. level	Regime Detected	Detection method
5:3 FTUCA	$C_8H_3F_{11}O_2$	338.98791	338.98856	1.92	3 m (An)<br>24 h (Ae)	2b	N/C/S<br>Aerobic	Nano-ESI-HRMS
PFHxA	$C_6HF_{11}O_2$	312.97226	312.97273	1.50	5 m (An)<br>18 d (Ae)	1a	N/C/S<br>Aerobic	LC-MS/MS &<br>Nano-ESI-HRMS
OH-5:3 FTCA	$C_8H_5F_{11}O_3$	356.99848	356.99936	2.46	1 m (An)<br>48 h (Ae)	3a	N/C/S<br>Aerobic	Nano-ESI-HRMS
keto-5:3 FTCA	$C_8H_3F_{11}O_3$	354.98283	354.98374	2.56	2 m (An)<br>48 h (Ae)	3a	N<br>Aerobic	Nano-ESI-HRMS
5:2 FT ketone	$C_7H_3F_{11}O$	310.99300	310.99332	1.03	1 m (An)<br>12 d (Ae)	2b	N<br>Aerobic	Nano-ESI-HRMS

5:2 FTCA	$C_7H_3F_{11}O_2$	326.98791	326.98852	1.87	5 m (An)<br>48 h (Ae)	2b	N/C/S<br>Aerobic	Nano-ESI-HRMS
5:2 FTUCA	$C_7H_2F_{10}O_2$	306.98169	306.98245	2.48	4 m (An)<br>96 h (Ae)	3	N/C/S<br>Aerobic	Nano-ESI-HRMS
3-F 4:3 FTCA	$C_7H_4F_{10}O_2$	308.99734	308.99769	1.13	-<br>6 d (Ae)	3d	Aerobic	Nano-ESI-HRMS
4:3 FTUCA	$C_7H_3F_9O_2$	288.99111	288.99168	1.97	4 m (An)<br>6 d (Ae)	2b	N<br>Aerobic	Nano-ESI-HRMS
4:3 FTCA	$C_7H_5F_9O_2$	291.00676	291.00748	2.47	5 m (An)<br>18 d (Ae)	2b	N/C<br>Aerobic	Nano-ESI-HRMS
OH-4:3 FTCA	$C_7H_5F_9O_3$	307.00167	307.00251	2.74	1 m (An)<br>18 d (Ae)	3	N<br>Aerobic	Nano-ESI-HRMS
keto-4:3 FTCA	$C_7H_3F_9O_3$	304.98602	304.98683	2.66	-<br>10 d (Ae)	3d	Aerobic	Nano-ESI-HRMS

4:2 FT ketone	$C_6H_3F_9O$	260.99619	260.99663	1.69	-<br>8 d (Ae)	3d	Aerobic	Nano-ESI-HRMS
PFPeA	$C_5HF_9O_2$	262.97546	262.97582	1.37	5 m (An)<br>18 d (Ae)	1a	N/C<br>Aerobic	LC-MS/MS &<br>Nano-ESI-HRMS
4:2 FTCA	$C_6H_3F_9O_2$	276.99111	276.99167	2.02	-<br>-	3d	Aerobic	Nano-ESI-HRMS
4:2 FTUCA	$C_6H_2F_8O_2$	256.98488	256.98555	2.61	-<br>18 d (Ae)	2b	Aerobic	Nano-ESI-HRMS
3-F 3:3 FTCA	$C_6H_4F_8O_2$	259.00053	259.00080	1.04	-<br>18 d (Ae)	2b	Aerobic	Nano-ESI-HRMS
3:3 FTUCA	$C_6H_3F_7O_2$	238.99430	238.99477	1.97	-<br>18 d (Ae)	2b	Aerobic	Nano-ESI-HRMS
3:3 FTCA	$C_6H_5F_7O_2$	241.00995	241.01029	1.41	-<br>18 d (Ae)	2b	Aerobic	Nano-ESI-HRMS

PFBA	$C_4HF_7O_2$	212.97865	212.97871	0.28	5 m (An)<br>18 d (Ae)	1a	N/C<br>Aerobic	LC-MS/MS &<br>Nano-ESI-HRMS
2:3 FTCA	$C_5H_5F_5O_2$	191.01315	191.01302	-0.68	-<br>18 d (Ae)	3d	Aerobic	Nano-ESI-HRMS
PFPrA	$C_3HF_5O_2$	162.98185	162.98156	-1.78	-<br>18 d (Ae)	1a	Aerobic	Nano-ESI-HRMS
TFA	$C_2HF_3O_2$	112.98504	112.98454	-4.42	-<br>-	3	Aerobic	Nano-ESI-HRMS

Table S11. First timepoint of significant emergence relative to abiotic controls and *p*-values for quantified TPs using Welch's t-test

	Emergence time (Aerobic)	*p*-value	Emergence time (Anoxic)	*p*-value
OH-5:3 FTCA	12 h	0.0235	1 m	0.0070
5:3 FTUCA	12 h	0.0197	1 m	0.0074
keto-5:3 FTCA	48 h	0.0025	2 m	0.0039
PFHxA	48 h	0.0162	1 m	0.0023
5:2 FTCA	12 h	0.0076	1 m	0.0025
5:2 FTUCA	12 h	0.0204	1 m	0.0090
4:3 FTUCA	24 h	0.0020	1 m	0.0021
4:3 FTCA	24 h	0.0015	1 m	0.0059
OH-4:3 FTCA	48 h	0.0057	1 m	0.0037
PFPeA	12 h	0.0113	1 m	0.0075
4:2 FTUCA	6 d	0.0001	-	-
3:3 FTUCA	6 d	0.0025	-	-
3:3 FTCA	48 h	0.0147	-	-
PFBA	12 h	0.0032	4 m	0.0043
2:3 FTCA	10 d	0.0032	-	-
PFPrA	96 h	0.0003	-	-

Table S12. Time-integrated area under the abiotic-corrected concentration-time curve (AUC) of detected TPs over selected monitoring periods

AUC_j	Anoxic [0, 2 month] μM · m	Anoxic [0, 5 month] μM · m	Aerobic [0, 96 h] μM · d	Aerobic [0, 18 d] μM · d
OH-5:3 FTCA	0.555	1.106	10.085	26.525
5:3 FTUCA	2.639	7.028	13.738	30.853
keto-5:3 FTCA	0.042	0.060	0.115	0.149
PFHxA	0.466	2.256	0.809	7.213
5:2 FTCA	0.044	0.181	3.636	9.332
5:2 FTUCA	0.056	0.220	2.010	8.576
4:3 FTUCA	0.071	0.257	0.117	0.920
4:3 FTCA	0.048	0.271	2.055	38.524
OH-4:3 FTCA	0.037	0.054	0.114	1.219
PFPeA	0.050	0.266	2.782	33.046
3:3 FTUCA	0	0	0	0.603
3:3 FTCA	0	0	0.260	5.104
4:2 FTUCA	0	0	0	0.668
PFBA	0	0.059	1.233	18.113
2:3 FTCA	0	0	0	0.548
PFPrA	0	0	0.035	1.864

Table S13. Pathway and cascade assignments for quantitative TPs. "Low Conc." indicates a low intensity compound assigned at CL ≤ 3d and that not quantified. "ND" indicates not detected or below LOQ.

TP (j)	Pathway/Cascade Assignment	Note
5:3 FTUCA	-	Starting enoyl acid of P_{E1}; not assigned
PFHxA	P_{E1}	
5:2 FT ketone	P_{E1}	Low Conc.; not assigned
5:2 sFTOH	P_{E1}	ND; not assigned
OH-5:3 FTCA	P_{E1}/P_{H1} allocation	Composite α/β node
keto-5:3 FTCA	P_{E1}/P_{H1} allocation	Composite α/β node
5:2 FTCA	P_{H1}	
5:2 FTUCA	P_{H1}	
3-F 4:3 FTCA	P_{H1}	Low Conc.; not assigned
4:3 FTUCA	P_{H1}	Starting of 4:3 layer (P_{E2})
4:3 FTCA	P_{H1}	Starting of 4:3 layer (P_{H2})
OH-4:3 FTCA	P_{E2}/P_{H2} allocation	Composite α/β node
keto-4:3 FTCA	P_{E2}/P_{H2} allocation	Composite α/β node;<br>Low Conc.; not assigned
4:2 FT ketone	P_{E2}	ND
4:2 sFTOH	P_{E2}	ND
PFPeA	P_{E2}	
4:2 FTCA	P_{H2}	Low Conc.; not assigned
4:2 FTUCA	P_{H2}	
3-F 3:3 FTCA	P_{H2}	Low Conc.; not assigned
3:3 FTUCA	P_{H2}	Starting of 3:3 layer (P_{E3})
3:3 FTCA	P_{H2}	Starting of 3:3 layer (P_{H3})
PFBA	P_{E3}	

2:3 FTUCA	P_{H3}	ND; Starting of 2:3 layer (P_{E4})
2:3 FTCA	P_{H3}	Starting of 2:3 layer (P_{H4})
PFPrA	P_{E4}	
TFA	P_{E5}	Low Conc.; not assigned

Table S14. Weighting factor λ and δ for allocation

Aerobic	12 h	24 h	48 h	96 h	6 d	8 d	10 d	12 d	18d
λ, δ	0.6, 1	0.6, 1	0.6, 1	0.6, 1	0.5, 1	0.5, 1	0.5, 1	0.5, 0	1, 0
Anoxic/ Anaerobic	1 m	2 m	3 m	4 m	5 m				
λ, δ	0.6, 1	0.6, 1	0.6, 1	0.6, 0	1, 0				

Table S15. Detailed Predicted toxicity profiles of 5:3 FTCA and selectedTPs.

PFAS	IGC50	LC50DM	LC50FM	AMES toxicity	Human hepatotoxicity	Carcinogenicity
5:3 FTCA	3.61	4.786	4.214	0.191	0.498	0.43
PFHxA	3.721↑	5.055↑	4.464↑	0.221↑	0.539↑	0.399↓
PFPeA	3.586-	4.801-	4.179-	0.3↑	0.568↑	0.43-
PFBA	3.183↓	4.176↓	3.424↓	0.29↑	0.508-	0.4↓
PFPrA	2.792↓	3.525↓	2.815↓	0.353↑	0.455↓	0.382↓
4:3 FTCA	3.41↓	4.456↓	3.9↓	0.201-	0.484↓	0.408↓
3:3 FTCA	3.135↓	4.056↓	3.531↓	0.205↑	0.491-	0.411↓
2:3 FTCA	2.809↓	3.471↓	3.011↓	0.203-	0.526↑	0.402↓
α-OH 5:3 FTCA	3.665-	5.013↑	4.391↑	0.177↓	0.511↑	0.284↓
β-OH 5:3 FTCA	3.378↓	4.636↓	3.858↓	0.158↓	0.559↑	0.238↓
α-OH 4:3 FTCA	3.495↓	4.772-	4.118↓	0.186-	0.521↑	0.263↓
β-OH 4:3 FTCA	3.172↓	4.323↓	3.545↓	0.18↓	0.53↑	0.237↓
α-keto 5:3 FTCA	3.732↑	5.116↑	4.534↑	0.151↓	0.427↓	0.228↓
β-keto 5:3 FTCA	3.493↓	4.697-	4.174-	0.156↓	0.619↑	0.376↓
5:2 FTCA	3.61-	4.826-	4.2-	0.242↑	0.516↑	0.381↓
5:2 FTUCA	3.585-	4.83-	4.183-	0.173↓	0.694↑	0.33↓
4:3 FTUCA	3.611-	4.865-	4.236-	0.256↑	0.532↑	0.342↓
3:3 FTUCA	3.42↓	4.582↓	3.991↓	0.234↑	0.527↑	0.318↓

References

1. Wu, C., et al., *Distinctive biotransformation and biodefluorination of 6:2 versus 5:3 fluorotelomer carboxylic acids by municipal activated sludge.* Water Research, 2024. **254**: p. 121431.
2. Gao, P., et al., *Long-term impact of a tetracycline concentration gradient on the bacterial resistance in anaerobic-aerobic sequential bioreactors.* Chemosphere, 2018. **205**: p. 308-316.
3. Yaqoob, S., et al., *Anaerobic Biotransformation and Biodefluorination of 6:2 Fluorotelomer Carboxylic Acid by Biosolids under the Nitrate-Reducing Condition.* ACS ES&T Water, 2025. **5**(5): p. 2412-2420.
4. USEPA, *Nutrient Control Design Manual: State of Technology Review Report*. 2009, U.S. Environmental Protection Agency, Office of Research and Development: Washington, DC.
5. Grady Jr, C.L., et al., *Biological wastewater treatment*. 2011: CRC press.
6. Su, B., H. Chen, and M. Li, *Mycothiol Conjugation Drives Biotransformation and Biodefluorination of Fluorotelomer Carboxylic Acids by Actinomycetota and Sludge Microbiomes.* bioRxiv, 2025: p. 2025.09.24.678248.
7. Cioni, L., et al., *Fluorine Mass Balance, including Total Fluorine, Extractable Organic Fluorine, Oxidizable Precursors, and Target Per- and Polyfluoroalkyl Substances, in Pooled Human Serum from the Tromsø Population in 1986, 2007, and 2015.* Environmental Science & Technology, 2023. **57**(40): p. 14849-14860.
8. Fu, L., et al., *ADMETlab 3.0: an updated comprehensive online ADMET prediction platform enhanced with broader coverage, improved performance, API functionality and decision support.* Nucleic Acids Research, 2024. **52**(W1): p. W422-W431.
9. Faure, G., *Principles of isotope geology. Second edition*. 1986, United States: John Wiley and Sons Inc.,New York, NY; None.
10. Charbonnet, J.A., et al., *Communicating Confidence of Per- and Polyfluoroalkyl Substance Identification via High-Resolution Mass Spectrometry.* Environmental Science & Technology Letters, 2022. **9**(6): p. 473-481.
11. Boatman, A.K., et al., *Updated Guidance for Communicating PFAS Identification Confidence with Ion Mobility Spectrometry.* Environmental Science & Technology, 2025. **59**(33): p. 17711-17721.
12. EPA, U.S. *Method 300.0: Determination of Inorganic Anions by Ion Chromatography*. 1993; Available from: https://www.epa.gov/sites/default/files/2015-08/documents/method_300-0_rev_2-1_1993.pdf.
