# Supplementary material for: Bifurcated Fluorotelomer Carboxylic Acid Biotransformation: Enoyl-Hydration versus Hydroxylation Regulated by Redox Conditions
Source: Environ Sci Technol. 2026 Sep 22;60(39):27917–30. doi: 10.1021/acs.est.6c04089 (PMC13648231; doi:10.1021/acs.est.6c04089)
Supplement: Supplementary file 2 [file es6c04089_si_002.pdf]

Supporting Information – Mass Spectrometry

for

Bifurcated Fluorotelomer Carboxylic Acid Biotransformation:

Enoyl-hydration versus Hydroxylation Regulated by Redox Conditions

Boyuan Su[1], Hao Chen[1], John Washington[2,3], Mengyan Li[1,*]

[1] Department of Chemistry and Environmental Science, New Jersey Institute of Technology, Newark, New Jersey, United States

[2] Department of Geology, University of Georgia, Athens, GA, USA

[3] USEPA, Office of Research and Development, Center for Environmental Measurement and Modeling Retired, Athens, GA, USA

*Address correspondence to Dr. Mengyan Li

ORCID ID: 0000-0002-7664-4836

Email: mengyan.li@njit.edu

Phone: +1-973-642-7095

Fax: +1-973-596-3586

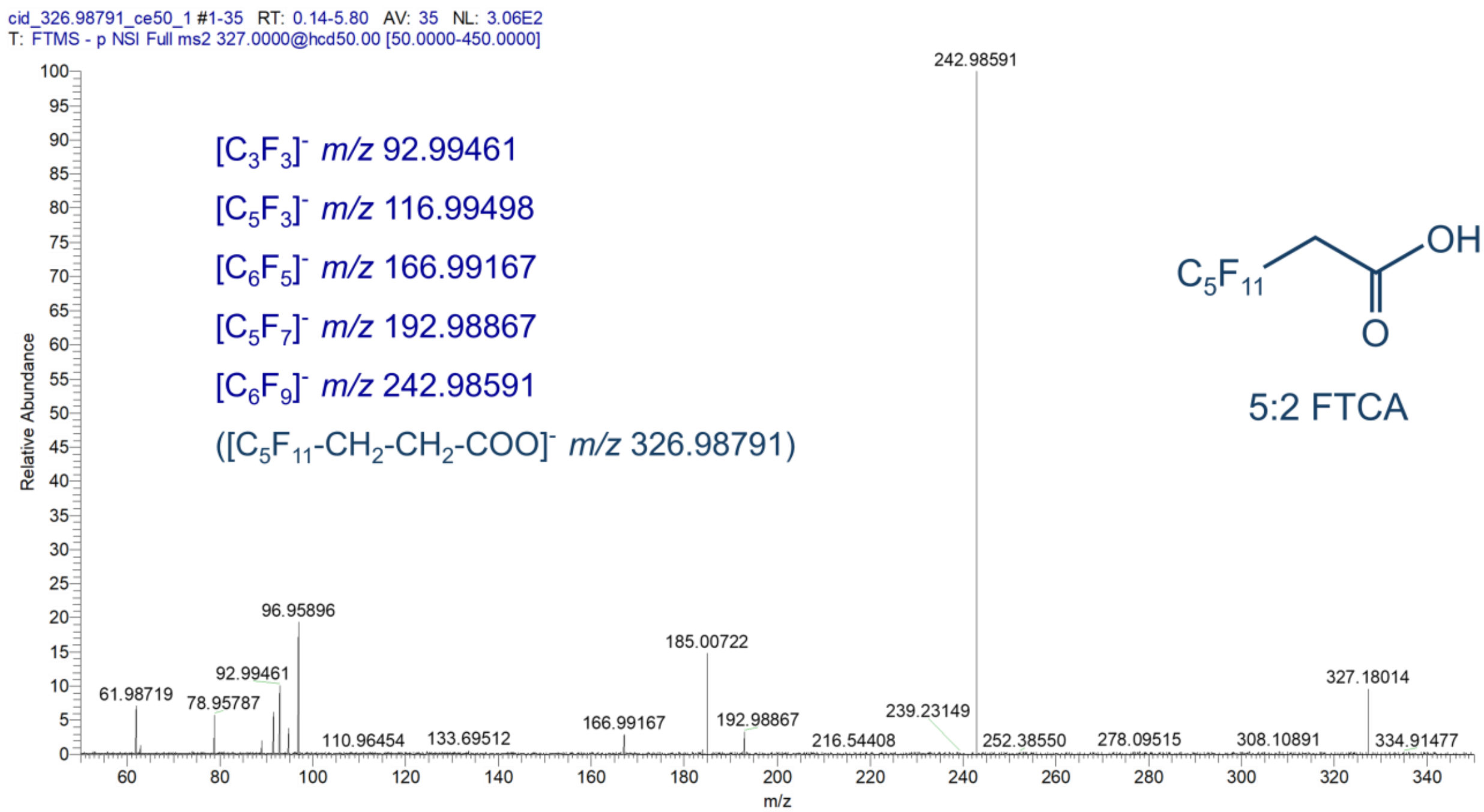

Figure M1. MS2 spectrum of 5:2 FTCA and its major product ions generated by collision-induced dissociation (CID).

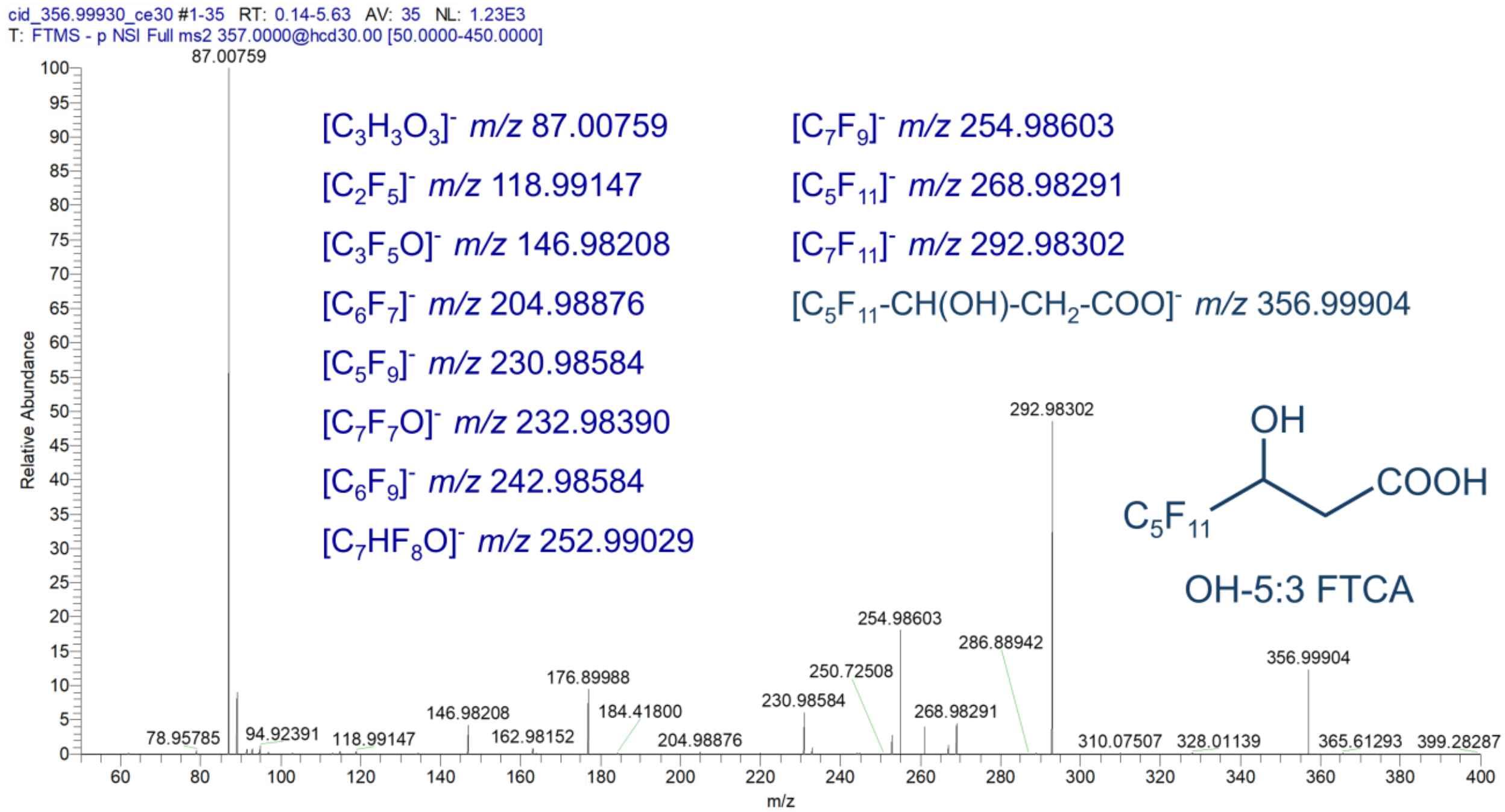

Figure M2. MS2 spectrum of OH-5:3 FTCA and its major product ions generated by CID.

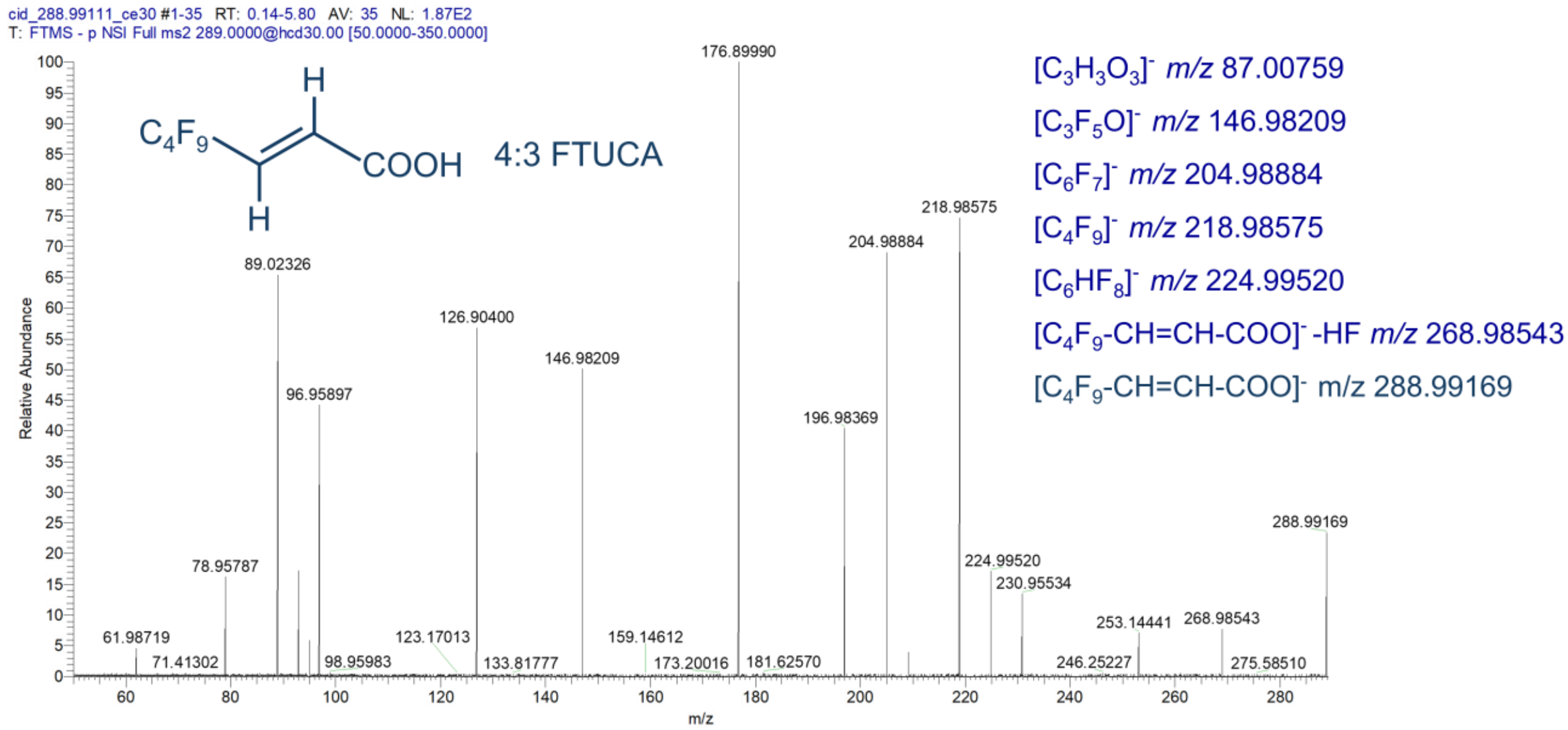

Figure M3. MS2 spectrum of 4:3 FTUCA and its major product ions generated by CID.

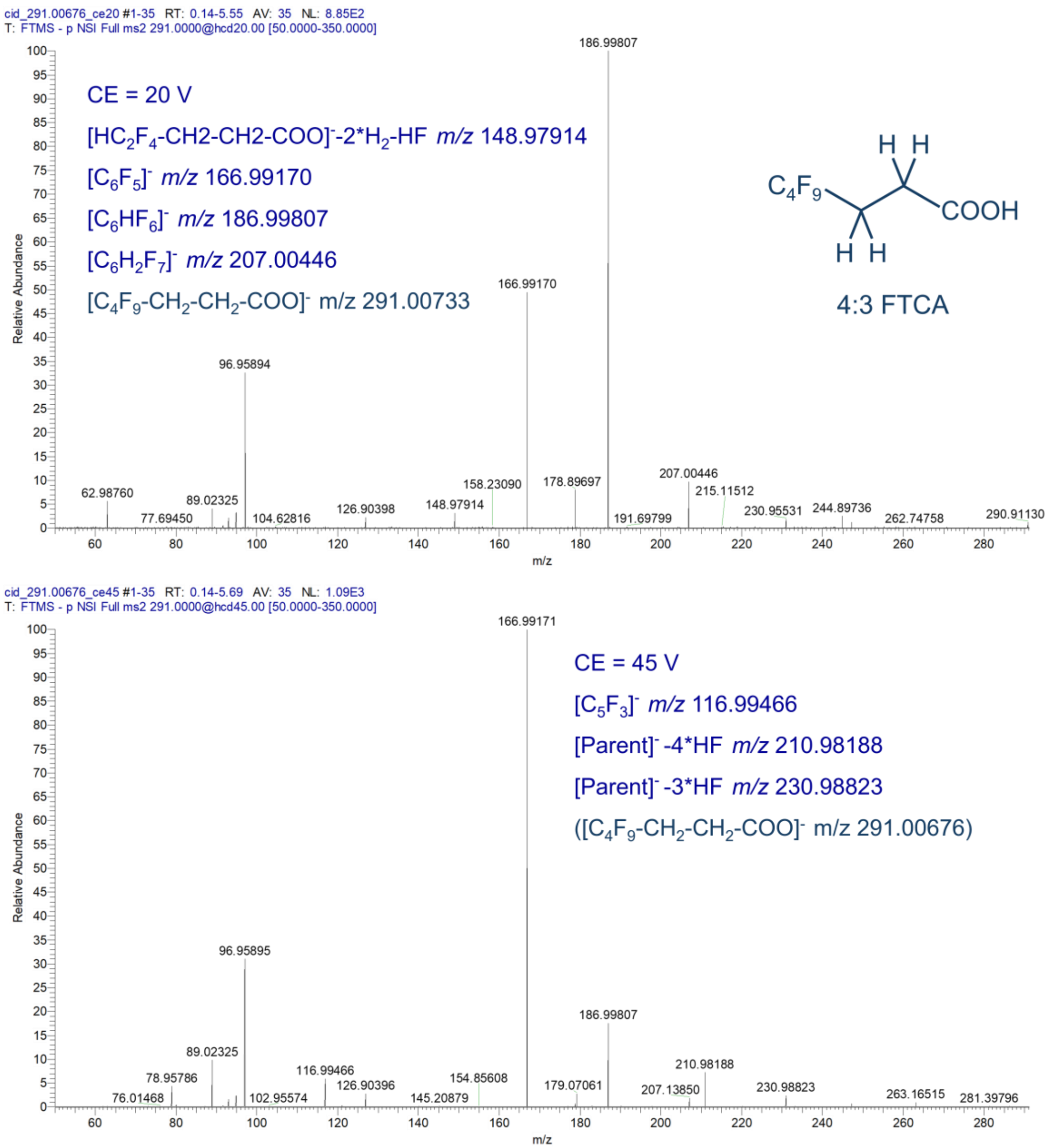

Figure M3. MS2 spectrum of 4:3 FTCA and its major product ions generated by CID.

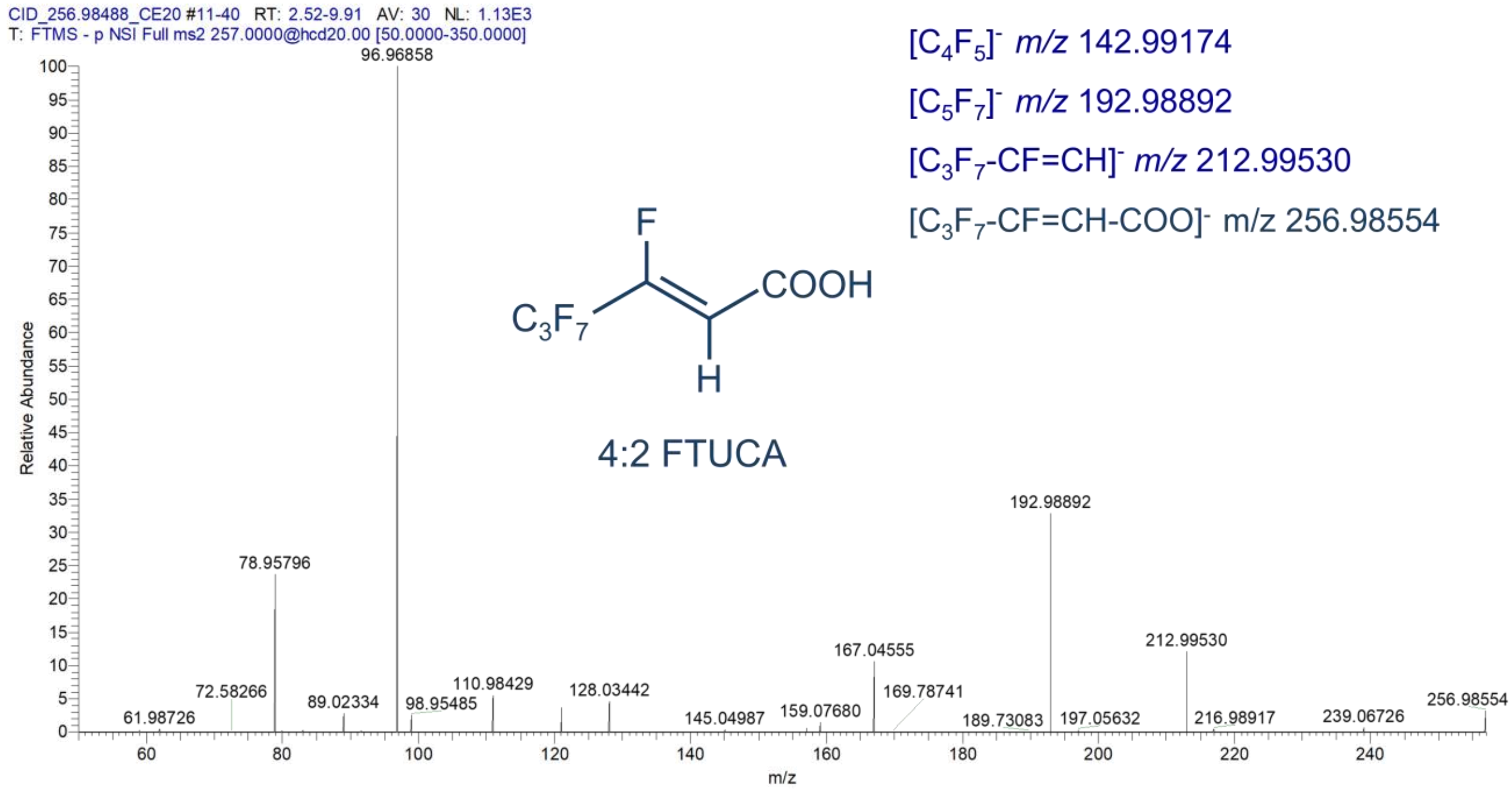

Figure M4. MS2 spectrum of 4:2 FTUCA and its major product ions generated by CID.

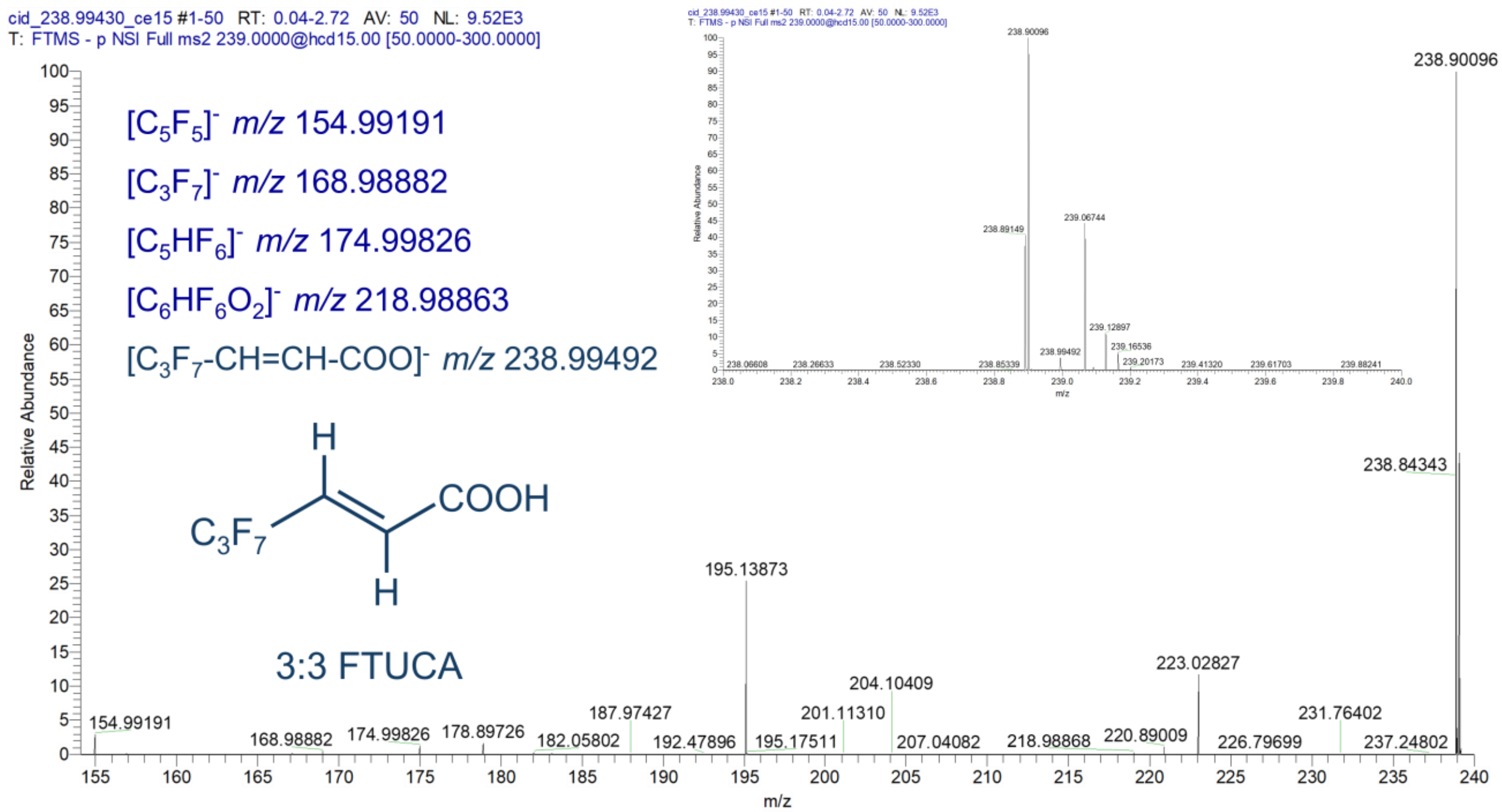

Figure M4. MS2 spectrum of 3:3 FTUCA and its major product ions generated by CID.

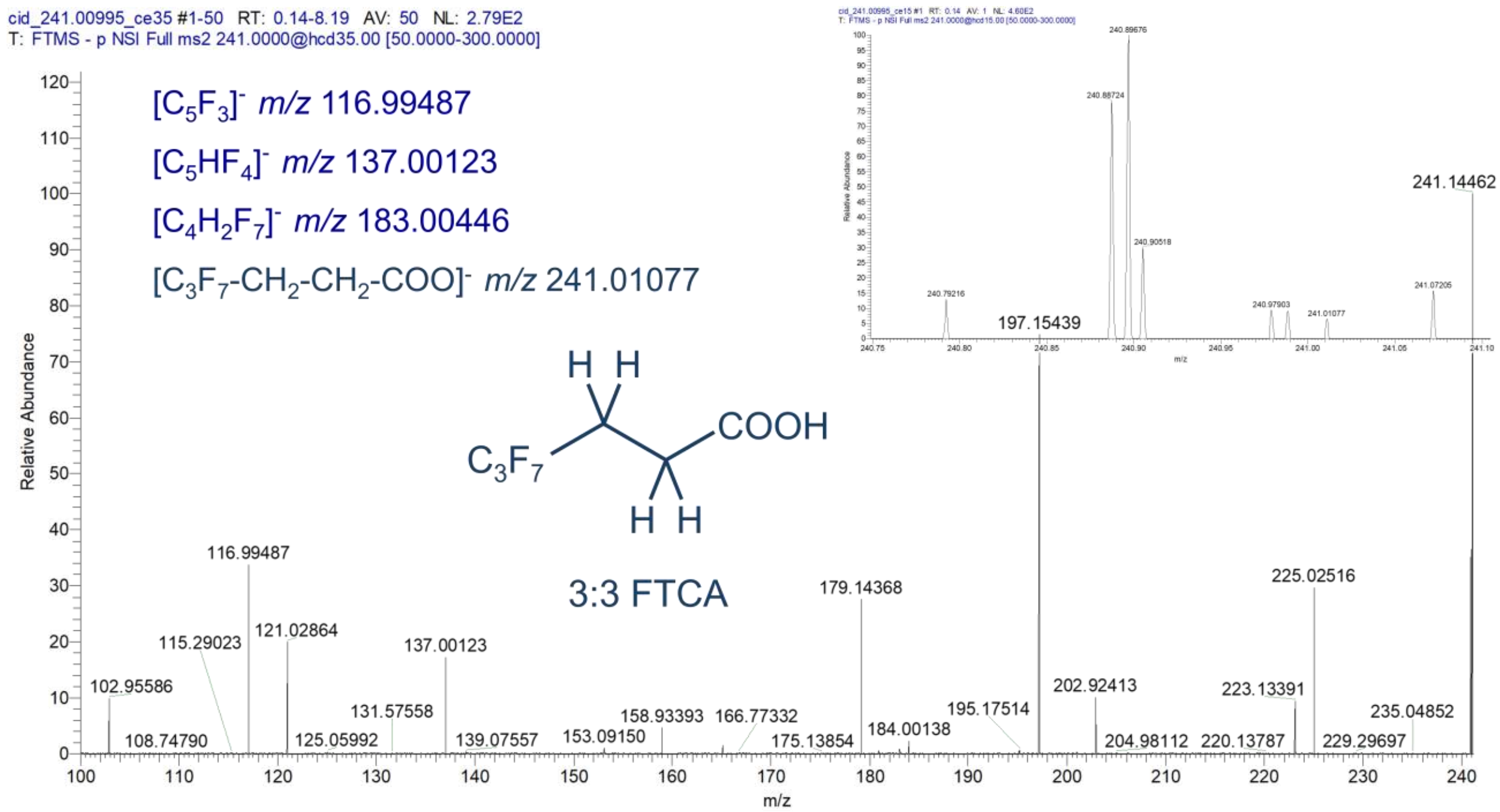

Figure M5. MS2 spectrum of 3:3 FTCA and its major product ions generated by CID.

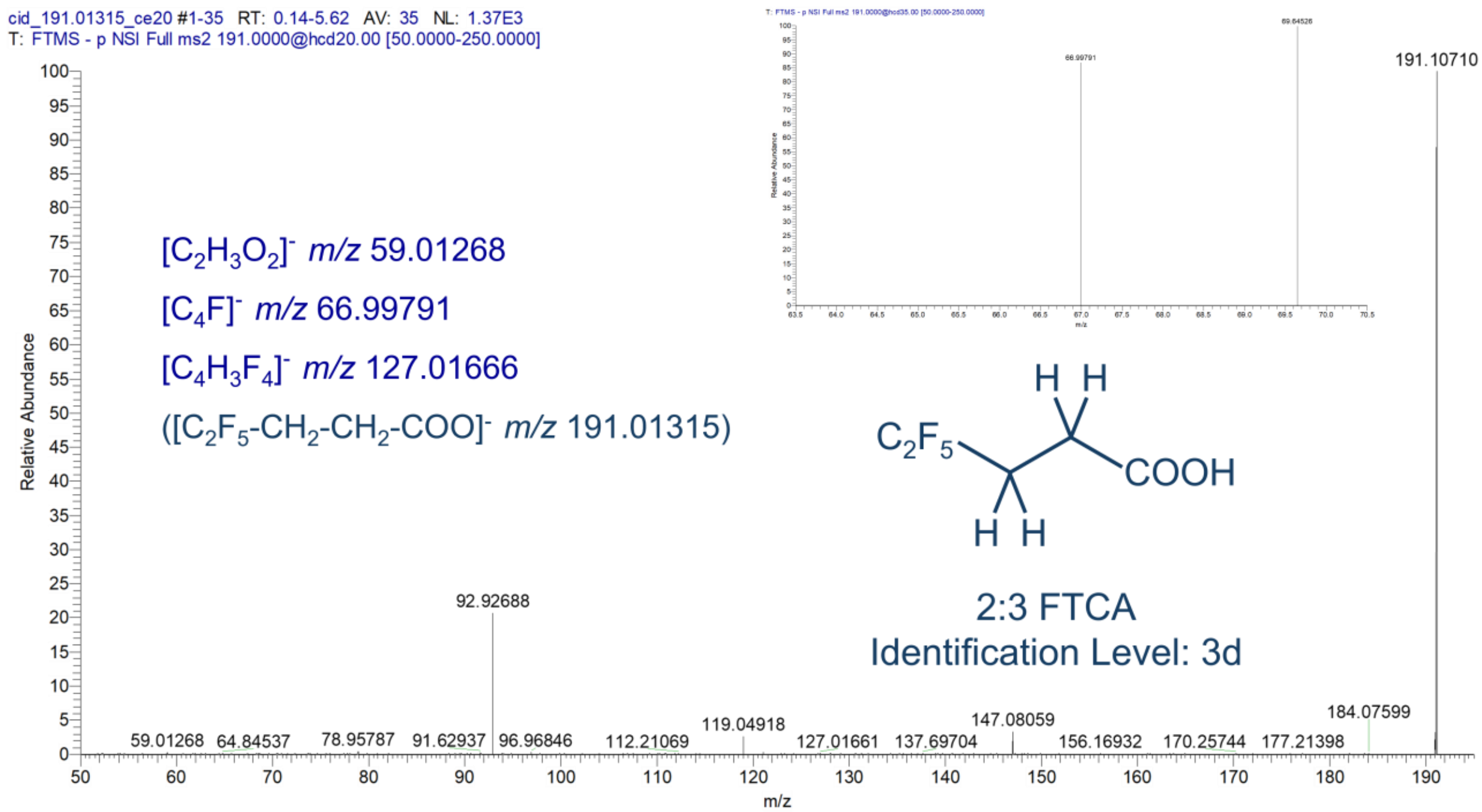

Figure M6. MS2 spectrum of 2:3 FTCA and its major product ions generated by CID.

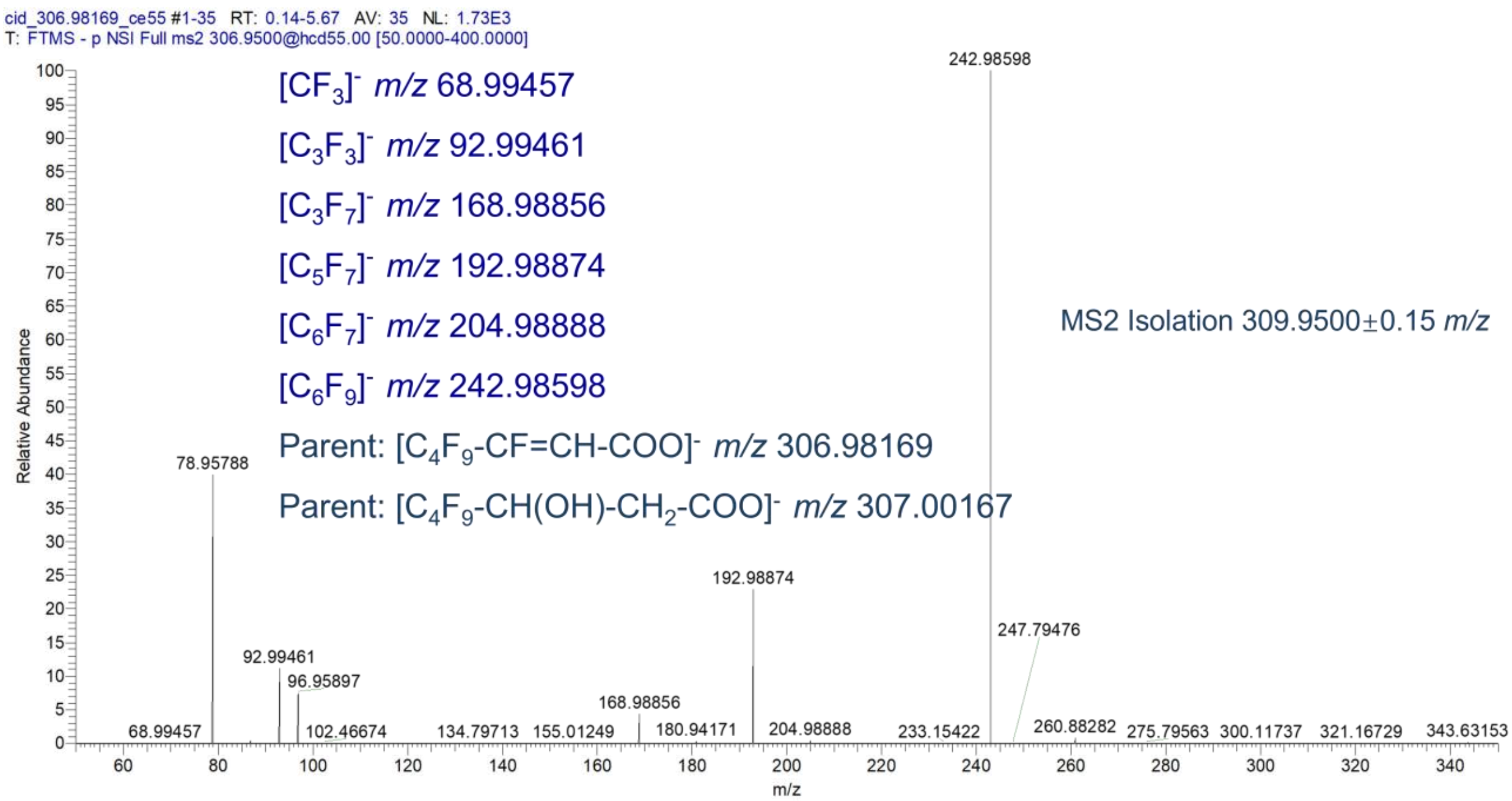

Figure M7. MS2 spectrum for isolation window of 306.95 ± 0.15 and its major product ions generated by CID.

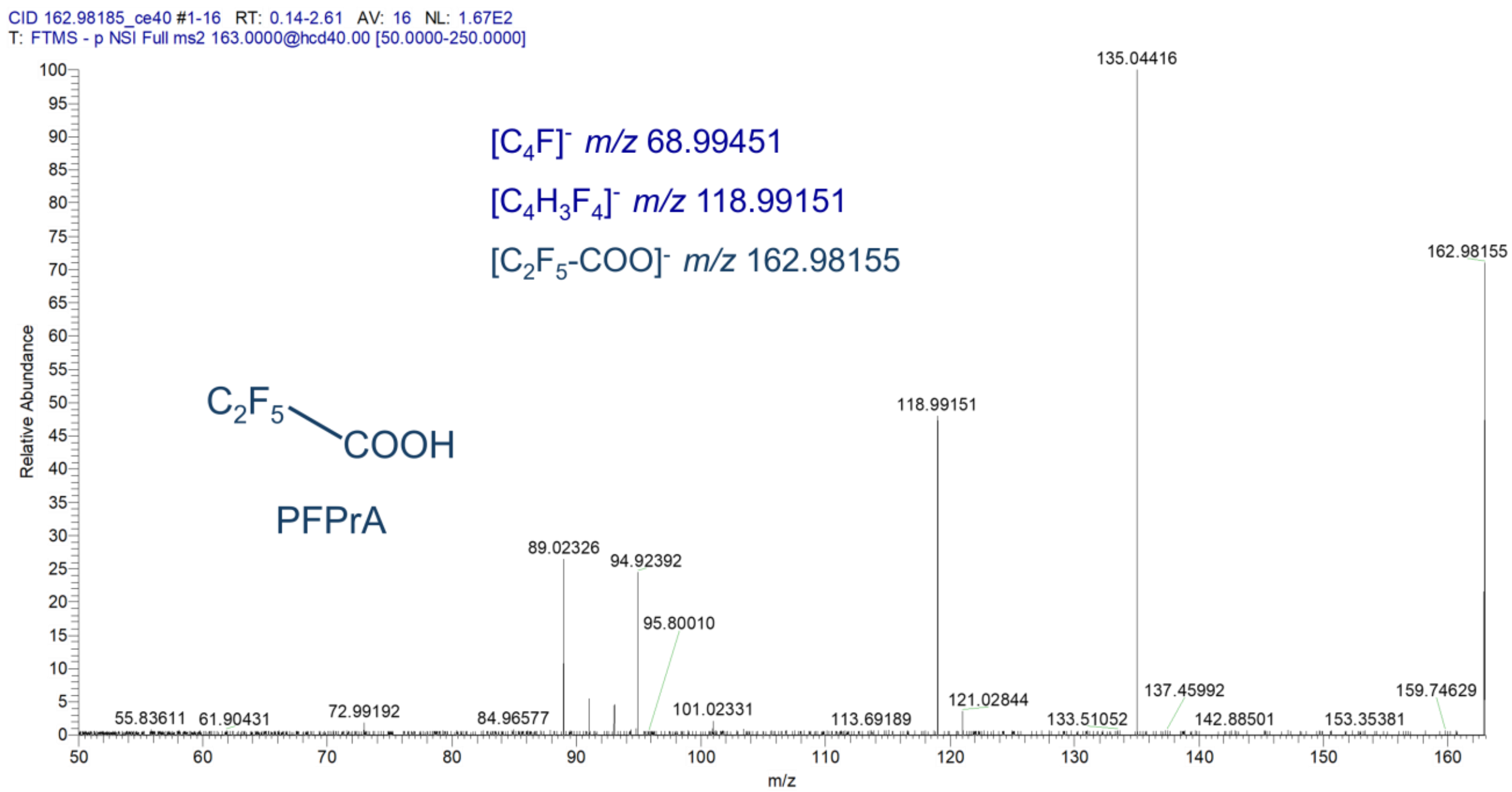

Figure M8. MS2 spectrum of PFPrA and its major product ions generated by CID.

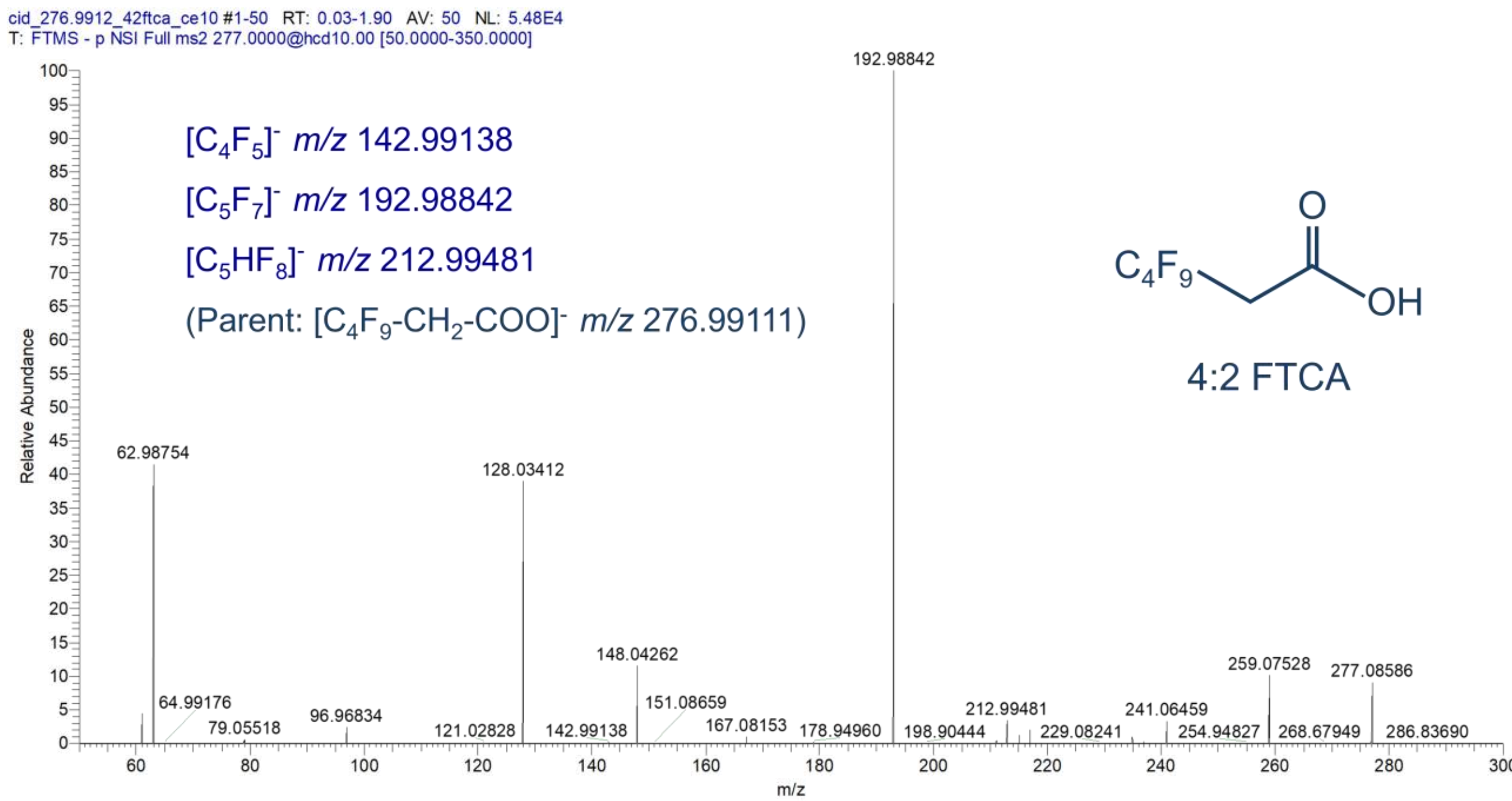

Figure M9. MS2 spectrum of 4:2 FTCA and its major product ions generated by CID (identification level 3d).

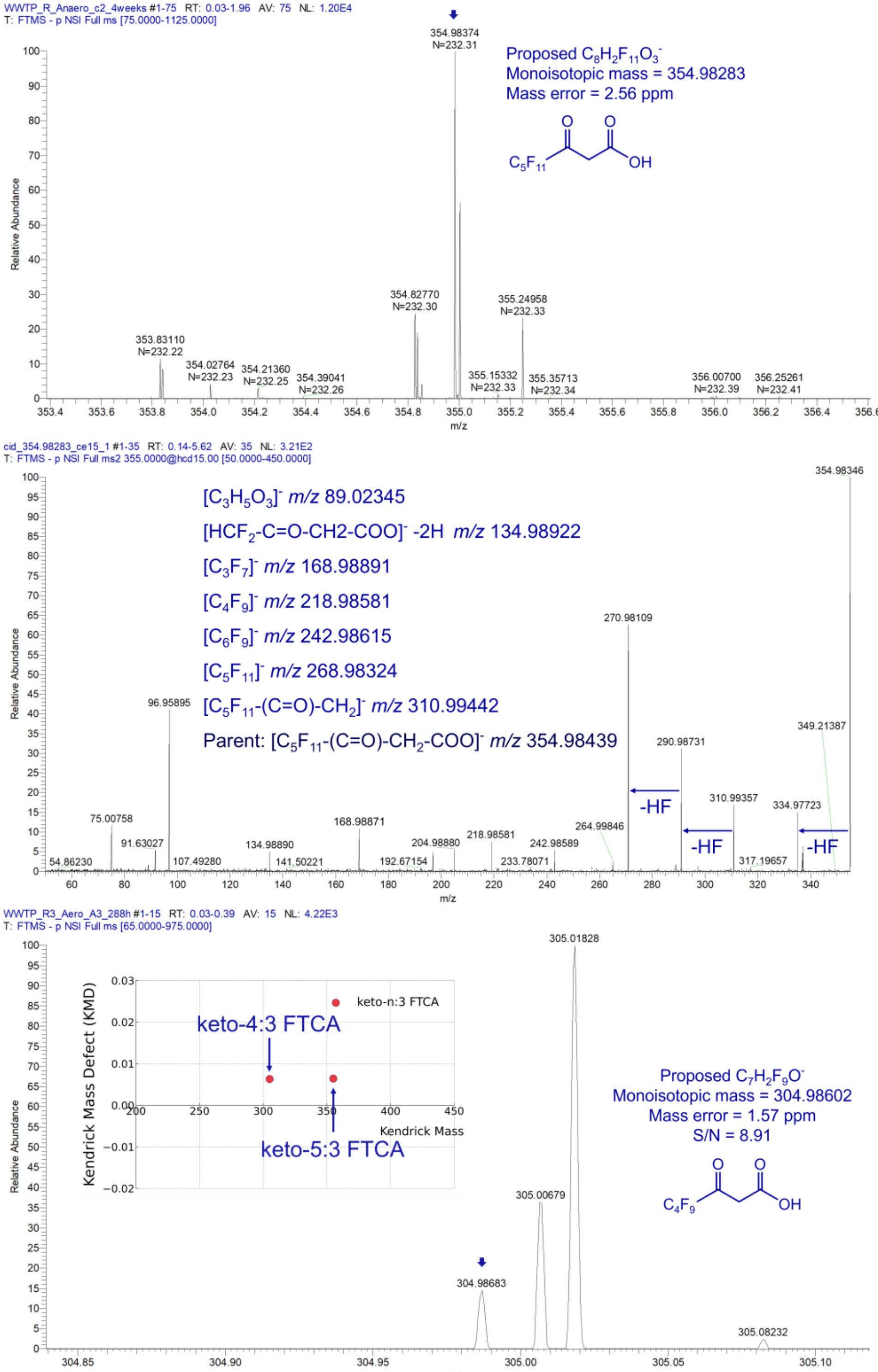

Figure M10. MS1&MS2 of keto-5:3 FTCA, MS1 of proposed keto-4:3 FTCA (identification level 3d), and Kendrick mass defect (KMD) of keto-5:3 FTCA and proposed keto-4:3 FTCA.

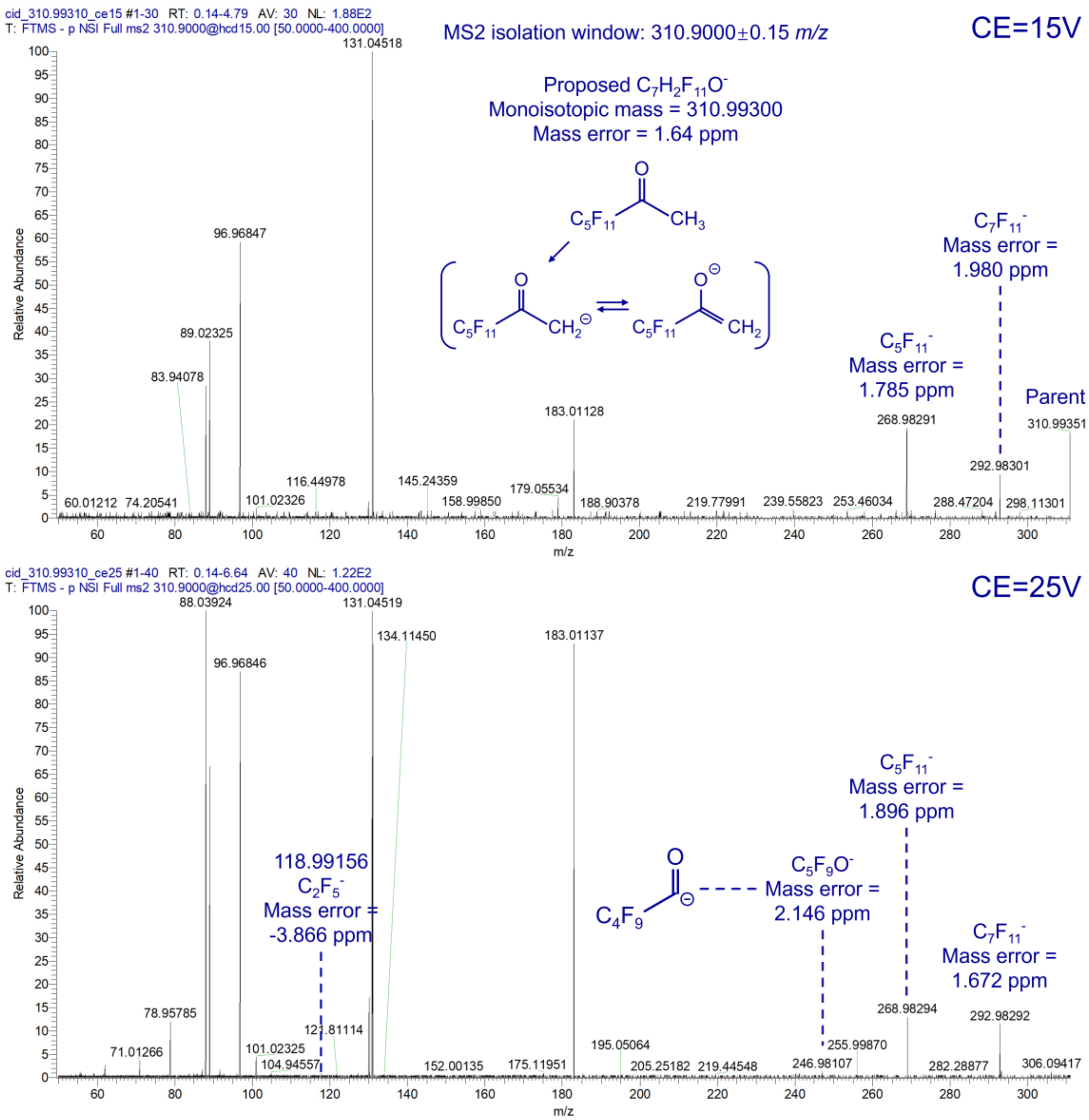

Figure M11. MS2 of proposed 5:2 FT ketone.

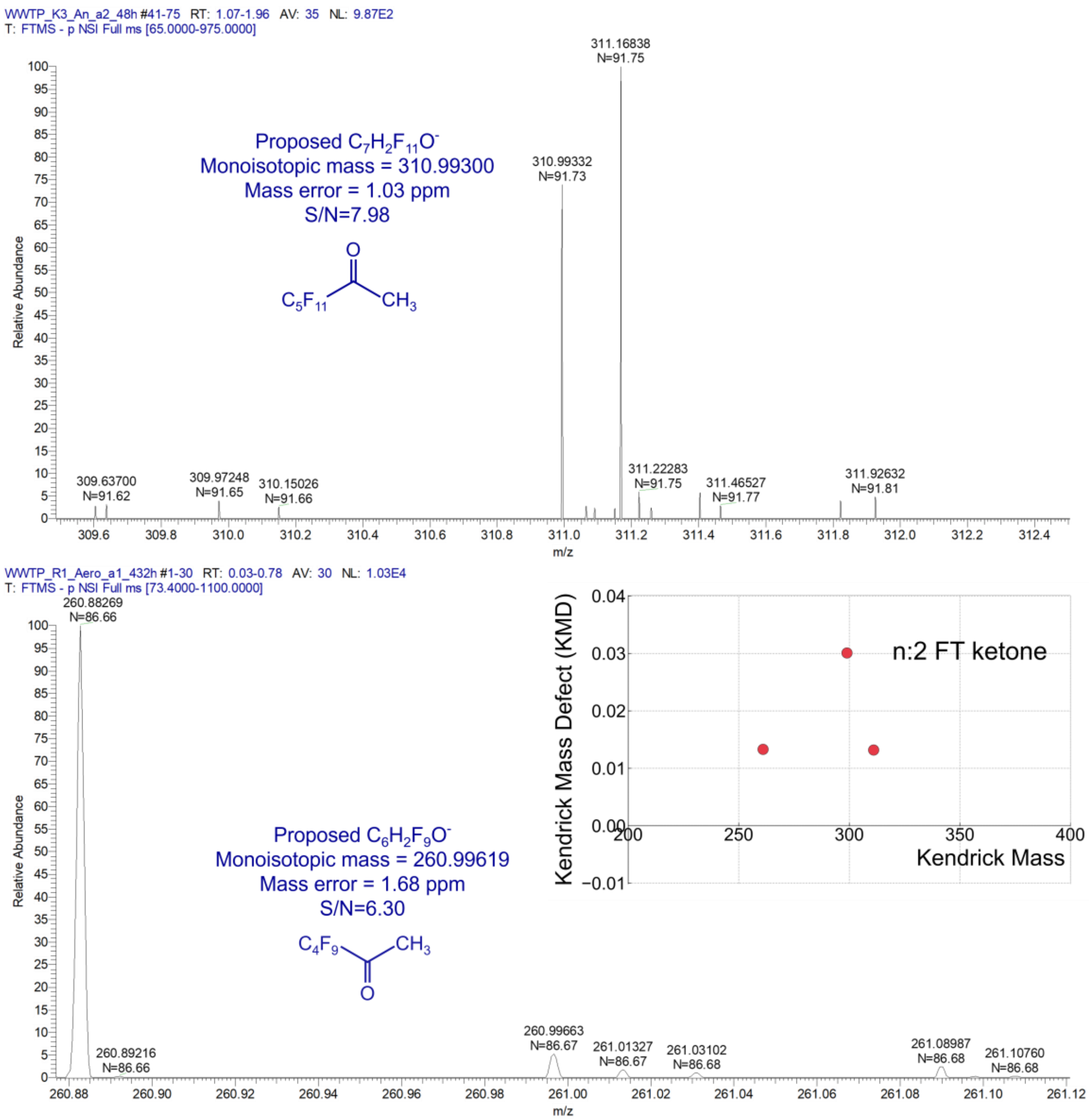

Figure M12. MS1 of proposed 5:2 FT ketone and 4:2 FT ketone, and KMD of 5:2 FT ketone and 4:2 FT ketone

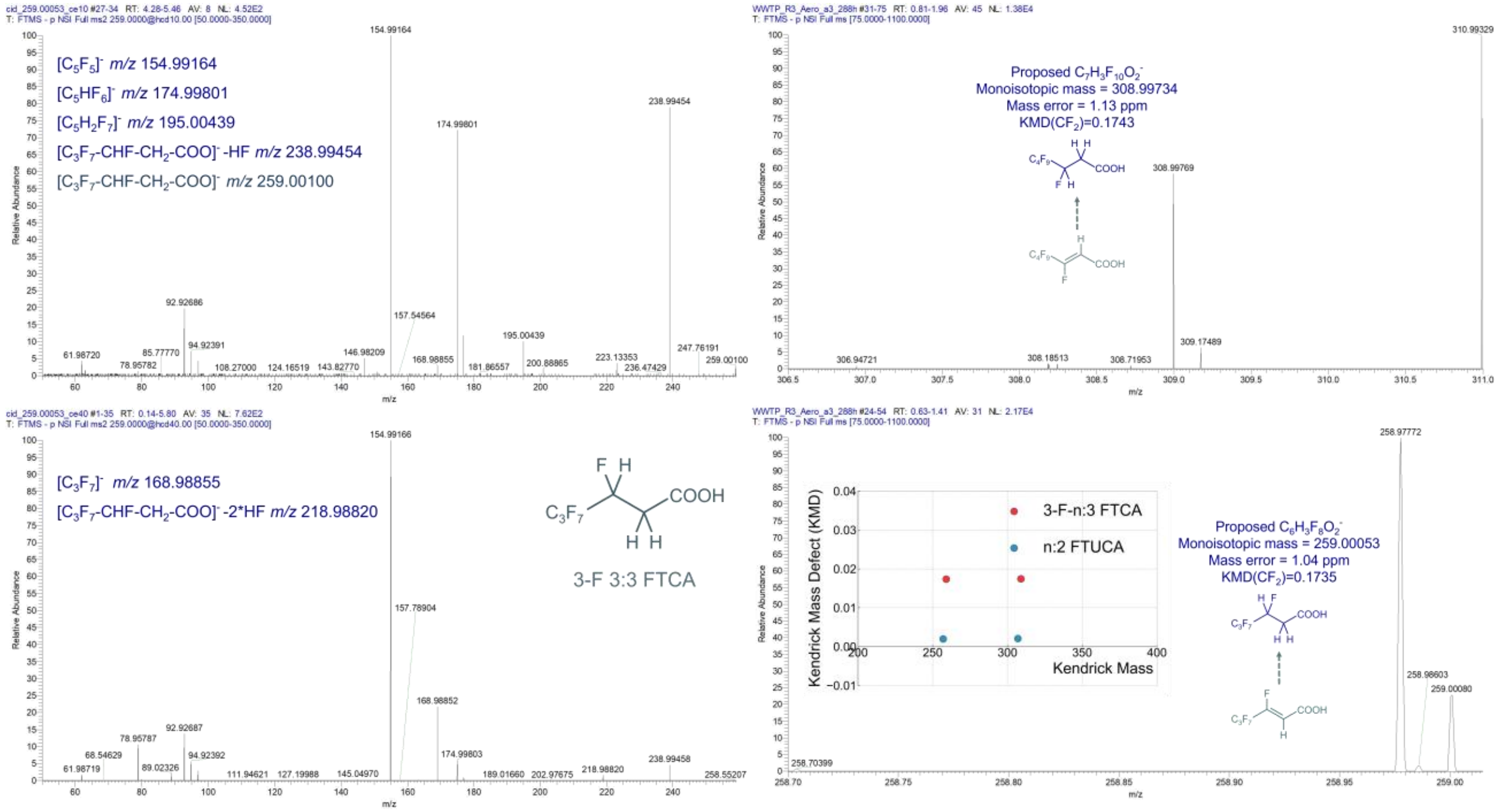

Figure M13. MS1&MS2 of 3-F 3:3 FTCA, MS1 of 3-F 4:3 FTCA, and KMD of 3-F 4:3 FTCA and 3-F 3:3 FTCA.

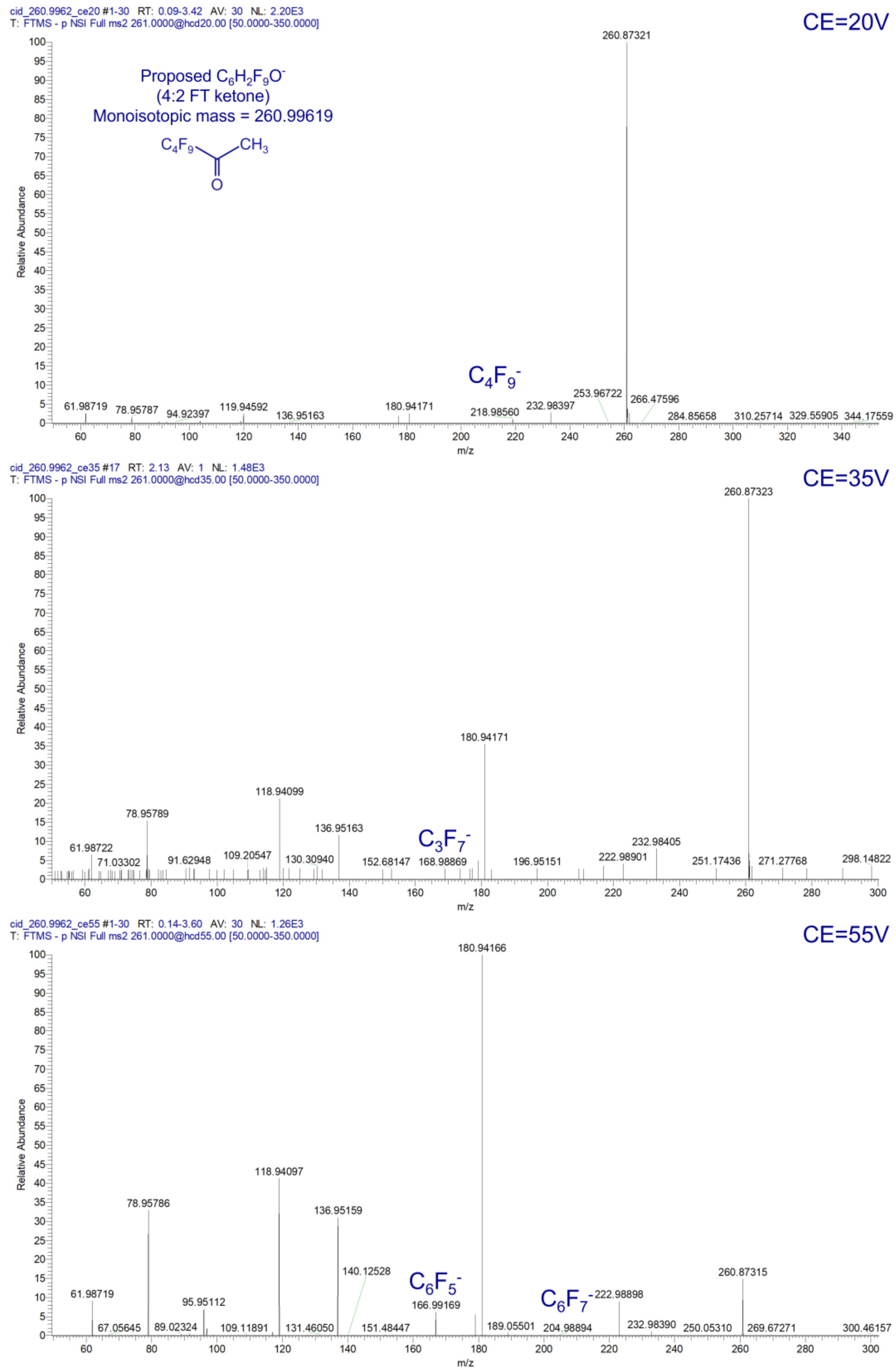

Figure M14. Fragments of proposed 4:2 FT ketone.
